# Identification of epidermal differentiation genes of the tuatara provides insights into the early evolution of lepidosaurian skin

**DOI:** 10.1038/s41598-020-69885-0

**Published:** 2020-07-30

**Authors:** Karin Brigit Holthaus, Lorenzo Alibardi, Erwin Tschachler, Leopold Eckhart

**Affiliations:** 1 Department of Dermatology, Medical University of Vienna, Vienna, Austria; 2 Comparative Histolab, Padova, Italy

**Keywords:** Evolutionary genetics, Evolutionary biology, Herpetology, Differentiation

## Abstract

The tuatara (*Sphenodon punctatus*) is the phylogenetically closest relative of squamates (including lizards and snakes) from which it diverged around 250 million years ago. Together, they constitute the clade Lepidosauria. Fully terrestrial vertebrates (amniotes) form their skin barrier to the environment under the control of a gene cluster, termed the epidermal differentiation complex (EDC). Here we identified EDC genes in the genome of the tuatara and compared them to those of other amniotes. The organization of the EDC and proteins encoded by EDC genes are most similar in the tuatara and squamates. A subcluster of lepidosaurian EDC genes encodes corneous beta-proteins (CBPs) of which three different types are conserved in the tuatara. Small proline-rich proteins have undergone independent expansions in the tuatara and some, but not all subgroups of squamates. Two genes encoding S100 filaggrin-type proteins (SFTPs) are expressed during embryonic skin development of the tuatara whereas SFTP numbers vary between 1 and 3 in squamates. Our comparative analysis of the EDC in the tuatara genome suggests that many molecular features of the skin that were previously identified in squamates have evolved prior to their divergence from the lineage leading to the tuatara.

## Introduction

The tuatara (*Sphenodon punctatus*) is the only living species of the order Rhynchocephalia which, together with the over 9.000-species rich order Squamata^1^, constitutes the superorder Lepidosauria. The evolutionary divergence of the two main lineages of lepidosaurs occurred 30 million years after the divergence of lepidosaurs and archelosaurs (including turtles, crocodilians, and birds), i.e. approximately 250 million years before present^2,3^. Lepidosaurs differ from archelosaurs and mammals with regard to the mechanism by which the superficial layers of the epidermis are renewed^4–6^. Uniquely, the epidermis of lepidosaurs grows cyclically and the outer generation of cells is shed either *in toto* or in pieces. Several histologically distinct layers of the epidermis (Oberhautchen, beta, mesos, alpha, lacunar, and clear layers) are formed during the skin shedding cycle in squamates^4–7^ whereas the layer organization of the epidermis is histologically less clearly defined in the tuatara^8–11^. In particular, the tuatara lacks histological equivalents of oberhautchen and clear layers which form the so-called shedding complex (responsible for molting) in squamates. These differences suggest that the tuatara may present a primitive epidermal condition in lepidosaurs, stressing the importance of this species as a study model for understanding the evolution of lepidosaurian skin.

Terrestrial vertebrates require protection against desiccation and environmental assaults. This protection is largely achieved by the outermost layer of the epidermis, the cornified layer, which consists of dead cells that are stabilized by extensive cross-linking of proteins. In mammals and birds, proteomes of these cornified cells (corneocytes) consist, to a significant extent, of proteins that are encoded by a cluster of genes, known as the epidermal differentiation complex (EDC)^12–14^. Two main types of EDC genes can be distinguished, (1) the so-called simple EDC (SEDC) genes which consist of a non-coding exon and another exon in which the entire coding sequence is located and (2) genes consisting of one non-coding and two coding exons, that together encode so-called S100 fused-type proteins or S100 filaggrin-type proteins (SFTPs)^15,16^. SEDC proteins such as loricrin, involucrin and small proline-rich proteins function predominantly, after cross-linking by transglutaminases, as resilient components of corneocytes^17–19^. By contrast, SFTPs undergo not only cross-linking but also limited proteolysis and furthermore interact with other proteins via their S100 domain and other motifs. Recently, the paradigmatic SFTP, filaggrin, was shown to form liquid-phase condensates, apparent as keratohyalin granules in histology^20^. Mutations in EDC genes are associated with skin barrier impairment and diseases in humans^21–24^ and changes of EDC genes are likely drivers of phenotypic diversification of the integument of tetrapods^14,25–30^. For example, subgroups of EDC genes were found to be associated in their evolution and expression pattern with the shell of turtles^25^, loricrin was detected in the epidermal α-(lacunar) layer but not in the β-layer of the anole lizard^31^, and epidermal differentiation cysteine-rich protein (EDCRP) was identified as a component of feather barbules in the chicken^32^. However, the great majority of EDC genes of non-mammalian tetrapods has not been investigated and the research into the associations between EDC genotype, skin phenotype and adaptations to the environment is still at its beginning.

The molecular architecture of the epidermis of the tuatara is only incompletely understood at present. A two-dimensional electrophoretic analysis indicated that at least 20 different corneous beta proteins (CBPs), previously termed beta-keratins, of 16–20 kilo-Dalton (kD) and a smaller number of keratin intermediate filament proteins of 40–63 kD, previously termed alpha-keratins, are expressed in the epidermis of the tuatara^33^. Partial amino acid sequences of 37 CBPs and the complete sequence of one CBP have been reported^34^ whereas the primary structure of other epidermal proteins of the tuatara is unknown. CBPs exist in reptiles and birds but not in amphibians and mammals^35^. They are characterized by the presence of a 34-amino acid sequence motif that assumes the conformation of a beta-sheet^36^. CBPs form dimers and fibers and their accumulation in differentiated keratinocytes contributes to the hardness and mechanical resilience of epidermal appendages such as scales in reptiles^10,37–40^. The only CBP for which a complete amino acid sequence has been reported in the tuatara^34^ corresponds in size to one of the CBPs detected by immunoblot analysis of epidermal extracts^33^ and, based on the analysis of its amino acid sequence, has an unusual structure. This CBP of the tuatara contains 4 beta-sheets and thereby resembles special orthologs in squamates^26,41^ but differs from the large majority of other CBPs in squamates and archelosaurs that have a single beta-sheet^35^. The high number of CBPs may be linked to the formation of diverse skin appendages, such as tuberculate scales, overlapped scales, dorsal and ventral spines and claws in the tuatara^11^. Besides CBPs, other proteins are likely to contribute to different types of shape and hardness of the skin appendages of the tuatara, but no specific data are available^37–39,41^.

In order to fill the gap of knowledge on the genes that determine the protein composition of the epidermis of Rhynchocephalia, the present study was carried out to identify EDC genes, including CBPs and SFTPs, in the whole genome shotgun sequence of the tuatara. Comparative analysis of the amino acid sequences encoded by these genes suggests features that are conserved among epidermal proteins of lepidosaurs and tuatara-specific molecular traits. The comprehensive analysis of EDC genes of both tuatara and squamates allows us to propose new models for the evolutionary origin and diversification of lepidosaurian EDC genes.

## Results

### Identification of the epidermal differentiation complex (EDC) in the tuatara genome

**Figure 1:**
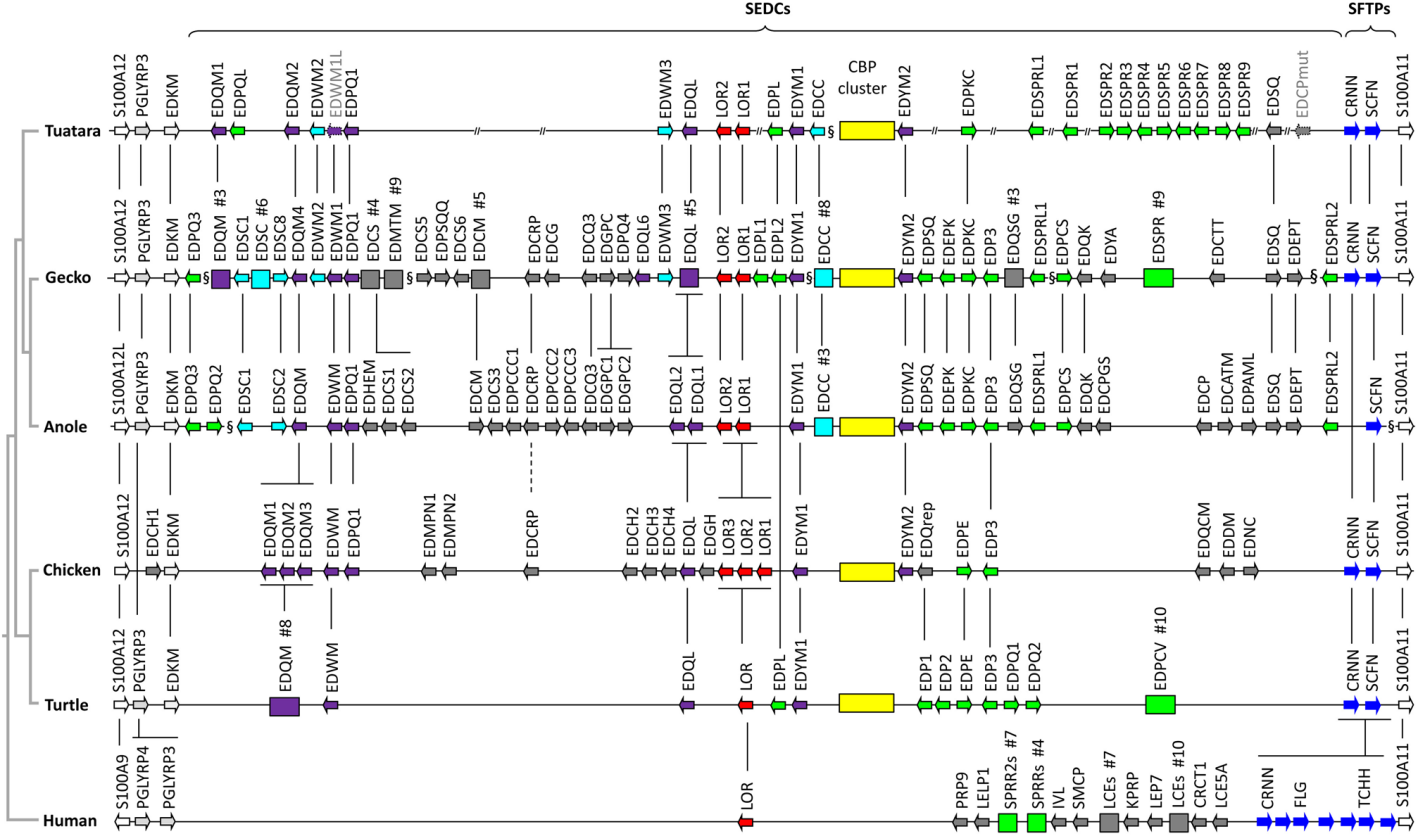
Organization of the epidermal differentiation complex (EDC) in the tuatara. Genes of the EDC locus of the tuatara (*Sphenodon punctatus*) are compared to those of the Japanese gecko (*Gekko japonicus*), anole lizard *(Anolis carolinensis),* chicken (*Gallus gallus*), turtle (*Chrysemys picta bellii*) and humans. Genes are displayed by arrows pointing in the direction of transcription. Rectangular boxes indicate clusters of similar genes with numbers of genes (#) in these cluster being shown above the boxes. Orthologies of genes and gene families in different species are indicated by vertical lines. EDC gene symbols of the tuatara are colored and genes encoding proteins of similar amino acid sequence in other species are shown in the same colors. The “§” symbols indicate gaps between genome sequence scaffolds. “//” symbols indicate gaps within scaffolds that cause uncertainty about the local gene arrangement. The gene map is not drawn to scale. Chicken genes *EDQM3* and *EDPQ1* correspond to *EDSC* and *EDCH5* , respectively, in a previous report^14^. A phylogenetic tree of the species is depicted on the left^1,2^. SEDC, simple EDC gene (1 coding exon); SFTP, S100 filaggrin-type protein.

To identify EDC genes in the draft genome of the tuatara (*Sphenodon punctatus*), we performed tBLASTn searches using EDC proteins of the green anole lizard^14^ as queries. Genome sequence scaffolds that contained EDC orthologs were further investigated and EDC genes were predicted de novo according to a protocol established in previous publications^14,25–27^ (Fig. 1). The same approach was applied to identify EDC genes of the Japanese gecko (*Gekko japonicus*)^42^, representing a major evolutionary branch of squamates (Suppl. Tables S1-S4). For the comparative analysis of some EDC genes, additional species were included (Suppl. Table S1).

Tentative names of EDC genes were assigned following the previously established nomenclature system (Suppl. Table S2)^14^ in which “Epidermal Differentiation” (ED) is followed by a description of amino acid features of the protein encoded by the respective gene. For instance, EDPKC stands for ED protein rich in proline (P), lysine (K) and cysteine (C). Members of gene families are distinguished by numbers at the end of the gene name. Orthologs of well-characterized EDC genes such as loricrin, cornulin, scaffoldin and CBPs were named to indicate this orthology. The full names of the EDC genes are listed in Supplementary Table S2 whereas only their abbreviations are used in the text. Gene positions on scaffolds and amino acid sequences of the proteins are shown in the Supplement (Suppl. Table S3, Suppl. Fig. S1). The expression of several EDC genes of the tuatara could be confirmed by the identification of sequence reads in an embryo transcriptome^43^ (Suppl. Fig. S2). Likewise, many newly predicted EDC genes of the Japanese gecko were validated by RNA-seq reads in a public database (Suppl. Table S4).

### The structure of the EDC is similar in the tuatara and in squamates

The EDC genes of the tuatara were present on 7 genome sequence scaffolds that could be aligned to the EDC gene maps of other reptiles (Fig. 1). For further investigations, the EDC of the tuatara was compared to those of two squamate species, i.e. the Japanese gecko (Suppl. Fig. S3 and Suppl. Table S4) and the green anole lizard (*A. carolinensis*), the chicken (*Gallus gallus*)^14^, a turtle (*Chrysemys picta bellii*)^25^, and human^15,44^. SEDC genes, including a cluster of CBP genes, form the main body of the EDC of the tuatara (Fig. 1). The cluster of SEDC genes is flanked by SFTP genes, i.e. *cornulin* and *scaffoldin*, and other genes with at least 2 coding exons, such as the EDKM, PGLYRP3, and S100As (Fig. 1).

Many of the tuatara EDC genes are 1:1 orthologs of squamate EDC genes (indicated by vertical lines in Fig. 1, Suppl. Fig. S4) but there are also 1-to-many orthology relationships between EDC genes of tuatara and squamates. For several EDC genes that are present in both gecko and anole lizard, we did not find an ortholog in the tuatara. This could be caused by genuine absence of these genes in the tuatara genome or by the incompleteness of the available genome sequence in GenBank at the time of this study (March 2020). The region between *EDPQ1* and *EDWM3* lacks genes in the tuatara whereas the syntenic region of the EDC contains several genes, in particular those encoding cysteine-rich proteins in squamates. Orthologs of *EDCRP* and neighboring genes of the gecko could not be found on any of the available scaffolds of the tuatara. Likewise, we did not find orthologs of *EDPQ3*, *EDPE*, and some other squamate EDC genes. *EDCC*, *EDPKC*, *EDSPRL1*, *EDCP* and *EDSQ* are present in the tuatara and squamates but not in archelosaurs^14,26^, suggesting that these genes originated either in a common ancestor of lepidosaurs after divergence from the archelosaurian lineage or in the sauropsid cenancestor followed by loss in archelosaurs. *EDSC*s are present in squamates but they are absent in the tuatara, suggesting an origin after the divergence of these lineages or loss in the tuatara lineage. *EDWM* which is conserved across sauropsids with the exception of softshell turtles^25^ is present in the form of 3 copies in the tuatara and the gecko whereas only one *EDWM* gene is present in the anole lizard, indicating gene loss in the latter (Suppl. Fig. S5).

### Tuatara EDC genes encode sequence repeat-rich proteins

**Figure 2:**
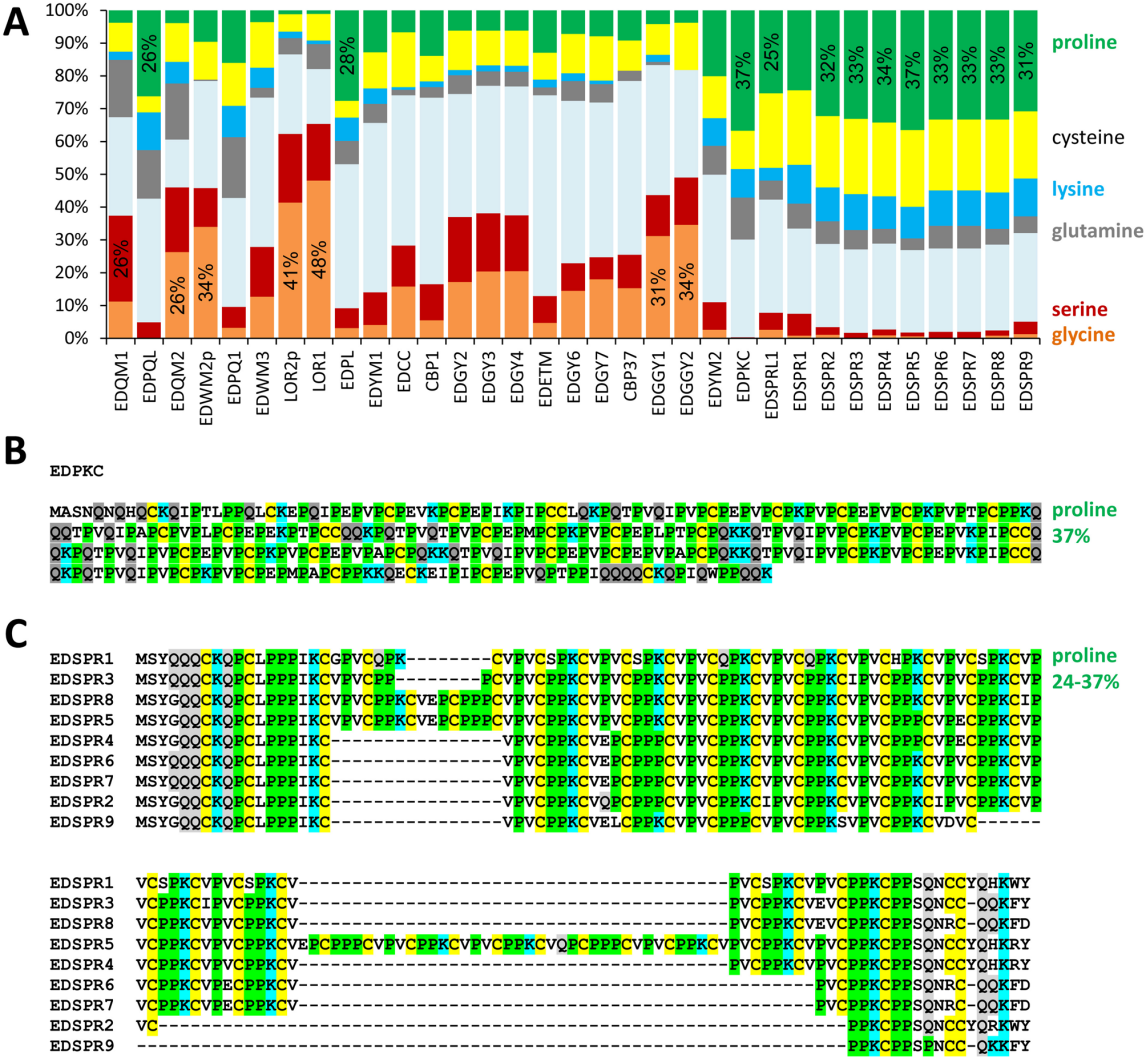
Tuatara EDC proteins are enriched in a small subset of amino acid residues. (**A**) The diagram shows the amino acid compositions of the tuatara SEDC proteins. The order of the protein data follows the order of genes on the EDC (Fig. 1). Among corneus beta proteins (CBPs), only those encoded by the first and the last gene of the CBP cluster are included in this analysis. (**B**) Amino acid sequence of tuatara epidermal differentiation protein rich in proline (P), lysine (K) and cysteine (C) (EDPKC). (**C**) Amino acid sequence alignment of epidermal differentiation small proline-rich proteins (EDSPRs) of the tuatara. Note the presence of repeated sequence motifs. Colors are used to highlight amino acid residues glutamine (Q), K (potential targets of transglutamination), C (potential sites of disulfide bonds), and P. EDWMp, EDWM partial sequence.

We translated the coding sequences of EDC genes of the tuatara and compared the resulting amino acid sequences to those of EDC proteins of squamates and other amniotes. Like their homologs, most tuatara EDC proteins exhibit high contents of one or several of the amino acids glycine, serine, proline and cysteine (Fig. 2A). Moreover, the amino acid residues glutamine and lysine are present in most of these proteins and may serve as targets of transglutamination^12^. While the biased amino acid composition of EDC proteins is largely caused by the repetitive nature of the amino acid sequences in the central part of the proteins, glutamine and lysine are predominantly present in conserved sequence motifs^14,26^ at the amino and carboxy-terminal segments (Fig. 2B,C).

Loricrins 1 and 2 have the highest contents of glycine and serine (together more than 60% of total amino acid residues), EDQM1 is glutamine and serine-rich (26%) (Suppl. Fig. S6) and high proline contents are found in EDPQL, EDPL, EDPKC (Fig. 2B) and ED small proline-rich (EDSPR) proteins (Fig. 2C; Suppl. Fig. S7).

### Small proline-rich proteins have increased in numbers in the tuatara

EDSPRs are a family of 9 proteins encoded by a gene cluster that is located close to the SFTP genes in the EDC of the tuatara. They contain up to 17 repeats of sequence motifs such as KCVPVCPP in EDSPR5 (Suppl. Fig. S7). A similar family of small proline-rich proteins (SPRRs) is expressed in mammalian keratinocytes where they are incorporated by transglutamination into the protein envelopes that form during cornification^45,46^.

**Figure 3:**
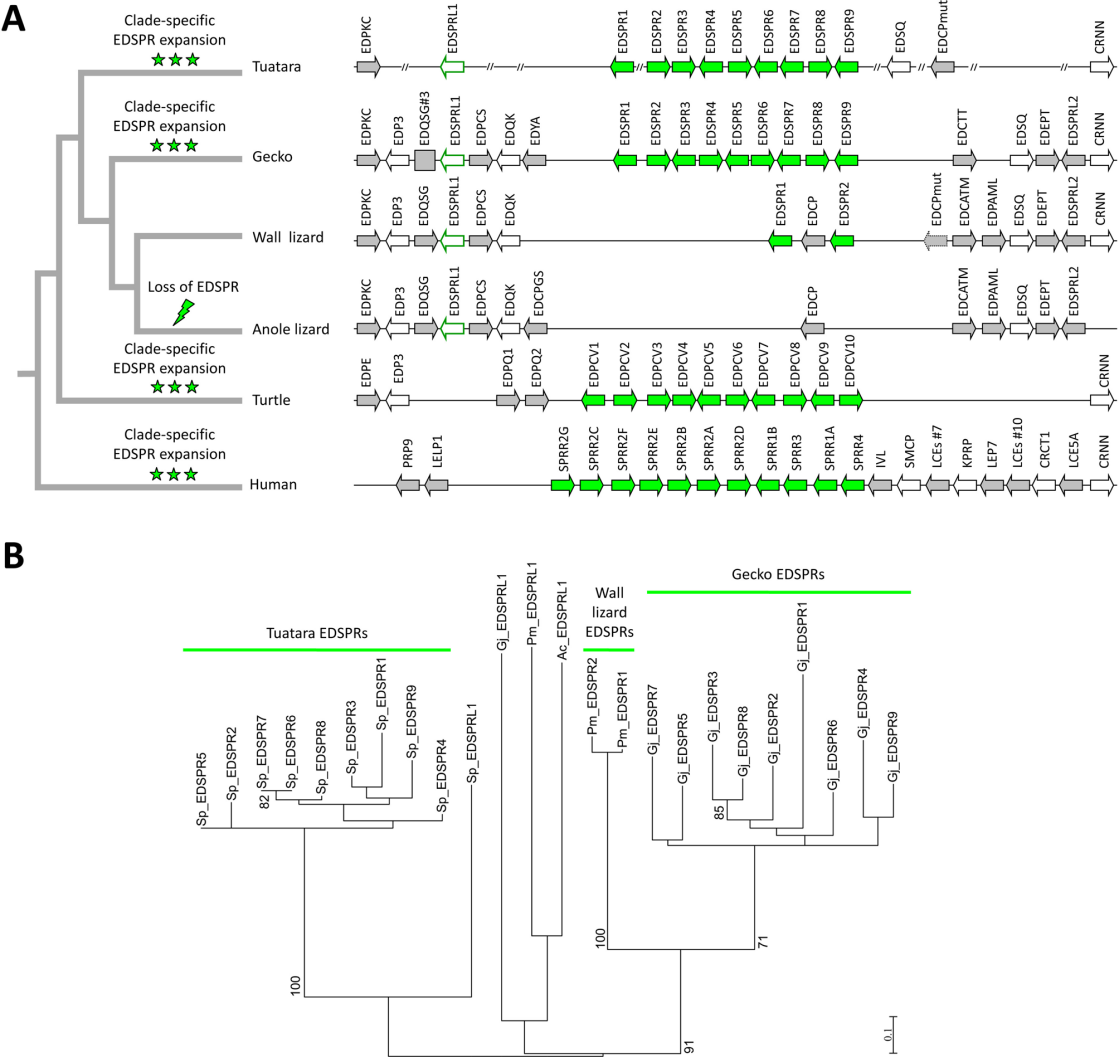
Small proline-rich proteins have increased in numbers during the evolution of the tuatara. (**A**) Schematic representation of the *EDSPR* gene cluster of the tuatara in comparison to clusters of small proline-rich protein (*EDSPR*, *EDPCV*, *SPRR*) gene clusters in other amniotes. Phylogenetic relationships of species are shown by the tree on the left. Expansion of proline-rich proteins occurred in various evolutionary lineages whereas *EDSPR* was lost in the anole lizard (indicated by a lightning bolt symbol on the tree). A phylogenetic tree of the species is depicted on the left^1,2^. (**B**) Phylogenetic analysis of lepidosaurian EDSPRs. A phylogenetic tree of lepidosaurian EDSPRs was generated by maximum likelihood analysis. Species: Tuatara (*Sphenodon punctatus*), Japanese gecko (*Gekko japonicus*), common wall lizard (*Podarcis muralis*), anole lizard (*Anolis carolinensis*), Western painted turtle (*Chrysemys picta bellii*), human (*Homo sapiens*).

EDSPR genes of the tuatara are located at a position that is largely syntenic with those of proline-rich protein genes in the gecko, wall lizard, turtles and humans (Fig. 3A), suggesting that they are orthologous. To estimate whether there are 1:1 orthology relationships of some of these genes, we performed a phylogenetic analysis. There was strong support for monophyly of tuatara EDSPR1-9 to the exclusion of gecko EDSPR1-9 and wall lizard EDSPR1-2, suggesting that the EDSPR gene cluster expanded independently in the different clades of lepidosaurs (Fig. 3B).

### Three types of CBPs have evolved in lepidosaurs

**Figure 4:**
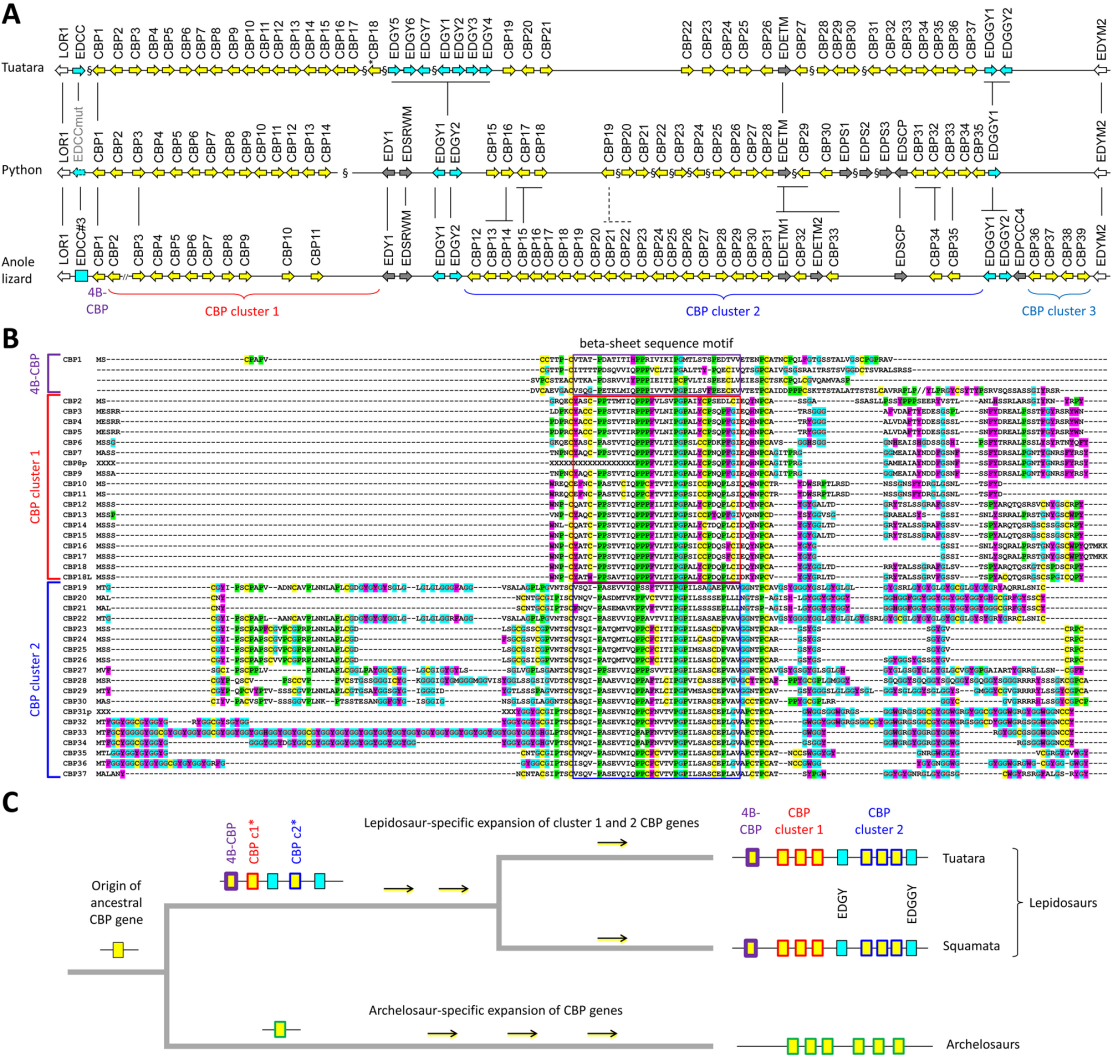
Three distinct types of CBPs have evolved in lepidosaurs. (**A**) Comparison of the corneous beta-protein (CBP) or beta-keratin gene clusters of the tuatara (*S. punctatus*), the anole lizard (*A. carolinensis*) and the python (*Python bivittatus*). The CBP cluster is flanked by *loricrin1* (*LOR1*) and *EDYM2*, while its continuity is interrupted by genes that encode proteins without beta-sheet motifs, such as *EDGY*, *EDGGY*, *EDETM*, and others. Presumed orthology is represented by black vertical lines. The symbol § indicates discontinuity in the genome sequence assembly. A sequence gap in a sequence scaffold of the anole lizard CBP cluster is indicated by the // symbol. The schematic is not drawn to scale. *, note that the loci of the genes encoding CBP18 and CBP18L are unknown and may be outside of the EDC. Only the hypothetical locus of the *CBP18* gene besides genes of high sequence similarity is indicated. (**B**) Amino acid sequence alignment of tuatara CBP genes. The 4 beta-sheet sequence motifs of CBP1 are aligned to the single beta-sheet sequence motifs of the other CBPs. A selected set of amino acid residues is highlighted by colors (cysteine, yellow; proline, green; glycine, blue with red fonts; aromatic residues, magenta). (**C**) A hypothesis for the origin and evolution of lepidosaur CBP genes based on the species distribution of CBP1, cluster 1 and cluster 2 CBP genes, and non-CBP genes.

Among reptilian SEDC proteins, CBPs stand out by the presence of a beta-sheet sequence motif that facilitates protein dimerization and by their massive abundance as compared to other epidermal proteins of sauropsids^35,36,38,39,47^. The complete amino acid sequence of one CBP and 37 amino acid sequence fragments possibly corresponding to CBPs of the tuatara were reported previously^34^. Here, we could determine the complete sequences of 36 CBPs, both at the gene and protein levels, and partial sequences of 2 CBPs of the tuatara (Fig. 4; Suppl. Fig. S1B). We also found several CBP pseudogenes which correspond at the nucleotide sequence level to some of the predicted amino acid sequence fragments^34^. Due to the incomplete assembly of the tuatara genome, we could not determine the location of all CBP genes relative to other genes, but 2 genome sequence scaffolds containing CBP genes (GenBank accession numbers QEPC01001131.1 and QEPC01003505.1) also included non-CBP genes, that have orthologs in the EDCs of squamates (Fig. 4A; Suppl. Table S3).

Despite the uncertainty about the arrangement of EDC sequence scaffolds, we can estimate the number of CBP genes in the EDC of the tuatara (n = 36–40) to be similar to those of the python (n = 35) and the green anole lizard (n = 40) (Fig. 4A) while this number is smaller than the number of CBPs of geckos^26,34,42,48^. The continuity of the CBP gene cluster is interrupted by non-CBP genes, namely *EDETM* and *EDGY* genes (Fig. 4). Essentially the same organization of the CBP gene locus is found in all squamates investigated so far^26^. In the course of this comparison, we found previously unknown orthologs of the tuatara *EDGGY1* and *EDGGY2* genes in squamates (Fig. 4; Suppl. Fig. S8).

Among the CBP proteins of the tuatara, only one CBP with a complete amino acid sequence was described previously and this one has 4 beta-sheet sequence motifs, like the 4-beta-sheet-CBPs (4B-CBPs) of squamates^26,34^. All additional CBPs identified in the present study have one beta-sheet region (Fig. 4B), thus resembling the classical CBPs of reptiles and birds^36,38,47,49^. The CBP genes are arranged in two separate clusters within the EDC of the tuatara (Fig. 4A). Likewise, a syntenic organization of CBPs genes is present in the EDC of squamates. The cluster 1 of CBP genes is located between the 4B-CBP gene and the *EDGY* genes whereas cluster 2 genes are located on the other side of the *EDGY* genes (Fig. 4A). A third CBP gene cluster is present in the anole lizard but not in the python and the tuatara (Fig. 4A). Molecular phylogenetics provided support for monophyly of cluster 1 CBPs of tuatara and squamates and for monophyly of lepidosaurian CBPs to the exclusion of archelosaurian CBPs (Suppl. Fig. S9-S10; Suppl. Table S5).

In the light of the phylogeny of the tuatara and other reptiles^1–3^, these findings suggest the following evolutionary model for CBPs in lepidosaurs (Fig. 4C). All lepidosaurian CBPs evolved from a single CBP present in the common ancestor of sauropsids. Gene duplications and sequence mutations led to the origin of three types of CBPs (4B-CBP, cluster 1 and cluster 2 CBPs) in the common ancestor of lepidosaurs already more than 250 million years ago. While 4B-CBP remained a single-copy gene in all lepidosaurs investigated, cluster 1 and cluster 2 CBPs evolved differentially in the various clades of lepidosaurs, thus contributing to the phenotypic diversification of the scaled skin and its diverse derivatives such as claws, pads, frills and others.

### A single scaffoldin gene is expressed in the tuatara whereas two scaffoldins are present in multiple lineages of squamates

**Figure 5:**
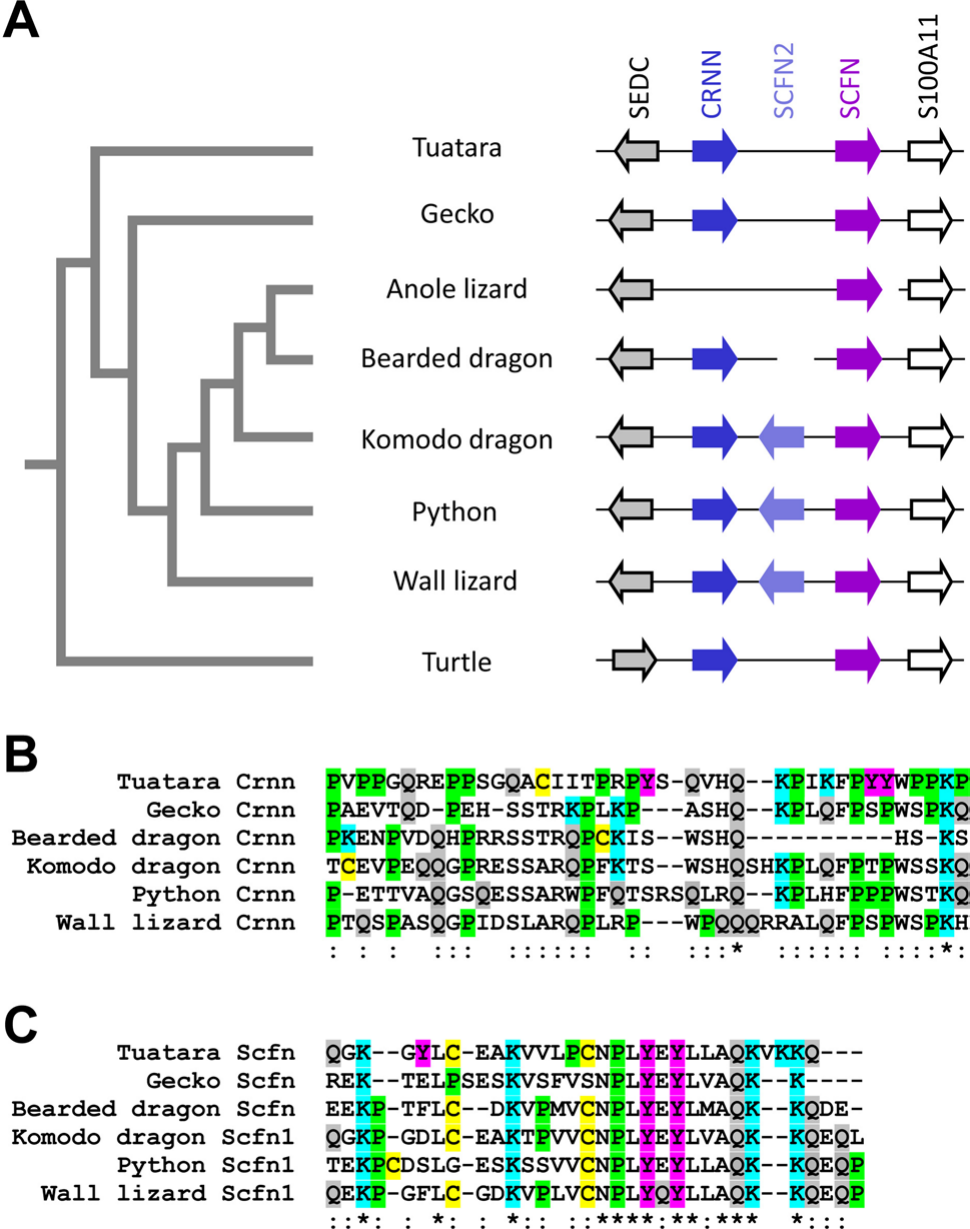
A single scaffoldin gene is present in the tuatara whereas two scaffoldins are present in multiple lineages of squamates. (**A**) Comparison of the scaffoldin (SCFN) loci in phylogenetically diverse lepidosaurs, namely tuatara (*S. punctatus*), gecko (*G. japonicus*), anole lizard (*A. carolinensis*), bearded dragon (*Pogona vitticeps*), Komodo dragon (*Varanus komodoensis*), python (*Python bivittatus*), wall lizard (*Podarcis muralis*) and, representing an outgroup, a turtle (*C. picta bellii*). A phylogenetic tree of the species is depicted on the left^1,2^. Amino acid sequence alignments of the carboxy-termini of cornulin (Crnn) (**B**) and scaffoldin 1 (Scfn 1) (**C**). Positions with identical amino acid residues in all or more than 50% of the sequences are indicated by “*” and “:”, respectively, below the sequences. Colors highlight the same set of amino acid residues as in Fig. 2. In addition, tyrosine (Y) is highlighted in magenta.

The two SFTP genes, *cornulin* (*Crnn*) and *scaffoldin* (*Scfn*), are present at the end of the EDC of the tuatara and both genes are expressed during embryonic development (Suppl. Fig. S2). This is compatible with the hypothesis of evolutionarily conserved roles of these SFTPs in the periderm of sauropsids^50^. However, only *Scfn* but not *Crnn* is conserved in the anole lizard^14^ and two copies of *Scfn* are located close to *Crnn* in snakes^26^. To further investigate the evolution of SFTPs in lepidosaurs, we analyzed the SFTP gene locus in the EDCs of several lineages of squamates such as geckos (Japanese gecko, *Gekko japonicus*), agamids (bearded dragon, *Pogona vitticeps*), varanids (Komodo dragon, *Varanus komodoensis*), snakes (python, *Python bivittatus*), and lacertids (wall lizard, *Podarcis muralis*) (Fig. 5A). *Crnn* was found in all squamates investigated, except the anole lizard and all of the encoded cornulin proteins had a characteristic carboxy-terminal sequence motif that distinguishes cornulin from other SFTPs (Fig. 5B)^50^. At least one *Scfn* gene was identified in all squamates and a second copy, named *Scfn2*, was found not only in snakes but also in the Komodo dragon and in the wall lizard (Fig. 5A). In all these species, *Scfn2* had the opposite orientation as *Scfn1* and only Scfn1 proteins contained a carboxy-terminal sequence motif (Fig. 5C). This motif was previously identified in similar form in other SFTPs, including human filaggrin and trichohyalin^16,26^. By contrast, the carboxy-termini of Scfn2 proteins lacked the motifs present in Crnn or Scfn1 and their sequences were not conserved among Scfn2 proteins of different species (Suppl. Fig. S11). Interestingly, there are stretches of high intra-specific sequence similarity and even up to 100% identity in Scfn1 and Scfn2 proteins of the same species (Suppl. Fig. S11, indicating either recent independent duplications of the *Scaffoldin* gene in three lineages (varanids, snakes, and lacertids) or duplication of *Scfn* in a common ancestor, followed by gene conversion between *Scfn1* and *Scfn2* in varanids, snakes, and lacertids and loss of Scfn2 in iguanids.

## Discussion

The results of this study establish the first catalog of epidermal differentiation genes of the tuatara and thereby help to address open questions pertaining to the skin barrier of the tuatara and to the molecular evolution of epidermal differentiation in lepidosaurs. We identified more than 50 tuatara genes located in the EDC, a gene cluster that originated during the evolution of fully terrestrial life of amniotes^14^ and was inherited, with clade-specific modifications, in all modern species of amniotes investigated so far^14,25–27,44^. In addition, we identified EDC genes of a gecko species and other squamates with incompletely annotated genomes. This allowed us to compare the EDC genes of the tuatara and other amniotes and to obtain the following main findings which will be subsequently discussed in detail: (1) The EDC of the tuatara is most similar, but not identical in gene composition, to the EDC of squamates. (2) A group of small proline-rich proteins has expanded by gene duplications in the tuatara. (3) Besides the previously characterized CBP with 4 beta-sheets, two types of CBPs are present in the tuatara. (4) Two SFTPs are present in the tuatara whereas squamates have one, two or three SFTPs.

1. The majority of genes in the EDC of the tuatara have orthologs in squamates, suggesting high similarity in the molecular composition of the skin barrier in all lepidosaurs which is in line with morphological and biochemical data^8–10,33^. Several EDC genes, such as *cornulin*, *scaffoldin*, *loricrin*, *EDWM*, *EDKM*, *EDQM*, *EDQL* and *EDYM1*, are shared between the tuatara and all main clades of sauropsids, indicating that evolutionarily ancient epidermal differentiation genes are conserved and functional in the tuatara. The lepidosaur-specific conserved genes include *EDCC*, *EDETM*, *EDGY* and *EDGGY* (Fig. 1). However, other genes, such as *EDSC*, *EDCRP*, *EDPSQ*, *EDEPK*, *EDQSG*, *EDQK*, *EDEPT*, *EDSPRL2*, and *EDP3* that are present in the EDC of squamates, could not be identified in the tuatara. Due to incompleteness of the tuatara genome sequence currently available in GenBank, the lack of detection of these genes cannot be considered as evidence for absence and further studies are necessary to ascertain which ones, if any, mark molecular differences between the tuatara and squamates. At present it is unknown which genetic differences cause the phenotypic differences of the skin in lepidosaurs.
2. EDSPR genes are clustered in the EDC of the tuatara and, based on the results of our phylogenetic analysis, have evolved by repeated duplications that occurred after the divergence from the squamate lineage. This expansion of proline-rich proteins in the tuatara is remarkable because very similar expansions of proline-rich proteins have occurred during the evolution of geckos (Fig. 3), turtles^25^ and mammals^14,15,44^. Human and mouse proline-rich EDC proteins, known as SPRRs, function as important components of the cornified envelope of epidermal keratinoytes^12,45^. Through their cysteine residues, they also act as quenchers of reactive oxygen species^51^. It appears plausible that the EDSPRs of the tuatara play similar roles but conclusions about their function require experimental tests in future studies. The conservation of small proline-rich proteins in the tuatara and many other amniotes suggests that these proteins have been important for the evolution of epidermal cornification.
3. Thirty-five complete and two incomplete CBP coding sequences were identified in the tuatara besides the CBP gene, that was previously reported as the only complete CBP gene of the tuatara^34^. CBPs are the quantitatively predominant components of sauropsidian epidermis^35,36,38–40,47^. Here we present new phylogenetic data on lepidosaurian CBPs which suggest that the newly described CBPs of the tuatara as well as 1-beta-sheet CBPs of squamates can be classified into two types. Sequence differences in the central beta-motif and the outer protein segments suggest differences in the modes of interaction with other proteins. Tuatara CBPs of type 1 (encoded by CBP cluster 1 genes) have only a short amino-terminal domain with 7–10 amino acid residues before the beta-sheet motif whereas CBPs of type 2 have amino-terminal domains with up to 100 residues (Fig. 4B).
4. The tuatara has two SFTPs, i.e. cornulin and scaffoldin, and in that regard resembles archelosaurs and geckos (Fig. 5), whereas it differs from other squamates such as the anole lizard (1 SFTP), snakes, Komodo dragon and the wall lizard (3 SFTPs). The implications of differences in SFTPs content on skin biology are not yet known. However, our finding that both *cornulin* and *scaffoldin* are expressed during embryonic development of the tuatara (Suppl. Fig. S2) suggests that they play similar roles in the cornification of the periderm as their orthologs in the chicken periderm^50^. Morphological and ultrastructural studies have indeed detected coarse granules, likely equivalent to periderm granules of birds, in the cornifying periderm of tuatara embryos^11^.

The characterization of EDC genes of the tuatara provides the basis for determining the in situ expression patterns of these genes and for investigating the functions of the corresponding proteins in vivo and in experimental systems in vitro. Together with the comparative analysis of the EDC in phylogenetically diverse squamates, these future studies will define the epidermal barrier of the tuatara and unravel the evolutionary history of lepidosaurian skin.

## Methods

### Gene identification in genome sequences

EDC genes were identified in the genome sequences of the tuatara (*Sphenodon punctatus*) (GenBank accession number QEPC00000000.1, submitted by Rutherford KM and Gemmell NJ, Department of Anatomy, University of Otago, Dunedin, New Zealand) and the Japanese gecko (*Gekko japonicus*)^42^ (Suppl. Table S1). Genomes of several other lepidosaurs, such as the bearded dragon (*Pogona vitticeps*)^52^, the common wall lizard (*Podarcis muralis*)^53^ and Komodo dragon (*Varanus komodoensis*)^54^ were analyzed for the gene content of specific regions within their EDC. The accession numbers of EDC genes of the tuatara and the gecko are listed in Supplementary Tables S3 and S4, respectively.

To identify EDC genes of the tuatara and the gecko, we performed tBLASTn searches with EDC protein sequences of the green anole lizard (*Anolis carolinensis*) as queries^14,55^. In the tBLASTn searches, the filter for low-complexity regions was deactivated whereas default settings of the NCBI browser were used for other parameters. Amino acid sequences were obtained by the translation of open reading frames in the regions found by tBLASTn, followed by comparison with EDC proteins identified in other lepidosaurian species^14,25–27^. Subsequently, the genome sequence scaffolds containing EDC genes were further investigated by in silico translation of the entire nucleotide sequence and by screening the translation products for sequence motifs characteristic of EDC proteins as defined previously^14^. The sequences of these de novo-predicted proteins were then used as queries to search for similar proteins in the same and in other species and the predicted coding sequences were investigated for the presence of splice sites in the pattern that is characteristic for SEDC and SFTP genes^14^. Orthology of genes was assigned using the criteria of best reciprocal BLAST hits and gene locus synteny.

The expression of tuatara EDC genes was validated by tBLASTn searches in the transcriptome of an early stage tuatara embryo^43^. Most of the predicted gecko (*G. japonicus*) genes were confirmed to be expressed by the alignment of RNA-seq reads (from tissues and regenerating tail, including epidermis)^42^ in the NCBI browser for “genomic regions, transcripts, and products” (https://www.ncbi.nlm.nih.gov/gene/, last accessed on March 11, 2020) (Supplementary Table S4).

### Analysis of amino acid sequences encoded by EDC genes

Amino acid sequences were aligned with MUSCLE and phylogenetic analyses were performed with PhyML at the Seaview platform^56^. The protocol for the phylogenetic analysis of CBP sequences was modified from a previous study^25^ using unambiguously aligned sequences of the beta-motifs and the JTT model for maximum likelihood analysis with 100 bootstrap replicates. To highlight particular sequence motifs and repeats in Figs. 2C, 4B, 5B,C, S4-S8, and S11B-D, amino acid sequences were aligned with MultAlin followed by manual optimization^57^. The ProtParam software tool at the ExPASy portal (https://web.expasy.org/protparam/, last accessed on March 31, 2020) was used to calculate amino acid percentages of EDC gene-encoded proteins^58^.

## Supplementary information

Supplementary information.
