# Supplementary material for: Identification of epidermal differentiation genes of the tuatara provides insights into the early evolution of lepidosaurian skin
Source: Sci Rep. 2020 Jul 30;10:12844. doi: 10.1038/s41598-020-69885-0 (PMC7393497; doi:10.1038/s41598-020-69885-0)
Supplement: Supplementary file 1 — Supplementary information. [file 41598_2020_69885_MOESM1_ESM.pdf]

Supplementary Data: Supplementary Tables and Figures

Identification of epidermal differentiation genes of the tuatara provides insights into the early evolution of lepidosaurian skin

Karin Brigit Holthaus, Lorenzo Alibardi, Erwin Tschachler, Leopold Eckhart

Content

Supplementary Tables S1-S5

Supplementary Figures S1-S11

Supplementary Table S1. Sauropsid species investigated in this study

Species	Abbr.	Common name	Systematic classification	Genome assembly accession (GenBank)	Results
Sphenodon punctatus	Sp	Tuatara	Sauropsida; Lepidosauria; Rhynchocephalia; Sphenodontia; Sphenodontidae	GCA_003113815.1	Fig. 1-5; Suppl. Fig. S1, S2, S4-S11
Gekko japonicus	Gj	Japanese gecko	Sauropsida; Lepidosauria; Squamata; Gekkota; Gekkonidae	GCA_001447785.1	Fig. 1, 3, 5; Suppl. Fig. S3-S8, S11
Podarcis muralis	Pm	Common wall lizard	Sauropsida; Lepidosauria; Squamata; Laterata; Lacertidae	GCA_004329235.1	Fig. 3, 5; Suppl. Fig. S4, S5, S7, S8, S11
Python bivittatus	Pb	Burmese python	Sauropsida; Lepidosauria; Squamata; Serpentes; Pythonidae	GCF_000186305.1	Fig. 4, 5; Suppl. Fig. S5, S6, S8, S10, S11
Ophiophagus hannah	Oh	King cobra	Sauropsida; Lepidosauria; Squamata; Serpentes; Elapidae	GCA_000516915.1	Suppl. Fig. S11
Varanus komodoensis	Vk	Komodo dragon	Sauropsida; Lepidosauria; Squamata; Anguimorpha; Varanidae	GCA_007859595.1	Fig. 5; Suppl. Fig. S11
Anolis carolinensis	Ac	Green anole lizard	Sauropsida; Lepidosauria; Squamata; Iguania; Dactyloidae	GCF_000090745.1	Fig. 1, 3-5; Suppl. Fig. S4-S8, S11
Pogona vitticeps	Pv	Bearded dragon	Sauropsida; Lepidosauria; Squamata; Iguania; Agamidae	GCA_900067755.1	Fig. 5; Suppl. Fig. S11
Chrysemys picta bellii	Cp	Western painted turtle	Sauropsida; Archelosauria; Testudines; Cryptodira; Emydidae	GCA_000241765.1	Fig. 1, 3, 5; Suppl. Fig. S5, S7, S11
Alligator sinensis	As	Chinese alligator	Sauropsida; Archelosauria; Archosauria; Crocodylia; Alligatoridae	GCF_000455745.1	Suppl. Fig. S5, S7, S10
Gallus gallus	Gg	Chicken	Sauropsida; Archelosauria; Archosauria; Aves; Phasianidae	GCA_000002315.3	Fig. 1; Suppl. Fig. S5-S7

Abbr., abbreviation

Supplementary Table S2. Tentative abbreviations and full names of EDC genes (tuatara, gecko)

Gene abbreviation	Full gene name
CRNN	Cornulin
EDCC	Epidermal Differentiation protein containing Cysteine Cysteine motifs
EDCC2	Epidermal Differentiation protein containing Cysteine Cysteine motifs 2
EDCC3	Epidermal Differentiation protein containing Cysteine Cysteine motifs 3
EDCC4	Epidermal Differentiation protein containing Cysteine Cysteine motifs 4
EDCC5	Epidermal Differentiation protein containing Cysteine Cysteine motifs 5
EDCC6	Epidermal Differentiation protein containing Cysteine Cysteine motifs 6
EDCC7	Epidermal Differentiation protein containing Cysteine Cysteine motifs 7
EDCC8	Epidermal Differentiation protein containing Cysteine Cysteine motifs 8
EDCG	Epidermal Differentiation protein rich in Cysteine and Glycine repeats
EDCM	Epidermal Differentiation protein containing a CCCC Motif
EDCP	Epidermal Differentiation protein rich in Cysteine and Proline
EDCQ3	Epidermal Differentiation protein rich in Cysteine and glutamine (Q) 3
EDCRP	Epidermal Differentiation Cysteine-Rich Protein
EDCS1	Epidermal Differentiation protein, Cysteine-rich Short 1
EDCS2	Epidermal Differentiation protein, Cysteine-rich Short 2
EDCS3	Epidermal Differentiation protein, Cysteine-rich Short 3
EDCS4	Epidermal Differentiation protein, Cysteine-rich Short 4
EDCS5	Epidermal Differentiation protein, Cysteine-rich Short 5
EDCS6	Epidermal Differentiation protein, Cysteine-rich Short 6
EDCTT	Epidermal Differentiation protein containing Cysteine and Threonine (T) duplets
EDEPK	Epidermal Differentiation protein rich in glutamic acid (E), Proline and lysine (K)
EDEPT	Epidermal Differentiation protein rich in glutamic acid (E), Proline and Threonine
EDETM	Epidermal Differentiation protein containing an ET Motif
EDGPC	Epidermal Differentiation protein rich in Glycine, Proline and Cysteine
EDGY1	Epidermal Differentiation protein rich in Glycine and tyrosine (Y) 1
EDGY2	Epidermal Differentiation protein rich in Glycine and tyrosine (Y) 2
EDGY3	Epidermal Differentiation protein rich in Glycine and tyrosine (Y) 3
EDGY4	Epidermal Differentiation protein rich in Glycine and tyrosine (Y) 4
EDGY5	Epidermal Differentiation protein rich in Glycine and tyrosine (Y) 5
EDGY6	Epidermal Differentiation protein rich in Glycine and tyrosine (Y) 6
EDGY7	Epidermal Differentiation protein rich in Glycine and tyrosine (Y) 7
EDGGY1	Epidermal Differentiation protein rich in a Glycine duplet and tyrosine (Y) 1
EDGGY2	Epidermal Differentiation protein rich in a Glycine duplet and tyrosine (Y) 2
EDGGY3	Epidermal Differentiation protein rich in a Glycine duplet and tyrosine (Y) 3
EDKM	Epidermal Differentiation protein containing a KKLIQQ Motif
EDMTM1	Epidermal Differentiation protein containing a MTVLC Motif 1
EDMTM2	Epidermal Differentiation protein containing a MTVLC Motif 2
EDMTM3	Epidermal Differentiation protein containing a MTVLC Motif 3
EDMTM4	Epidermal Differentiation protein containing a MTVLC Motif 4
EDMTM5	Epidermal Differentiation protein containing a MTVLC Motif 5
EDMTM6	Epidermal Differentiation protein containing a MTVLC Motif 6
EDMTM7	Epidermal Differentiation protein containing a MTVLC Motif 7
EDMTM8	Epidermal Differentiation protein containing a MTVLC Motif 8
EDMTM9	Epidermal Differentiation protein containing a MTVLC Motif 9
EDP3	Epidermal Differentiation protein rich in Proline 3
EDPCS	Epidermal Differentiation protein rich in Proline, Cysteine and Serine

Supplementary Table S2. Tentative abbreviations and full names of EDC genes (tuatara, gecko) - continued

Gene abbreviation	Full gene name
EDPKC	Epidermal Differentiation protein rich in Proline, lysine (K) and Cysteine
EDPL	Epidermal Differentiation Proline-rich protein, close to Loricrin
EDPL2	Epidermal Differentiation Proline-rich protein, close to Loricrin 2
EDPQ1	Epidermal Differentiation protein rich in Proline and glutamine (Q) 1
EDPQ3	Epidermal Differentiation protein rich in Proline and glutamine (Q) 3
EDPQ4	Epidermal Differentiation protein rich in Proline and glutamine (Q) 4
EDPQL	Epidermal Differentiation protein rich in Proline and glutamine (Q) Like
EDPSQ	Epidermal Differentiation protein rich in Proline, Serine and glutamine (Q)
EDPSQQ	Epidermal Differentiation protein with Proline, Serine and glutamine (QQ) repeats
EDQK	Epidermal Differentiation protein containing glutamine (Q) and lysine (K) repeats
EDQL	Epidermal Differentiation protein rich in glutamine (Q), close to Loricrin
EDQL2	Epidermal Differentiation protein rich in glutamine (Q), close to Loricrin 2
EDQL3	Epidermal Differentiation protein rich in glutamine (Q), close to Loricrin 3
EDQL4	Epidermal Differentiation protein rich in glutamine (Q), close to Loricrin 4
EDQL5	Epidermal Differentiation protein rich in glutamine (Q), close to Loricrin 5
EDQL6	Epidermal Differentiation protein rich in glutamine (Q), close to Loricrin 6
EDQM1	Epidermal Differentiation protein containing a glutamine (Q) Motif 1
EDQM2	Epidermal Differentiation protein containing a glutamine (Q) Motif 2
EDQM3	Epidermal Differentiation protein containing a glutamine (Q) Motif 3
EDQM4	Epidermal Differentiation protein containing a glutamine (Q) Motif 4
EDQSG1	Epidermal Differentiation protein rich in glutamine (Q), Serine and Glycine 1
EDQSG2	Epidermal Differentiation protein rich in glutamine (Q), Serine and Glycine 2
EDQSG3	Epidermal Differentiation protein rich in glutamine (Q), Serine and Glycine 3
EDSC1	Epidermal Differentiation protein rich in Serine and Cysteine 1
EDSC2	Epidermal Differentiation protein rich in Serine and Cysteine 2
EDSC3	Epidermal Differentiation protein rich in Serine and Cysteine 3
EDSC4	Epidermal Differentiation protein rich in Serine and Cysteine 4
EDSC5	Epidermal Differentiation protein rich in Serine and Cysteine 5
EDSC6	Epidermal Differentiation protein rich in Serine and Cysteine 6
EDSC7	Epidermal Differentiation protein rich in Serine and Cysteine 7
EDSC8	Epidermal Differentiation protein rich in Serine and Cysteine 8
EDSCP1	Epidermal Differentiation protein rich in Serine, Cysteine and Proline
EDSCP2	Epidermal Differentiation protein rich in Serine, Cysteine and Proline
EDSPR1	Epidermal Differentiation protein Small Proline-Rich 1
EDSPR2	Epidermal Differentiation protein Small Proline-Rich 2
EDSPR3	Epidermal Differentiation protein Small Proline-Rich 3
EDSPR4	Epidermal Differentiation protein Small Proline-Rich 4
EDSPR5	Epidermal Differentiation protein Small Proline-Rich 5
EDSPR6	Epidermal Differentiation protein Small Proline-Rich 6
EDSPR7	Epidermal Differentiation protein Small Proline-Rich 7
EDSPR8	Epidermal Differentiation protein Small Proline-Rich 8
EDSPR9	Epidermal Differentiation protein Small Proline-Rich 9
EDSPRL1	Epidermal Differentiation protein Small Proline-Rich Like 1
EDSPRL2	Epidermal Differentiation protein Small Proline-Rich Like 2
EDSQ	Epidermal Differentiation protein rich in Serine and glutamine (Q)
EDSRWM	Epidermal Differentiation protein containing a SRW Motif
EDWM1	Epidermal Differentiation protein containing a WYDP Motif 1
EDWM2	Epidermal Differentiation protein containing a WYDP Motif 2

Supplementary Table S2. Tentative abbreviations and full names of EDC genes (tuatara, gecko) - continued

Gene abbreviation	Full gene name
EDWM3	Epidermal Differentiation protein containing a WYDP Motif 3
EDY1	Epidermal Differentiation protein rich in tyrosine (Y)
EDYA	Epidermal Differentiation protein containing tyrosine (Y) and Arginine repeats
EDYM1	Epidermal Differentiation protein containing Y Motif 1
EDYM2	Epidermal Differentiation protein containing Y Motif 2
LOR1	Loricrin 1
LOR2	Loricrin 2
PGLYRP3	Peptidoglycan recognition protein 3
SCFN	Scaffoldin

Note - Corneous beta protein (CBP) and S100A genes are not included here.

Supplementary Table S3. Tuatara (*Sphenodon punctatus*) EDC genes

Gene	Accession nr.	CDS start	CDS end	Sequence complete
S100-A12	QEPC01012061.1	>327511	324481	no
PGLYRP3	QEPC01012061.1	307674	300149	yes
EDKM	QEPC01012061.1	290186	286886	yes
EDQM1	QEPC01012061.1	271438	271668	yes
EDPQL	QEPC01012061.1	264883	265068	yes
EDQM2	QEPC01012061.1	254657	254899	yes
EDWM2	QEPC01012061.1	238115	>239737	no
EDPQ1	QEPC01012061.1	211366	212307	yes
EDWM3	QEPC01012061.1	177121	176624	yes
EDQL	QEPC01012061.1	166187	>166213	no
LOR2	QEPC01012061.1	158713	>159444	no
LOR1	QEPC01012061.1	122516	123073	yes
EDPL	QEPC01012061.1	34557	34853	yes
EDYM1	QEPC01012061.1	25201	25719	yes
EDCC	QEPC01012061.1	2219	1860	yes
CBP1	QEPC01002958.1	2749	1709	yes
CBP2	QEPC01002958.1	28787	28503	yes
CBP3	QEPC01002958.1	53258	53545	yes
CBP4	QEPC01002958.1	65137	65424	yes
CBP5	QEPC01002958.1	74330	74617	yes
CBP6	QEPC01002958.1	102651	102944	yes
CBP7	QEPC01002958.1	121772	121485	yes
CBP8	QEPC01002958.1	>134761	134537	no
CBP9	QEPC01002958.1	135188	134901	yes
CBP10	QEPC01002958.1	145723	145478	yes
CBP11	QEPC01002958.1	157608	157363	yes
CBP12	QEPC01002958.1	176541	176254	yes
CBP13	QEPC01002958.1	239708	239986	yes
CBP14	QEPC01002958.1	245661	245374	yes
CBP15	QEPC01002958.1	255361	255648	yes
CBP16	QEPC01002958.1	264774	264520	yes
CBP17	QEPC01002958.1	272231	272482	yes
CBP18	QEPC01003457.1	4019627	4019340	yes
CBP18L	QEPC01013643.1	15701	15988	yes
EDGY5	QEPC01007544.1	14123	14374	yes
EDGY6	QEPC01007544.1	28612	28878	yes
EDGY7	QEPC01007544.1	>40703	40581	no
EDGY1	QEPC01003505.1	11477	10899	yes
EDGY2	QEPC01003505.1	32583	32921	yes
EDGY3	QEPC01003505.1	53528	53866	yes
EDGY4	QEPC01003505.1	>57295	57411	no
CBP19	QEPC01003505.1	71695	72171	yes
CBP20	QEPC01003505.1	87094	86771	yes
CBP21	QEPC01003505.1	98304	98627	yes
CBP22	QEPC01003505.1	110862	111362	yes

Supplementary Table S3. Tuatara (*Sphenodon punctatus*) EDC genes (continued)

Gene	Accession nr.	CDS start	CDS end	Sequence complete
CBP23	QEPC01003505.1	144339	144653	yes
CBP24	QEPC01003505.1	155288	154977	yes
CBP25	QEPC01003505.1	163798	164112	yes
CBP26	QEPC01003505.1	172773	172432	yes
EDETM	QEPC01003505.1	217487	217744	yes
CBP27	QEPC01003505.1	237060	236590	yes
CBP28	QEPC01013324.1	3245	3736	yes
CBP29	QEPC01013324.1	15615	15130	yes
CBP30	QEPC01013324.1	42364	42771	yes
CBP31	QEPC01001131.1	>3846	3529	no
CBP32	QEPC01001131.1	17978	17556	yes
CBP33	QEPC01001131.1	44136	43597	yes
CBP34	QEPC01001131.1	54845	55264	yes
CBP35	QEPC01001131.1	71324	71028	yes
CBP36	QEPC01001131.1	92309	92656	yes
CBP37	QEPC01001131.1	119640	119936	yes
EDGGY1	QEPC01001131.1	137172	137462	yes
EDGGY2	QEPC01001131.1	144429	144115	yes
EDYM2	QEPC01001131.1	172060	171239	yes
EDPKC	QEPC01001131.1	237333	238364	yes
EDSPRL1	QEPC01001131.1	273831	273367	yes
EDSPR1	QEPC01001131.1	317720	317361	yes
EDSPR2	QEPC01001131.1	379248	379511	yes
EDSPR3	QEPC01001131.1	388954	389310	yes
EDSPR4	QEPC01001131.1	395148	394813	yes
EDSPR5	QEPC01001131.1	402542	403045	yes
EDSPR6	QEPC01001131.1	409963	409655	yes
EDSPR7	QEPC01001131.1	412620	412312	yes
EDSPR8	QEPC01001131.1	421099	421479	yes
EDSPR9	QEPC01001131.1	426718	426482	yes
EDSQ	QEPC01001131.1	>447305	447135	no
CRNN	QEPC01001131.1	519440	521923	yes
SCFN	QEPC01001131.1	546214	556831	no
S100-A11	QEPC01001131.1	<568743	570694	no

Notes - The symbols < and > indicate that ends of the coding sequence are not present on the scaffold. Only the S100A genes flanking PGLYRP3 and SCFN are shown here. CDS, coding sequence.

Supplementary Table S4. Japanese gecko (*Gekko japonicus*) EDC genes (other than CBPs)

Gene	Accession nr.	CDS start	CDS end	Sequence complete	Expression confirmed by RNA-seq data *
S100A12	NW_015164746.1	66175	70505	yes	yes
PGLYRP3	NW_015164746.1	110271	122296	yes	yes
EDKM	NW_015164746.1	150707	153538	yes	yes
EDPQ3	NW_015164746.1	197536	197306	yes	yes
EDQM1	NW_015167143.1	9742	9987	yes	yes
EDQM2	NW_015167143.1	20664	20419	yes	yes
EDQM3	NW_015167143.1	33175	33426	yes	yes
EDSC1	NW_015167143.1	37688	37065	yes	yes
EDSC2	NW_015167143.1	59253	58948	yes	yes
EDSC3	NW_015167143.1	72180	72440	yes	yes
EDSC4	NW_015167143.1	78673	78368	yes	yes
EDSC5	NW_015167143.1	91642	91947	yes	yes
EDSC6	NW_015167143.1	117418	117113	yes	yes
EDSC7	NW_015167143.1	128817	128530	yes	yes
EDSC8	NW_015167143.1	167957	168223	yes	yes
EDQM4	NW_015167143.1	174574	174332	yes	yes
EDWM2	NW_015165872.1	29756	28887	yes	yes
EDWM1	NW_015165872.1	42769	42116	yes	yes
EDPQ1	NW_015165872.1	82896	82288	yes	yes
EDCS1	NW_015165872.1	118514	118900	yes	yes
EDCS2	NW_015165872.1	136718	136945	yes	yes
EDCS3	NW_015165872.1	151174	151401	yes	yes
EDCS4	NW_015165872.1	175698	175964	yes	yes
EDMTM1	NW_015165872.1	187813	188025	yes	no
EDMTM2	NW_015165872.1	211098	211310	yes	yes
EDMTM3	NW_015165872.1	222370	222582	yes	yes
EDMTM4	NW_015165872.1	234077	234256	yes	yes
EDMTM5	NW_015165872.1	242734	242573	yes	yes
EDMTM6	NW_015165872.1	259695	259504	yes	yes
EDMTM7	NW_015165872.1	284784	284599	yes	yes
EDMTM8	NW_015165872.1	302829	302638	yes	yes
EDMTM9	NW_015165872.1	319521	319336	yes	yes
EDCS5	NW_015165273.1	13829	14191	yes	yes
EDPSQQ	NW_015165273.1	32839	42051	no	no
EDCS6	NW_015165273.1	60383	60021	yes	no
EDCM1	NW_015165273.1	94837	94616	yes	yes
EDCM2	NW_015165273.1	109303	109524	yes	yes
EDCM3	NW_015165273.1	117819	117598	yes	yes
EDCM4	NW_015165273.1	139633	139409	yes	yes
EDCM5	NW_015165273.1	163634	163413	yes	yes
EDCRP	NW_015165273.1	187728	184328	no	yes
EDCG	NW_015165273.1	203419	203240	yes	no
EDCQ3	NW_015165273.1	222104	221946	yes	no
EDGPC	NW_015165273.1	284184	284396	yes	yes
EDPQ4	NW_015165273.1	306777	307295	yes	yes
EDQL6	NW_015165273.1	311324	311034	yes	yes
EDWM3	NW_015165273.1	324050	324457	yes	no
EDQL5	NW_015165273.1	337173	336925	yes	yes
EDQL4	NW_015165273.1	348116	347760	yes	yes
EDQL3	NW_015165273.1	367896	367540	yes	yes
EDQL2	NW_015165273.1	380357	380001	yes	yes
EDQL	NW_015165273.1	397419	396856	yes	yes
LOR2	NW_015165273.1	449402	448278	yes	yes

Supplementary Table S4. Japanese gecko (*Gekko japonicus*) EDC genes (other than CBPs) (continued)

Gene	Accession nr.	CDS start	CDS end	Sequence complete	Expression confirmed by RNA-seq data *
LOR1	NW_015165273.1	497029	494960	yes	yes
EDPL1	NW_015165273.1	515159	514992	yes	yes
EDPL2	NW_015165273.1	562108	561893	yes	no
EDYM1	NW_015165273.1	575957	575454	yes	no
EDCC1	NW_015164736.1	243196	242813	yes	no
EDCC2	NW_015164736.1	237427	237810	yes	no
EDCC3	NW_015164736.1	227203	227586	yes	no
EDCC4	NW_015164736.1	217712	217329	yes	no
EDCC5	NW_015164736.1	199680	199892	yes	no
EDCC6	NW_015164736.1	172305	171769	yes	no
EDCC7	NW_015160458.1	<506	568	no	n.a.
EDCC8	NW_015160458.1	>8213	8133	no	n.a.
EDY1	NW_015174878.1	279730	279398	yes	yes
EDSRWM	NW_015174878.1	325455	326549	yes	yes
EDGY1	NW_015174878.1	342767	343264	yes	yes
EDGY2	NW_015174878.1	354050	354553	yes	yes
EDGY3	NW_015174878.1	361077	360646	yes	yes
EDSCP1	NW_015169269.1	32772	34046	yes	no
EDETM	NW_015160848.1	797526	797272	yes	yes
EDSCP2	NW_015160848.1	58604	59641	yes	no
EDGGY1	NW_015160848.1	3647	3441	yes	no
EDGGY2	NW_015166463.1	405198	405461	yes	no
EDGGY3	NW_015166463.1	366321	365956	yes	yes
EDYM2	NW_015166463.1	238411	239349	yes	yes
EDPSQ	NW_015166463.1	218291	217020	yes	no
EDEPK	NW_015166463.1	182229	182723	yes	no
EDPKC	NW_015166463.1	110127	108682	yes	yes
EDP3	NW_015166463.1	92756	93076	yes	yes
EDQSG1	NW_015166463.1	79330	79758	yes	yes
EDQSG2	NW_015166463.1	65051	64677	yes	yes
EDQSG3	NW_015166463.1	52157	51756	yes	yes
EDSPRL1	NW_015166463.1	35391	36422	yes	yes
EDPCS	NW_015173726.1	345212	344400	yes	yes
EDQK	NW_015173726.1	318908	319054	yes	yes
EDYA	NW_015173726.1	288732	289475	yes	no
EDSPR1	NW_015173726.1	267954	268367	yes	no
EDSPR2	NW_015173726.1	252541	252143	yes	no
EDSPR3	NW_015173726.1	239677	240120	yes	no
EDSPR4	NW_015173726.1	229052	228534	yes	no
EDSPR5	NW_015173726.1	210217	210564	yes	no
EDSPR6	NW_015173726.1	199660	199292	yes	no
EDSPR7	NW_015173726.1	185905	186366	yes	no
EDSPR8	NW_015173726.1	176343	175885	yes	no
EDSPR9	NW_015173726.1	166110	166583	yes	yes
EDCTT	NW_015173726.1	146998	148404	yes	yes
EDSQ	NW_015173726.1	28867	27827	yes	yes
EDEPT	NW_015173726.1	828	277	yes	yes
EDSPRL2	NW_015161985.1	65871	65695	yes	yes
CRNN	NW_015161985.1	132857	136952	yes	no
SCFN	NW_015161985.1	188866	195757	yes	no
S100A11	NW_015161985.1	215892	228376	yes	yes

Notes - * "RNA-seq evidence" corresponds to the presence of RNA-seq peaks in the "Genomic regions, transcripts and products" view at the NCBI GenBank website (www.ncbi.nlm.nih.gov, accessed on 27 March 2020). For genes located on some scaffolds, this view was not available (n.a.). The symbols < and > indicate that ends of the coding sequence were not present on the scaffold. Only the S100A genes flanking PGLYRP3 and SCFN are shown here. CBP/beta-keratin genes were reported by Liu et al. 2015. CDS, coding sequence.

Supplementary Table S5. Chinese alligator (*Alligator sinensis*) CBP genes

Gene	Accession nr.	CDS start	CDS end	Sequence complete
CBP1	NW_005843776.1	14927	15322	yes
CBP2	NW_005843776.1	38892	39287	yes
CBP3	NW_005844928.1	970	1539	yes
CBP4	NW_005843293.1	6303	6698	yes
CBP5	NW_005843293.1	20473	20928	yes
CBP6	NW_005843293.1	38862	39252	no
CBP7	NW_005843293.1	50698	51093	yes
CBP8	NW_005843293.1	57220	56816	yes
CBP9	NW_005843293.1	81993	81634	yes
CBP10	NW_005843293.1	98267	97782	yes
CBP11	NW_005843293.1	106917	107294	yes
CBP12	NW_005843293.1	128505	127948	yes
CBP13	NW_005843293.1	134659	135228	yes
CBP14	NW_005843293.1	141781	141224	yes
CBP15	NW_005843293.1	159177	158686	yes
CBP16	NW_005843293.1	164860	165426	yes
CBP17	NW_005843293.1	>195188	194952	no
CBP18	NW_005843293.1	203180	202836	yes
CBP19	NW_005843827.1	9986	<9816	no
CBP20	NW_005843827.1	21239	<20973	no
CBP21	NW_005843827.1	27575	27246	yes
CBP22	NW_005843827.1	35668	35213	yes
CBP23	NW_005844022.1	479	141	yes
CBP24	NW_005844022.1	5518	5856	yes
CBP25	NW_005844022.1	9390	9052	yes
CBP26	NW_005844022.1	17261	16923	yes
CBP27	NW_005842911.1	582806	582468	yes
CBP28	NW_005842911.1	575088	575426	yes
CBP29	NW_005842911.1	571820	572158	yes
CBP30	NW_005842911.1	567179	566859	yes
CBP31	NW_005842911.1	554888	555226	yes
CBP32	NW_005842911.1	550148	549810	yes
CBP33	NW_005842911.1	543674	544258	yes
CBP34	NW_005842911.1	506800	507279	yes
CBP35	NW_005842911.1	502103	501663	yes
CBP36	NW_005842911.1	496471	497001	yes
CBP37	NW_005842911.1	492176	491685	yes
CBP38	NW_005842911.1	479390	479022	yes
CBP39	NW_005842911.1	470088	470714	yes
CBP40	NW_005842911.1	457868	458509	yes
CBP41	NW_005842911.1	447145	448434	yes
CBP42	NW_005842911.1	430348	430953	yes

Notes - CDS, coding sequence; CBP, corneous beta protein

The symbols < and > indicate that ends of the coding sequence were not present on the scaffold.

A

>Sp_EDCC
MTCCGTSSGCGRGTVDVDFGPAARMNVCPGPCCLTICGPRLITHSETLRTCCSVVSGEECCRCCSGCSGAGGCTGATGPGVSVSLQGPTYEVISGGACLR
SSVHSASVQCSKTCDPCGPC

>Sp_EDETM
MSCFGGLGSYNPICSVSLPDSKIVIQPPLCVTNVALSEIDILDPEPRPYAVDECHLDKYGFPVVCCAPPEAPIICATFIYSIHQA

>Sp_EDGGY1
MTYGGYGKSWSYSSRSGRCNEPCGYGSGASGGYGHWYGSTTCRTRCSEPCGYGYGGYGGYGGYGGYGGNGCYGGYNRGCGSPRWSYPCSQGWGCKY

>Sp_EDGGY2
MTYSAGCCYPWGSSSCGSSCYDPCRSGCGYGYGRWGSRYSGSGCCEPCGYGGYGGGYGGYGNCCGYGGYGGYGGYGGYGGYGGRYGSRCSYPSWGCGWDS
CGSC

>Sp_EDGY1
MTHTKLKTCNQPITTHKYTSSSSCSQPCSSSSTYGYGTGCYRPRRYSCSYSHGCYQPYTCGTSYGSGSGYGCYQPCRSYGYGSGVSCYQPCTYGTSYGSG
SGYGCYQPCRSYGYGSGVRCYQPCTYGTSYGSGSGSGCYQPCQSYGYSSGVRCYQPCSYGSSYGSSCYEPCNYGYSSGVRCYQPRSYGNCGY

>Sp_EDGY2
MTYFRQKSYCDPCGSGYRYGSSSSSSCYRPCYGYGQSGGYGSFGSGSGVTCYQPSSYGYGCGISSCYQPCNYGYGYGQGVGCYKPYGMMSGSRLSYPFCR
RRYVYSNCGGPC

>Sp_EDGY3
MTYFRQKSYCDPCGSGYRYGSSSSSSCYRPCYGYGQSGGYGSFGYGSGVTCYQPSSYGYGCGISSCYQPCNYGYGYGQGVGCYKPYGMMSGSRLSYPFCR
RRYVYSNCGGPC

>Sp_EDGY4_partial
XXXGYGQGVGCYEPCGMISGSRLSYPFCRRRYVYSNCGGTC

>Sp_EDGY5
MNFYRQQSCYDPCWSDGWYGYGQRRWCYDPCNYGGWYGYGKRRWCYDPYSYRYGCYQPCYGYGYKSRCQYPWGSRRSSICGPC

>Sp_EDGY6
MNFYRQQSCYDPCWSGGWYGYGRGRRCYNPCYNPCNYGGGWGGYGQRRWCYDPCRYRRYGCYQPCYGYGYKSRCQYPWGSRRSSICGPC

>Sp_EDGY7_partial
XXXRRWSYDPCSYRDGCYQPWYGYKSRCQYPWGSRRSSICGPC

>Sp_EDPKC
MASNQNQHQCKQIPTLPPQLCKEPQIPEPVPCPEVKPCPEPIKPIPCCLQKPQTPVQIPVPCPEPVPCPKPVPCPEPVPCPKPVPTPCPPKQQQTPVQIP
APCPVPLPCPEPEKPTPCCQQKPQTPVQTPVPCPEPMPCPKPVPCPEPLPTPCPQKKQTPVQIPVPCPKPVPCPEPVKPIPCCQQKPQTPVQIPVPCPEP
VPCPKPVPCPEPVPAPCPQKKQTPVQIPVPCPEPVPCPEPVPAPCPQKKQTPVQIPVPCPKPVPCPEPVKPIPCCQQKPQTPVQIPVPCPKPVPCPEPMP
APCPPKKQECKEIPIPCPEPVQPTPPIQQQQCKQPIQWPPQQK

>Sp_EDPL
MSHHHQQSKQPNQSPPKCQELGPPPKIPVPCPPPPKCPELSPAFPECPEPCAIPPKGPPDHQSTINEIEYREPKKRNQTMSPPPVHDILPRFPTALGL

>Sp_EDPQ1
MYDSEYYRDQDSCQDYGRSVYSESCDGPPEGCHGRAVYSEPSCHNCESLSGYNSQGSCCYPLKARQQVKQQYHSPAQKCCPPAQKCHRLAQKKYCPLVQK
CAPPVQQQYCPPVQKCGPPVEQKYCPPVQKCRPLVKQQYCPPMQKCGPSVQQKYCPPVQKCRPLAKQQYCPPLQTCNPLPQQKHCSPAPKCCPPLQQKKC
YPPAQKQCCPPVQMYCPPVQKCPQKKQYCSPVQKCSPKQKYFLPHQQYCPPQQQYCPPLQQQQQCCSPVQKKQWCSEEQQNCPSGQQSCDPAAGPVLCQE
KQVCQVPAQLLKV

>Sp_EDPQL
MSSQKKEVQIASPQTCETPAKPTPTPEVPAETPPKPVQLQPTPPAPEQQQKKQCCPWPNPK

>Sp_EDQL_partial
MCSRRERGCXXX

>Sp_EDQM1
MGSSNTKEQQEQSGCHENRRSSCSGSTSSYQDSGSSSCGRSSGGCQSNHGSSCHSVGPSCQPQHNQQQQTCQVPSQKLN

>Sp_EDQM2
MSCQQNRGCQQSIPKQTGCSSGGCGRSGGGGCSRRGSSGGGCSSSGGSSSGGGCGSHGKQQCQQKQQCPIPQQKMK

>Sp_EDSPRL1
MSFPHQHQCKQPCLPPPICGQKCPAKCVEVCPPVCGDPCAPVCVRPCSPPCVPVSPPQCVDPCAPSCGDPCAPVCVRPCSPSCVPVCPPQCMDPCAPSCG
DPCAPVCVQPCQVKCVPVCPPQCVDPCAPVCVKPCADACAPKCVEPCTPACTNS

>Sp_EDSPR1
MSYQQQCKQPCLPPPIKCGPVCQPKCVPVCSPKCVPVCSPKCVPVCQPKCVPVCQPKCVPVCHPKCVPVCSPKCVPVCSPKCVPVCSPKCVPVCSPKCVP
VCPPKCPPSQNCCYQHKWY

>Sp_EDSPR2
MSYGQQCKQPCLPPPIKCVPVCPPKCVQPCPPPCVPVCPPKCIPVCPPKCVPVCPPKCIPVCPPKCVPVCPPKCPPSQNCCYQRKWY

>Sp_EDSPR3
MSYQQQCKQPCLPPPIKCVPVCPPPCVPVCPPKCVPVCPPKCVPVCPPKCVPVCPPKCIPVCPPKCVPVCPPKCVPVCPPKCIPVCPPKCVPVCPPKCVE
VCPPKCPPSQNCCQQKFY

>Sp_EDSPR4
MSYGQQCKQPCLPPPIKCVPVCPPKCVEPCPPPCVPVCPPKCVPVCPPKCVPVCPPPCVPECPPKCVPVCPPKCVPVCPPKCVPVCPPKCVPVCPPKCPP
SQNCCYQHKRY

>Sp_EDSPR5
MSYGQQCKQPCLPPPIKCVPVCPPKCVEPCPPPCVPVCPPKCVPVCPPKCVPVCPPKCVPVCPPKCVPVCPPPCVPECPPKCVPVCPPKCVPVCPPKCVE
PCPPPCVPVCPPKCVPVCPPKCVQPCPPPCVPVCPPKCVPVCPPKCVPVCPPKCPPSQNCCYQHKRY

>Sp_EDSPR6
MSYQQQCKQPCLPPPIKCVPVCPPKCVEPCPPPCVPVCPPKCVPVCPPKCVPVCPPKCVPVCPPKCVPVCPPKCVPECPPKCVPVCPPKCPPSQNRCQQK
FD

>Sp_EDSPR7
MSYQQQCKQPCLPPPIKCVPVCPPKCVEPCPPPCVPVCPPKCVPVCPPKCVPVCPPKCVPVCPPKCVPVCPPKCVPECPPKCVPVCPPKCPPSQNRCQQK
FD

>Sp_EDSPR8
MSYGQQCKQPCLPPPIKCVPVCPPKCVEPCPPPCVPVCPPKCVPVCPPKCVPVCPPKCVPVCPPKCVPVCPPKCVPVCPPKCIPVCPPKCVPVCPPKCVP
VCPPKCVEVCPPKCPPSQNRCQQKFD

>Sp_EDSPR9
MSYGQQCKQPCLPPPIKCVPVCPPKCVELCPPKCVPVCPPPCVPVCPPKSVPVCPPKCVDVCPPKCPPSPNCCQKKFY

>Sp_EDSQ_partial
XXXVEPCATVCKDACGSGCVTTCATKCVEPCTTTCIDSCGKTLPTICLDQCLLVKGRKC

>Sp_EDWM2_partial
MVVLSSGREPYYNLNSTWYDPAGSWLDNRRTPFRYVVNTSCVTGCNVRHDIPRRGGHNYRCYGYFGSSCCCGRSEPRFNCEGCSVMNPSIGCRDYWGRRL
GEGCHEGSRECHQCGRTISAGSRDGGCSAECHGRRRGVCSEPGCVSSGGGHGQGGVCSEPGCGSSGGGHGRGVCSEPGCGPSGGGHGRGVCSEPGCGPSG
GGHGRGVCSEPGCGPSGGGHGRGVCSEPGCGPSGGGHGRGVCSEPGCGPSGGGHGRGVCSEPGCGPSGGGHGRGVCSEPGCGPSGGGHGRGVCSEPGCGP
SGGGHGRGVCSEPGCGPSGGGHGRGVCSEPGCGPSGGGHGRGVCSEPGCGPSGGGHGRGVCSEPGCGPSGGGHGRGVCSEPGCGPSGGGHGRGVCSEPGC
GPSGGGHGRGVCSEPGCGPSGGGHGRGVCSEPGCGPSGGGHGRGVCSEPGCGPSGGGHGRGVCSEPGCGPSGGGHGRGVCSEPGCGPSGGGHGRGVCSEP
GCGPSGGRHGRGVCSEPGCGPSGGGHGRGVCSEPGCGPSGGXXX

>Sp_EDWM3
MAVCSSGRESYFNLNSTWYDPAGSWLDTRRKPFRYADNTCCVTCCNKKDDIPRRGGHNYRCYGYQGSSCCCGGNNSSKCYKKSCCPRRPSGGCRDYWGRP
LGDACNGSTGQSCSSGQSACDGCCRRHKSHRQGVCSEKCGGQSVCLEKSYVSSGGCSSRLSNKHC

>Sp_EDYM1
MAFYMHQCKIEHTYPASGVRFSPSPCVTVQHSTCITRNQQLCVPQPPEPCGVESQTPCYIRSANPCNVKCPTSCDIKCSESHDAGIRCLTPRAPKCAEKS
STPCTMPYPEPCGLKFSLQQSMPLYKQPCIARLPCGPILGGQCSIPSTSLCFMPRETYHYMTTRIYNSCYAK

>Sp_EDYM2
MAHYGQPCKQLGQLVPEYATKHLQQCAPRRIQPCAPKCYPSPCVPQYKKVACAPQYVQPCVAFCPRPCASKSRQPYVTTCPRLCAPKSPQPYVTTCPRPC
APKSPQPYVTTCPRPCATKSPQHYVTTCPQSCATKSPQPCVTTCPRPCTSKSPQSCAPKCLQPYPQPCVPKCPQSCITNYPQPYGPKCPRPYGLKSPRPF
VTSCPQLCASKCPKPVMTKYPRPCAPKYSQSCSQPCASGCLQPCFPNYTEGYTKNGKLSSHCRKSRSAPKWPF

>Sp_LOR1
MSQQRHSCGGGGGGHCGGGGGGGGGGCHGSGGGGGSSCHSGGGGGYQTQSSSCHSGGGGGGSHCQSGGGGCGGGGSSSCHGGSSGGYQMQVGGGGGGSSC
HGGGGGGGGYGGSSCHGGGVSSGVYQIPSSVGGGGSSCHGGGGGGGGSCQSGGGGGCGGGDSSCIDGGSICQQAKQGGQWPDQLK

>Sp_LOR2_partial
MSRQRQQSSRSGGCHSSGGHCGRSHSSGGGSSCHGGSSDGCSSSDYQIRYGSDGSGDGGGSSCSGSSHQIIITSGSGGHDCGGPPGGCHSSGGGHSGGQA
IIQSGGGSGDGCISDSSGGGSSGGYCFGGGSSSGGGGYGYSGLYQQKQKIVIPGGGCCIGGGSGGGGGGGGGGGTSGGNDSCGQIKIIGGGSSGCGSSGG
GYQEGASSGFDGGSGGVKIIGGGGGGGGGGSGGSGGVKIIGGGGXXX

B

>Sp_CBP1
MSCPAPVCCTTPCVTATPDATITIHPPRIVIKIPGMTLSTSPEDTVVETENPCATNCPQLFGTGSSTALVGSCPGPRAVCGTTPCITTTTPDSQVVIQPP
PVCLTIPGAVLTTYPQECIVQTSGPCAIVGSGSRAITRSTSVGGDCTSVRALSRSSSVPCSTEACVTKAPDSRVVIYPPPIEITIPCPVLTISPEECLVE
IESPCTSKCPQLCGVQAMVASPDVCAEVGACVSQGPETKLMIQPPPIVVTVPGPILSVFPEECKVVTETPCAIDDPPPCSKTTSTALATTSTSLCAVRRP
LPNVRRPPRQWADRYSGAYLPRGYCSYTYPSRVSQSSASSGIYRSR

>Sp_CBP2
MSGRQECYASCPPTTMTIQPPPFVLSVPGPAIYCPSEDLCIEQYNPCASSGASSASLLPSSYPPPSEERYVSTLANLHSSRLARSGIYKNYRPY

>Sp_CBP3
MESRRLDPKCYACCPPSTVTIRPPPFVLNIPGPALYCPSQPFGIEQHNPCATRSGGGAFVDAFTYEDESGSPLSNFYDREALPSSTFGYRSRYWN

>Sp_CBP4
MESRRPDPRCYACCPPSTVTIRPPPFVLNIPGPALYCPSQPFGIEQHNPCATRYGGGALVDAFTYDDESGSSLSNFYDREALPSSTFGYRSRYWN

>Sp_CBP5
MESRRPDPRCYACCPPSTVTIRPPPFVLNIPGPALYCPSQPFGIEQHNPCATRYGGGALVDAFTYDDESGSSLSNFYDREALPSSTFGYRSRYWN

>Sp_CBP6
MSSGGKQECYASCPASTVTIQPPPFVLTIPGPSLCCPDKPFGIEQHNPCAVSGGHSGGGNHEAISHGDSSGSHIPSFYTRRALPSSLYSYRTNYQFY

>Sp_CBP7
MASSTNPNCYAQCPPSTVTIQPPPFVLTIPGPALYCPNQPFGIEQHNPCAGITPRGGGMEAIAYNDDFGSNFSSFYDRSALPGNTYGNRSFYRSY

>Sp_CBP8_partial
XXXPPPFVLTIPGPALYCPSQPFGIEQHNPCAGITPRGGAMEAIAYNDDFGSNFSSFYDRSALPGNTYGNRSFYRSY

>Sp_CBP9
MSSATNPNCYAQCPPSTVTIQPPPFVLTIPGPALYCPNQPFGIEQHNPCAGITPRGGGMEAIAYNDDFGSNFSSFYDRSALPGNTYGNRSFYRSY

>Sp_CBP10
MSWREQCEFNCPASTVCIQPPCFTVTIPGPSICCPNQPLSIQQWNPCTRYDWSRPTLRSDNSSGNSFYDRGLGSNLTSFYD

>Sp_CBP11
MSWREQCEFNCPASTVCIQPPCFTVTIPGPSICCPNQPLSIQQWNPCTRYDWSRPTLRSDNSSGNSFYDRGLGSNLTSFYD

>Sp_CBP12
MSSSWNPCQATCPPSTVTIQPPPFILTIPGPALYCPDQPLCIDQYNPCAYGYGALTDGRYTSLSSGRAFGSSVTSPYARQTQSRSVCNYGSCRPY

>Sp_CBP13
MSSPVNPCYAQCPPSTVTIQPPPFVLTIPGPSICCPYQPFGIVQNNPCDYSYGGVSGGRAEALSYSGSSISNLYSRRALPRSTGNYGSCWPY

>Sp_CBP14
MSSSWNLCQATCPPSTVTIQPPPFVLTIPGPALYCTDQPLCIDQYNPCAYGYGGLTDGRYTALSSGRAFGSSVISPYARQTQSRGSCSSGSCRPY

>Sp_CBP15
MSSSWNPCQATCPPSTVTIQPPPFILTIPGPALYCPDQPLCIDQYNPCAYGYGALTDGRYTSLSSGRAFGSSVTSPYALQTQSRGSCSSGSCRPY

>Sp_CBP16
MSSSWNPCYATCPPSTVTIQPPPFVLTIPGPSICCPDQSFCIEQYNPCAYGYGGSSISNLYSQRALPRSTGNYGSCWPYQTMKK

>Sp_CBP17
MSSSWNPCYATCPPSTVTIQPPPFVLTIPGPSICCPDQSFCIEQYNPCAYGYGGSSISNLYSQRALPRSTGNYGSCWPYQTMKK

>Sp_CBP18
MSSSWNPCYATCPPSTVTIQPPPFVLTIPGPALYCPDQPLCIDQYNPCAYGYGGLTDRRYTALSSGRAFGLSVTSPYARQTQSKGTCSPDSCRPY

>Sp_CBP18L
MSSSWNPCYATWPPSAVTIQPPPFVLTIPGPALYCPDQPLCIDKYNPCVYGYGRLTDGRYTALSSGRVFGSSVTSPYACQTQSRGSCSPGICQPY

>Sp_CBP19
MTGCGYIPSCPAPVADNCAVPLNNLAPLCGDGYGYGYSGLGLGLGLGGGFAGGVSALAGPLPGVNTSCVSQIPASEVVIQPSSFTVIIPGPILSAGAEPV
AVGGNTPCAVGSYGGGYGSLGYGLGLGYGSRLGYGYGLGYGCGLGYGYGRYAQRRLSNIC

>Sp_CBP20
MALCNYNCNTGCGIPISCVNQVPASDMTVKPPVCTVTIPGPILSSSSEPLLLNGTSPAGISHLGYGGYGYGGYGGHGGFGGYGGYGGYGGYGGYGHGCGR
FGYSSCY

>Sp_CBP21
MALCNYSCNTGCGIPTSCFNQVPASEMAVKPPVFTVTIPGPILSSSSEPLLLNGTSPAGISHLGYGGYGYGGYGGHGGFGGYGGYGGYGGYGGYGGGCGR
FGYSSCY

>Sp_CBP22
MTGCGYIPSCPAPLAANCAVPLNNLAPLCGDGYGYGYGGLGLGLGLGGRFAGGVSALAGPLPGVNTSCVSQIPASEVVIQPPAVTVIIPGPILSASAEPV
AVGGNTPCAVGSYGGGYGGLGYGLGLGYGSRLGYGCGLGYGYGLGYGCGLGYSYGRYGRRCLSNIC

>Sp_CBP23
MSSCGYIPSCPAPFCGVPCGPRPLNNLAPLCGDLSGCGSSCGPVNTSCVSQIPATQMTVQPPCFCITIPGPILSASCDPVAVGGNTPCARGSYGSSGYGV
CRPC

>Sp_CBP24
MSSCGYIPSCPAPSCGVPCGPRPLNNLAPLCGDFSGCGSVCGPVNTSCVSQIPATQMTLQPPCFCITIPGPILCASCDPVAVGGNTPCARGSYGSSGYGV
CRPC

>Sp_CBP25
MSSCGYIPSCPAPSCGVPCGPRPLNNLAPLCGDLSGCGSICGPVNTSCVSQIPATQMTVQPPCFCITIPGPIMSASCDPVAVGGNTPCARGSYGSSGYGV
CRPC

>Sp_CBP26
MSSCGYIPSCPAPSCVVPCGPRPLNNLAPLCGDLSGCGSICGPVNTSCVSQIPATQMTVQPPCFCITIPGPILSASCDPVAVGGNTPCARGSYGSSGYGG
SYGSSGYGVCRPC

>Sp_CBP27
MVFSGCIPSCPPLVPLNNLAPLCGGLPAYGGCGYGLGCGIGYGYLSSGLVGPLSGANTSCVSQIPPSEVVIQPPPSVVTIPGPILSASCEPVAVGGNTPC
AVGSYGSGYGLSGLGHGYGLGSGYGLGYGLGCVGYGPGAIARTYGRRGLLSNCGFF

>Sp_CBP28
MSRCGYPQSCVPSCCVPPVCSTGSSGGGICYGKGGGIGYGMGGGMGGVISYGGLSSGSIGVDTSCVSQIPAAEVVIQPPAFTLCIPGPVICASSEPVGVG
CYTPCAFPPYGCGPLGMGGYSGQGGYSGQGGYSGQGGYSGQGGYSGGGRRRRYSSSGKCGPCA

>Sp_CBP29
MTYCGYPQPCVYPTVSSSCGVPLNNLAPLCGTGSAYGGSGYGIGGGIDYGTLSSSPAGVNTSCVSQIPASEVVIQPPAFKLCIPGPVMSASCEPVAVGGN
TPCAVGSYGGGSLGLGGYSGLGGYSGLGGYSGLGGYSGMGGYCGVGRRRRYLSSGYCGPCA

>Sp_CBP30
MASCIYVPACVSPTVSSSGGVPLNKPTSSTESANGGFGYGISGGIGNSGLSSGLAGGNTSCASQIPASEVVIQPPAFTLCIPGPVIRASCEPVAVGCCTP
CAVPPYGCGPLRRGGYCGVGRRRRHLSSGYCGPCP

>Sp_CBP31_partial
XXXYGGYGCGIPTSCDSQIPASEVNIQPPCFNVTVPGPILSASCEPLGVAPCTPCAGWGGSGGWGRGSGGWGRGSGGCGYGGWGRGYGGCGYGGWGRGYG
GWGGNCCY

>Sp_CBP32
MTFGGYGGCGYGGYGRYGGCGYSGYGGYGGYGGYGCGIPTSCDSQIPASEVKIQPPCFNVTVPGPILSASCEPLGVAPCTPCAGWGGYGGWGRGSGGCGY
GGWGRGSGGCGYGGWGRGSGGCDYGGWGRGSGGWGGNCCY

>Sp_CBP33
MTFGCYGGGGYGGCGYGGYGGYGGCGYGGYGGYGGHGGYGGYGGCGYGGYGGYGGYGGYGGYGGYGGYGGYGGYGGYGGYGGYGGYGGYGGYGHGLPTSC
VNQIPASEVTIQPAPFNVTVPGPILSASCEPLGVAPCTPCAGWGGYGGWGRGYGGRGYGGWGRGYGGCGSGGWGGNCCY

>Sp_CBP34
MTFGCYGGCGYGGYGGGGYGGYDGYGGCGYGGYGGYGGYGGYGGYGGYGGYGGYGGYGHGVPTSCVNQIPASEVTIQPAPFNVTVPGPVLSASCEPLGVA
PCTPCAGSGGYGGWGRGYGGRGYGGWGRGSGGWGGNCCY

>Sp_CBP35
MTLGGYGGYGYGGYGCNTGCGIPTSCVNQVPASDVMIQPPCFCVTVPGPILSASCEPLAVAPCTPCATNCCSWGGYGYGNRGWGGYGVCGRGYGVWGY

>Sp_CBP36
MTFGGYGGCGYGYGGCGYGYGGYGRFGGYGGCGIPTSCISQVPASEVVIQPPCFCVTVPGPILSASCEPLGVAPCTPCATNCCGWGGYGYGNGGWGGYGG
WGRGWGCGYGGGWGY

>Sp_CBP37
MALANYNCNTACSIPTSCVSQVPASEVQIQPPCFCVTVPGPILSASCEPLAVALCTPCATSYPGWGGYGYGNRGLGYGGSGCWGYRSRGFALGSRYGY

C

>Sp_Crnn
MPQLLSNISGIIGAFYKYARNNGSCTTLSKGELKGLIQQEFADVIENPYNVETIDKVMQLLDTDCDGRVDFNEFLVLVFRVVRACYKECQEAAKGQFQRE
ADAEQNPGEPKIPEQGASRQQTREPLTPEQDKSHQQTQEPQTPEQDQSRRQTQEPQTPEQDESRRQTQEPQTPEQDESRPQTQEPQTPEQDESRLQTQEP
QTPEQDESRPQTQEPQTPEQDESRLQTQEPQTPEQDESRPQTQEPQTPEQDESRRQTQEPQTPEQDQSRRQTQEPLTPEQDKSRRQTQEPLIPEQDESRR
QTQEPLTPEQDESRPQTQEPQTPEQDESRRQTQEPQTPEQDKSHRQTQEPQTPEQDKSHQQTQEPQTPEQDKSHQQTQQPLTLEPDKSHRQTQEPQKPEQ
DPSHQQSQEPQTPEQGQNDQQTPEQDQTRRQTQEPQKVEQDPPSRRQSQEPLTPEPDQTRRQSQELQTPERGQSRGETPSPPIQGLVPVPPGQREPPSGQ
ACIITPRPYSQVHQKPIKFPYYWPPKP

>Sp_Scfn_partial
MPHLLDSICTIIALFYKYARKDGDCSTLSRREMKRLLTKEFAGVIEDPCDPQEMDAVLQLLDRNGNGLVDFNEFLVLVFKVAKACYSYLLPRECLLPRRE
ATRHHEPESQTDERIRHQQHGTETTEDHPRRDRQPWKDEEIETGPQEPELYKSERHQLEPSVNKESHRQPLEPEQIGDHGRGPSREPQQKEDKESQSQPR
KSEPQVDERSRQQPQKPEPQEDEWRHQSPSLSEQQVDEGRRHHPHQPEQIDSYGRRHPSHEPQTWDEEESQHHLLESELRGDERRHHQPKEPEPQVDEGH
HYQTREPESDVDEGRHRQSRAINPIDDYGRYSQSHERQSWEDKDRHHQPREPEPQWDVERQHQPYAFDPTEDYWKCPPSCEPQRWEDKDRKHQPKQPEPR
VDVNHHHQPHENVDYHHQPQKREPQRDGERQHQPRATDPRDDYRKHYSLHEPQIWEDEDKKHQPRDPKPQSDVDRHHQTQEPEPQADVARQVWEHEAQAD
VDHRRQLQEPEPQTNVDRHRQLREPEPQAVVDHRRQLREHEPQAAVDRRRQLREHEPQAAVDRRXXXQLREHEPQAAVDRRRQLREHEPQAAVDRRRQLR
NPEPQADVDHRHQLREPEPQAAIERRRQLQEPEPQAYVNRHRQLREPEPQADVNRHRQLREPEPQADVDHRRHLREPEPQADVERRRQLREPEPQADVNR
HRQLREPEPQADVDRHRQLREPEPQADVDHRRQLREPDPQVDVDRHRQLREPEPQAAVDRHRQLREPEPQVDVESQVDVDHHQLWEPRPQADVDHRRQSR
EPKPQVNVDHHHQTQKPELQEDVDHHRQPWESELQEDVDHYHQLQRHEPQIDVDRSHHQLREHEPQQDGERQHQQHTIDPIDDNWKHSSLRELQTWEDED
KQHQPRKLVPHVDVDHHYQSEDPAPQVEVKLRRQPWDSEQQAYVDRQPRAPETQVDLYRHHQPWDLEPQWVLNSHRQSWENEKQGFVGSHHQPHEREPQW
NEERQHQPRVIDPIYDDWRSSPLREPQTWEAEDRHHQPREPESHGDVNRRHQPREAEPPVDMHRHPWENKSQRDVDHFRQPQEPKTRVDINPCHWSREPE
PQWDVNHRRQTWEPELQVDTERRHQPDEHEPQWDGKRRHQPQAVDPIDNYWRCSSSHEPQTWGNNDRQHQPRDPGSRKDVDRQSLPRELEPRVNVDHRHQ
SREPEPRVDVDHRHQPREPEPRVDVDRRHQSREPELRVNVGLRRQPRKPEPQVDVHHHHKPREPELQVDVDHHRQPREPEQRMDMGRHRQPREPEPRVDV
NHRHKPREPELQVNVDRHRQPREPEQRMDMGRHRQPREPEPRLDVNHHHKPRESEVQVDVDRHHQPREPEPQVDVDHHHQPQKPKPRYVERRCLPRDIDS
VDDYLRCSPLYELQKWEDEGRQHHRRKPKAQVKEDSRHQSRDLGPQVDADSHRQPQEPEPRVEEHNHSQHRETIPRREVRNHWPQEPKLQADVGRHPQER
ETQSQLEEGECHQTWESEPQVNESSHHQPCDTEPKGDYWRCSPSRTWIEPRENGQRRPQLIEGERRMPQIHTYETTEGERARPKSRELHTREREQRRPLP
HEVGTKEGDRRRPQTHDPESREGEQRRPHQHELYPIECEWERPRSCQLEPNKREEKKTKQYDPESTKGEERNTERHDPEPTKREERNTERRDPEPTKREE
RNTERRDPQPTKREDRNTERHDPEPTKREXXXPTKREERKTERREPEPTKREERKSERREPEPTKREERKTERREPEPTKREERMTKESEPEPTKREERN
TERHDPEPMKRDERKPEQYEPDSTKREERKTERREPEPMKRDERKPEQYEPDSTKREKRETEQREPESTNYEGRRSQPRRPEPIDSHQGLNKTPKSQPQK
DDRREKRPHESEQGNGESRHWRLKESELQGDESRKGEQRRPQVKGPERIEDMGIQSLPREPYGVESDRTQSYSQACEPQPPLEKCSVGEEPQRGECSQHN
LDPHDPNTSREEQGKGYLCEAKVVLPCNPLYEYLLAQKVKKQ

D

>Sp_EDKM
MSKLIKAIADMIDSYQGNSKKGTEAEKFKRSEFKKLIHQELSPVKISSSSKYKHIKNLPDSDMELMNEKEIQPCVY

>Sp_PGLYRP3
MSKLSAFLSFLCALSQKTEHLPLIAPAKWGAVPAKCSGPLKKVPPEYVVIIHTAGSYCHTQTECSRILRDIQALHMNDNNWCNIAYNFLIGEDGNVYQAV
GWLGEGEHTFGYNDLSLGIAFIGDFTKREPNAAAWNALKNLLSFAVKNGYLASNYLLLAHGDVSNTLSPGKYIHDVIKKWPHYKH

>Sp_S100A11_partial
XXXSSKYTAGPTETERCIESLLAVFQRYAGRDGDSATLSKREFLTFMNTELASFSKNQKDPAVLDRMMKQLDTNCDGQLDFAEFLNLIGGMAQACHVSLA
VQGPPKPQQNPKKP

>Sp_S100A12_partial
XXXTQMESSFEIIINMFHAYSVRQSHPDKLNKGELKQLIEKELPNLILEATDKSTMDKIFKDLDKNKDNEVDFEEFMALITKVLITCHNKIHGHGHGHGH
GHSH

Supplementary Figure S1. Amino acid sequences of proteins encoded by EDC genes of the tuatara. **(A)** Amino acid sequences of proteins encoded by tuatara SEDC genes excluding corneous beta proteins (CBPs). **(B)** Amino acid sequences of CBPs, also known as beta-keratins. **(C)** Amino acid sequences of SFTPs. **(D)** Amino acid sequences of proteins encoded by other EDC genes of the tuatara. The following amino acid residues are highlighted to indicate residues important for protein cross-linking and the peculiar amino acid compositions of SEDCs and SFTPs and: lysine (K) and glutamine (Q) are potential sites of transglutamination; cysteine residues (C) are potential sites of disulfide bonds; glycine (G), proline (P) and serine (S) are highly abundant residues not directly involved in cross-linking. Stretches of unknown amino acid sequence due to gaps in the corresponding gene sequences are indicated by “XXX”. Only the S100A proteins whose genes flank *PGLYRP3* and *Scfn* are shown here. SEDC, Simple epidermal differentiation complex gene; SFTP, S100 filaggrin-type protein; Sp, *Sphenodon punctatus*.

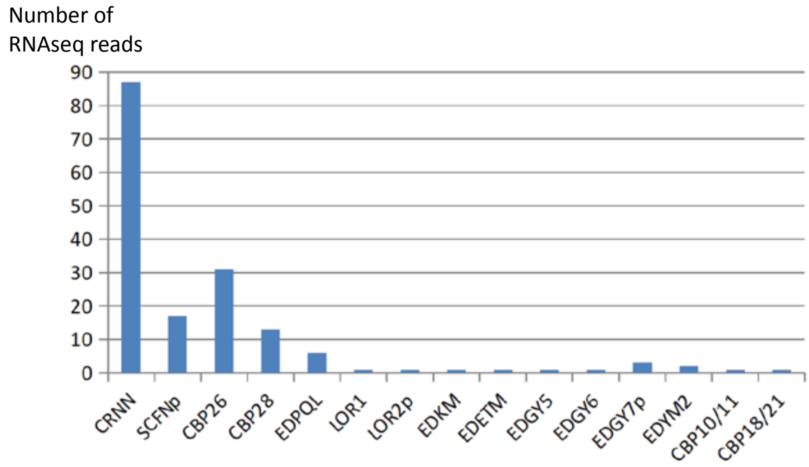

Supplementary Figure S2. Expression of EDC genes of the tuatara during embryonic development. The diagram shows the number of sequence reads corresponding to EDC gene transcripts in the sequence read archive (SRA) of tuatara embryos (GenBank accession number SRX141978) (Miller et al. BMC Genomics 2012;13:439). The transcriptome was obtained from head, trunk and tail of an early-stage embryo. Amino acid sequences of EDC proteins were used as queries in tBLASTn searches. For some proteins (marked by "p" after the name) only partial sequences were available as queries.

A

```
>Gj_EDCC1
MACCGTSCGTGCGGGGGGSSGSGGVLVDSGPANVVSVSTGGSSLTIPGPRLQVHNNVRVSCQCVCASECCMPCGGCCGGHGENGPGARVDISHPCYEVIT
GGGYLCSNVHCARASTSCCCDPCCGPC

>Gj_EDCC2
MACCGTSCGTGCGGGSGGSSGSGGVLVDSGPSNVVSVSTGGCSLTIPGPRLIVHNNVRVSCQCVCASECCMPCGGCCGGHGENGPGARVDICHPSYEVIM
GGGYLCSNVHCARASTSCCCDPCCGPC

>Gj_EDCC3
MACCGTSCGTGCGGGSGSGSGSGGVLVDSGPANVVSVSTGGSSLTIPGPRLQVHNNVRVSCQCVCASECCMPCGGCCGGHGENGPGARVDICHPCYDVIT
GGGYLCSNVHCARASTSCCCDPCCGPC

>Gj_EDCC4
MACCGTSCGTGCGGGSGSGSGSGGVLVDSGPANVVSVATGGSSLTIPGPRLQVHNNVRVSCQCVCASECCMPCGGCCGGHGENGPGARVDICHPCYDVIT
GGGYLCSNVHCARASTSCCCDPCCGPC

>Gj_EDCC5
MACCTSSWASRGSGLVSSRNDEVLVDYGPENIVRVHPGHCSLTLPPARVSSSNQPFRISCFVVGSSKCKT

>Gj_EDCC6
MACCKTSCGTGCGTGCGTGCGTRWGSGYGTGCGIGCGTGCGTGCGTGCGTGCGIGCGTGCGTGCGTGYAGGHEGSSLFVDNGCTSVVCVKPGDSHLTIPG
PRLIPHCDLQVCCHSVCVSECCMPCGCRPCGGCHGENGPGVEVDVRHPSYEVVIPTGYLYSCAHCAKVDTTCCDPCCD

>Gj_EDCC7_partial
XXXGYLCSNVHCARASTSCCCDPCCGPC

>Gj_EDCC8_partial
XXXDVIAGGGYLCSNVHCARASTSCCCDPCCGPC

>Gj_EDCG
MGCCGCCGGDRSRVVYCVMPCCYPSMQSCCCSCCGPQQHTQACCAPMQTKCCSQTKKCC

>Gj_EDCM1
MSCCRRCGGRGCSCCCCRRARPRVVYYVGRPCVPYGGYPGYTYVPSYIPARSCCCSARPCCSGSQGFSQVKKC

>Gj_EDCM2
MSCCRRCGGRGCSCCCCRRARPRVVYYIGRPCVPYGGYPGYTYVPSYIPARSCCCSARPCCSGSQGFSQVKKC

>Gj_EDCM3
MSCCRRCGGRGCSCCCCRRARPQVVYYVGRPCVPCGGYQGYTYVPSYIPARSCCAPARPCCYGSQGFSQVKKC

>Gj_EDCM4
MVFCRRCGGRGCSFCRGQRDKPKVIYYIGAPAAPCCGCRQYTYVPCYVPAPSSCAPACPCSHAEQGCIQVKKKH

>Gj_EDCM5
MSCCRCCGGRGCGCCCGRRAGPRVVYYVGRPAAPRCGYNQYTYVPSYAPVQSCCAPAYYGSSGQQCCNQVKKC

>Gj_EDCQ3
MGSCGRDSCCGEERPKVIYCVMPCYGARSWCTPYCTQIQSSCCSCCTPRKFC

>Gj_EDCRP_partial
MACKKAFGFMQCKKSSCAPCCFKKGCCSKCGCCPCCCNKKSCCCKGDCNTSCCKPQSCCCSKCGCNPCCCKPQSCCCSKCGCNPCCCNQKSCCCKPQSCC
CSKCGCNPCCCNQKSCCCKPQSCCCSKCGCNPCCCNQKSCCCKPQSCCCSKCGCNPCCCNQKSCCCKPQSCCCSKCGCNPCCCNQKSCCCKPQSCCCSKC
GCNPCCCNQKSCCCKPQSCCCSKCGCNPCCCNQKSCCCKPQSCCCSKCGCNPCCCNQKSCCCKPQSCCCSKCGCNPCCCNQKSCCCKPQSCCCSKCGCNP
CCCNQKSCCCKPQSCCCSKCGCNPCCCNQKSCCCKPQSCCCSKCGCNPCCCNQKSCCCKPQSCCCSKCGCNPCCCNQKSCCCKPQSCCCSKCGCNPCCCN
QKSCCCKPQSCCCSKCGCNPCCCKPQSCCCSKCGCNPCCCNQKSSCCKPQSCCCSKCGCNPCCCKPQSCCCSKCGCNPCCCNQKSCCCSKCGCNPCCCKP
QSCCCSKCGCNPCCCKPQSCCCSKCGCNPCCCKPQSCCCSKCGCNPCCCKPQSCCCSKCGCNPCCCNQKSCCCSKCGCNPCCCNQKSCCCSKCGCNPXXX
PQSCCCSKCGCNPCCCKPQSCCCSKCGCNPCCCKPQSCCCSKCGCNPCCCKPQSCCCSKCGCNPCCCKPQSCCCSKCGCNPCCCKPQSCCCSKCGCNPCC
CKPQSCCCSKCGCNPCCCSRKAKYCSKCGCSPCCCKPQSCCCSKCGCSPCCCQKKSCCSICGCNPCCCTQQRSCKKCGCSPCCCPQPCCPKCGCSPCCCK
PTSCCSKCGCNPCCCKPQSCCCSKCGCKPCCCKPQSCCCSKCGCKPCCCKPQSCCCSKCGCSPCCCKPTGCCSKCGCNPCCCNQTPCSKCGCCPCCCAQR
GGIKVCSCMPSGLPCCTSPCLFQCTLPPCCPPPCCIPSCCPGAPCYQ

>Gj_EDCS1
MTCCSCCGGGGQIICIPVSQMYRMGSSCCGSGCGSCYCSGCGSGSGSCCGSGSSSGSCCGSGSGSCCGSGSGSGCGCGSSGCNSGSCCGSSGSSCRIIVL
RPTCCPPMGCGQYCYPPKKRQCCAQYCC

>Gj_EDCS2
MNCCGGGGGCRLILINSGNSGGGCDCDCDCGGGGSNNNGMRVICLPNSCCGGCCQPSCQPMVYCCGGNNRGGCCG

>Gj_EDCS3
MNCCGGGGGCRLILINNGGGGGGGCDCDCNCGGGNNNDGMRVICIPNSCCGGCCQPSCQPMVYCCGGNNRGGCCG
```

>Gj_EDCS4
MSGCGCGGGNDNNMMILCVPCGNEGGCCKPCCCCQPCCCCQPCCCCQPSCCCCQPSCCCQPSKGCCQPCCQKKCCTPCCPPCCQAKKC

>Gj_EDCS5
MEFPEDDAFDPTLDEFWCRRPCCCIRSCCCCRPSCYQPSCCCRPLCCRPCCCCRPCCCCRPSCYQPSCCCRPLCCRPSCCCRPVCCCRPSCYQPSCCCRP
LCCCQPCGCCCRPCCCCQPW

>Gj_EDCS6
MVSGCCSSCHGSTILVCPTSTCGGQSCCSPCCGQSCQSCCVPCCCIPVCCSPCCGQSSCCSPCCVQSSCCSPCCVQSAGCSPCCGQSACCTKSSGCSQSC
VPCCAPLCCTSETKCGTKKC

>Gj_EDCTT
MAYQCKQPCVGPPSFVKGSTISYVDADADKSSDVPPCIEPCQAAPMDASKTAAVGSQTAGTKASKAAVKAADTTEVCAKVAVGRSKVVCTEGCGNFPTKT
IKSSTPSQAQGPTCVPVCKTPDQGPACVPVCTSPSSAPAYVPICKTPDQGPAYVPVCTSPSKGPVCVPVCATPGQGPTYIPVCVAPGHAPAYVPICTTPG
HPPTYVPVCTSPGHPPTYVPVCATPGQAPTCVPVCTTPGQAPTCVPVCTTPGQAPTSVPVCATPGQAPTCVPVCTTPGQAPTSVPVCTTPGQGPTYVPVC
TTPGQGPTYVPVCTTPGQGPTCVPVCTTPGQGPTYVPVCTTPGQGSTCVPVCTTPGQAPTCVPVCTTPGQGPTCVPVCTTPGKAPTCIPVYATPGHAPTC
VPVCKSPSRVPYSGMSCKSPPSSPVWYVQCCRSGCGGGCGYMFLVTHPAQGVTPYTCCSRSPSSKSQG

>Gj_EDEPK
MSGAYQQRKQNPVLPRALTKSTPESVSEAEPPEPDHESKRHPPSRKEPEKRPQEENGSNAKEEPPNQAPTSGAEAKEVPEPEPEAPVEKEKEHPHQDEHL
DGHPPQAEQQEKESATLPPAPSKEAINPDHPPAKEAGGLLPSQPPPPVEEIQQQKKQAAQWPPK

>Gj_EDEPT
MAYQCTQPSLPPPIHGQQGSSGAQEPSTCASSCTTASADCGSPAYQMTQACLPPPIFMQPRPTECANAPAPPFVPMCHAVIADPGPTARPEVSSSSGGPC
TEQPCLPLVEVVRPATAGIVDPNMARYSHANAEASPAPCPEATPAYVTPGSVVSLHPSGRITVRPGPVGEASVSMGASMPHIS

>Gj_EDETM
MSFPGELCGLNTTYSTSLPASTLLIQPPTYVTETILTDLEILIPEPPPYEYDECHPGHLLPYYCHLPPEPPLVFATFTTELFEV

>Gj_EDGGY1
MSYGNCCGYSCCSPCGYGGWGYGGWGGYGGCGGCCGGYGGYGGHGCCGNRCSYSSCCSPCCGGWGWGW

>Gj_EDGGY2
MSYGNCCGSSCCSPCGYGGWGRRGWGGYGGWGGYGGCGGCCGGYGGYGGYGGYGGYGGYGGYGGRCGNRCGYSSCCSPCGGGWGWGW

>Gj_EDGGY3
MATYGMHSMSHSSSQPSHGVSGQGFGAGHWYGSSTSHPRCYEPTSYINLGGPSAGGGYGSCGGPSSYGMLSCRNTSGGPGTYGGGYGGPGGYGSPGGPGG
SVSWCSPRSYSSPQWGSSWGN

>Gj_EDGY1
MSLYRHQSYWNPCRRGSFSSGSCYGGSGQRISYYPSYGRSCYPQSCYGGSGQRISYYPSYGRSCYPQSCYGGSSQRICYYPQTSYGGYGRSYYPQSSWGG
YGYGYDRGCYPQQYYGGYGYGRRYGSGYGSRRGSMCWGYGSRRGSICYEPCYPIKRRYSCSSIDGPC

>Gj_EDGY2
MSLYRHQSYWNPCRRGSFSSGSCYGGSGQRISYYPSYGRSCYPQSCYGGSSQRISYYPSYGRSCYPQSCYGGSSQRICYYPQTSYGGYGRSYYPQSSWGG
YGYGYGRGCYPQQYYGGYGYGRRYGSGYGSRRGSMCWGYGSRRGSICYEPCYPIKRRYSCSSIDGPC

>Gj_EDGY3
MSLYRHQSYWDPCRRGSFSSGSYYGGSGQRICYYPQTSYSGYGRSYYPQTYWGGYGRSYMPPLYCGGYGYGRGYYPQQNYGGYGYGGLYGRRYGWGYGSR
RGSMSWGYGSRRGSICYEPYGYSSGGCYPLKRRYSCSSIDGPC

>Gj_EDGPC
MGNSGSSGGGGCGCDCCGGGGGSDIICCEVPGGGCCQGCCCQPCCGMKCCPQSCCGCQGCGCGCGCPKSC

>Gj_EDMTM1
MCDCCGGSSDMTVLCLPAGGQGCCGCQCGCGCQCGCSCQPSKCKSCCQDSCSCSCCQEKCCTPCCQCKKC

>Gj_EDMTM2
MCDCCGGGSSNMTVLCLPCGNQGGGQSCCCKPCCQPCCQPCCQPCCCKPCCQQSCSTPCCPPCCQSKKSC

>Gj_EDMTM3
MCDCCGGGSSNMTVLCLPCGNQGGGQSCCCKPCCQPCCQPCCQPCCCKPCCQQSCSTPCCPPCCQSKKSC

>Gj_EDMTM4
MCDCCGGGSNNMTILCLPCGNQGGGQSSCCQPCCQPCCCYKPCCQQSCCAPCCPSRRCC

>Gj_EDMTM5
MSGCGCGGGNGGNMTVLCLPAGNQGGGCCQPCCCCRPCQSCCQTCCCPCRRCC

>Gj_EDMTM6
MSGCCGGGGGGDMTVLCVPCGNEGGCCQPCCCCQPCCCCKPCCCQSCCQQCCQPSCCQCKKSC

>Gj_EDMTM7
MGSMFSCGRGGGGDNMTVLCVPSENEGGCCQPCCCCQPCCCCQPCCDQSCCQPCCCQCKKS

>Gj_EDMTM8
MSGCCGGGGGDNMTVLCVPCGNEGGCCQPCCCCQPCCCCKPCCCQSCCQQCCQPSCCQCKKSC

>Gj_EDMTM9
MSGCCGGGGGDNMTVLCVPCGNEGGCCQPCCCCQPCCCCKPCCCQSCCQQCCQPSCCQCKKSC

>Gj_EDP3
MSYQQTQQCKQPAVCPPAPCVTPCQPSECPKPCPPPACQVSGGSTCQVSGGSTGQVSGGSTCHGSGGSPCQSADPCQGTTGSQSQCPSGKGDSHQNHHDS
QQHKQC

>Gj_EDPL1
MSSQQTHYPTNQPSCTGSSTTGPPKKTSDQGSPSCGSPQSQQKKQPPPQCPQAPK

>Gj_EDPL2
MSHHHQQKKQPNLPPHKGLQTGPSPEVSETSSVPPKSPEPSRSPPKSSEPSPAALPSGPPHSHQASGPKHC

>Gj_EDPCS
MYFHQLNPELVDVSHLHFPESGLNVLIRQSPDCLPSHDSSLCTRPCGVKCPSKYDPCQRVCRPSVPVCRPRYMDPCRPYCVRPRCVSPCRPYCPSPCAPQ
CMSPCRPYCPSPCAPQCVVPCRPYCPSPCAPQCMSPCRPYCPSPCAPQCVIPSRPYCPSPCAPQCVIPYRPYCPSPCRPSCLSPCRPYCVSPCRPQCLSP
CRPYCVSPCAPQCMLPRPQCVYPRPLCAQPCRPYCPIPTSPICVNPCSPCSPCDYLDLEYVDLDDYYDEY

>Gj_EDPKC
MASSSNQHQSKQAASLPAELSKHTKEPQATAEPPSQEKQPCKEPPIAAHPSEPSKEKAPGKEIPVVVTPLPAHQVAPSTQEKHPVSAPEQAPHERAPGKE
TQATQEKQPAKEPQVAHERAPGKETQATQEKVPAKEPQVAHERAPGKETQATQEKVPAKEPQVAHERAPGKEPQATQERVPAKEPQVAHERAPCKEPQAT
QERVPAKEPQVAHERAPGKEPQATQERTPAKEPQVAHERAPGKEPQATQERTPAKEPQVAHERAPSKETQATQERVPAKEPQVAHERAPGKETQATQERV
PAKEPQVAHERAPGKETQATQERTPAKETQTTQEKVPSKAPPCAPCDQEKAPGKHALSTTQDKAPCKEIPEPSKEPQAATQERGAGKEPQAAAQERGAGK
EPQSAAQERGAGKEPQAATQERGAGKEPQAATQEKAPGKEIPVVVVPEKKPPSKEPTEAKGQPPLDHQQRKHTSPWPLQNK

>Gj_EDPQ1
MSSSRPVDCCFPPQRYCPPVQKYCPPVQQYCPPVKSSPSQRKSCPPPCPPVKCNPPQRKYLPPPCPLTKCSTPQQKFCPPPCCPPQRKFCPPPCSSPQRK
YCPPPCCPPQRKYCPPLCCPPQQKYFPPPCCPPQRKYCPPPRFPPQQKYCPPVQQCSPPVQQTSVPVSQYCPPPQQTCCSGRQPDICQMEQVFQAPSQLL
KK

>Gj_EDPQ3
MAYPYQEQCKQPCLPPPLCKTTCPPPQPICPPKEKGCPPPQQPCSPQEKGCPPPQQPCSPQEKGCPPPQKVCPPQE

>Gj_EDPQ4
MHCACRNCNCRDCCSGNGVHLIIIYCCPPCCGCRYRCSPRCIPRQRCCARMRPCGVPSPKPCSAPSCPPPQSRCPPTHPPPQSTTLPPPKPCSSSSCPPP
QSSTLPSPKEYCLPSSSPSKSGPPPQLRSSPSSPPPQPSCPPPCPPPQSPTCPPPQVSSAPVKESCDTVEKP

>Gj_EDPSQ
MSCYQQQCKQPCLPPPCVACSPCVKTCSPNCVESCNPCAPKCEEPLSHPCPPCQCVNVCHPSVPNCVEPGHSFPPCQCVKVFHPCSSKGVEPCHPCCPRQ
CVVCQPCVPRCEESYNPSAMGMETHHPCVPKCVEPCHQCPPRQCVKVCHSYAPKCVEGCHPCAPNCVEHCHPCAPKCVVPFHPCTPKCEEPCHLSAPKCV
EPCNPCTPKCEQPCNTCGPTCVETYNPHGPMCEESCNPRGPMCEESCNPCTPKSEEHCNPCGSKCVEPCNLCGPKCENPCDTHGPRCEDLCKPGGLKCVE
LCKPCGPKCVEPSNQYGPKCEDPCKPHGPKCEDPCKPRGPKRVEEDGEEEIGFIPRPSLPKGVSERLTNSFPFPSPQQTPCEVVGAERVPLFFKNSCART
MACPRSSSRCNWRSGESNPVLPD

>Gj_EDPSOQ_partial
MSSSICSDCSDCPSHGRVTTGPCTGIQRCGKLQCSTVYRSPYHQTHFYHDPPCYSMSSRTPSGCTFQYHVPSSYPAGSKSVPQTTSTYSRMEKITCSRRQ
TYETPQQTFSKAPAESCAPFQELTCASPTQQRGPSRQSNCSVPGLSCGPSQRSACSPPVQSCSQSQQSTCSTTMQSCGPSQQSACSAPVQTCGSSQQSSC
SAPMQSCGSYQKSTCTAPMQSCGQSQQTTCSGPVQSYGTSQQSTCSGPMKSCGPSQQSPCSAPVQSSGPSQQSTCGTPMQSYGPSQQPTRSTPGLSCGPS
QKSSCSTPMQSCCPSQQSTRSTPMQSCGKSQQSTCSNPVQSCHPSQQSTCSGPMQSCGTSQQSSCSGPMQSCGPSQQSTPSTPMQSCGKSQQSTCSNPVQ
SCHPSQQTTCSVPVQSYGPSQQSTCSGPLQSCGPSQQSTPSTPMQSCGPSQQSTCSGPVQSCGPSQQSTSSTPMQSSGPSQESTCSGPMQSCGPSQQSFC
SAPMQSCGLSQQSTCSGPVQSSGPSQKSTCSTPMHSSGPFQQSTCGTPKQPCGPSQQSTCSGPMQSCGPSQQSTCSGPMQSCGPSQQSTCSTPMXXXQSC
GPSQQSTCSGPMHPCGPSQQSTCSGPMQSCGPSQQSTCSTPMQSCGPSQKSTCRTPMQSCGRXXXQQSSCSGPMHSSGPSQQSTCRTPMQSCGPSQQSSC
STSGQSCGPSQQSTCSAPMHYSDPSQQSSCSTPRQICGPSQQPTCTTPMQSCHPSQQTTSSGPVHSCGPSQQSTCSAPKQSCGPSQQSSCSTPVQSCGPP
QESTCSGPMQSCAPSQYSASSAATHSCGHSQQSTCSSPAHSRGETQQSACSSHPQSSGAPQPSN

>Gj_EDQK
MEKDQQQKKATSQPPAQQKKESQNPPKDKEKEKEGNKEEKPASDKPSG

>Gj_EDQL1
MCSRRDKYSSYNRGGSSCCGSGDNFGYGSYGSSCSGSRDNFGYGAYGSSCCGSRGSFGYGVYPSPSWSQQPAAFIPSQGLKQPAAFIPPKGLKQPAAFIP
PQGLKQPPVFVQPPLQTQPPMYAQPPLQTQPPMYAQPPLQTQPPMYAQPPLQTQPAETFIPPPVQPVFQPTMRQQVKQPPQWPSMLK

>Gj_EDQL2
MCSRRQDKECCSRVPKESSCGGRSSCSDKERSSCCGSRDNTGYVVHPVPCWPQQPAVVVPAPGAKQRPVFIPGQQQPPVLIPDQQQPPVLIPPPAQPPFQ
SQMKQQVKQPPQWPSQLK

>Gj_EDQL3
MCSRRQDKECCSRVPKESSCGGRSSCSDKERSSCCGSRDNVGYVVHPVPSWSQQPAVVVPACNAKQPPMLIPDQKQPPVFIPDQQQPAVLIPPPVAPTFV
PQMKQQVKQPPQWPSQLK

>Gj_EDQL4
MCSRRQDKECCSRVPKESSCGGRSSCSDKERSSCCGSRDNVGYVVHPVPCWSQQPAVVAPACNAKQPPVLIPDQKQPPVFIPDQQQPAVLIPPPALPPFQ
PQMKQQVKQPPQWPSQLK

>Gj_EDQL5
MCSRHESGCCGSLCCDASCCSCGCSCCESCCCKPRVKSCKQANQQSSTATPCCPLRPPPCKQQHQQKQQVKQPCKCPPPQQK

>Gj_EDQL6
MGCHGESSGCHRGSSCHGSHVQEVSMGKQFGYYGGGGCCHRSGSSCHSSGSSCHGSGSCCHHGGAQKYFPIQYCPPSYPIYSQYKQACAYPTQYLK

>Gj_EDQM1
MCSRQEKDHCHKQEKEESSCCSSRRSSCSDKESSCGRGRRDSGGCHSSRGSSCCTSGVPQQQQQHQQQQKQVCQPPCQKLK

>Gj_EDQM2
MCSRQEKDHCHKQEKEESSCCSSRRSSCSDKESSCGRGRRDSGGCHGSRGSSCCTSGVPQQQQQQQQQQKQVCQPPCQKLK

>Gj_EDQM3
MCSRQEKDHCHKQEKEESSCCSSRRSSCSDKESSCGRGRRDSGGCHSSRWSSCCASGVPQQQQQQQHQQQQKQVCQPPCQKQK

>Gj_EDQM4
MCSRQEKDHCHKQEKEESSCCLSRRSSGRDKESSCGRGRRDSGGCHSNQGSSGCCAGKPQMQQQKQQKQQVFQVPGQKLK

>Gj_EDQSG1
MSYQQSQQFKQSSACSSSSYTKPCQPSGCSKPGPAPGCSNPCPSSGCSKPGPAPGWSHPGPAPGWSNPCQSSGCSKPGPASGCANPCPPPAHQGSAGSPC
QGSWGNPSQCSSNQGSRHHQHHYYHGTQQHKSC

>Gj_EDQSG2
MSYQQSQQFKQSSACTSSSYTKPCQPSGCSKPGPAPGCSNPCPSSGCSKPGPAPGWSNPCPSSGWSKRAPASGCANPCPPPAHQGSAGSPCQGSWGNPSQ
CSSGQGSGHHQHHYYHGTQQHKSC

>Gj_EDQSG3
MSYQQSQQFKQSSPCPSSSYTKPCQPSGCSKPGQASGCSNPCPSSGCSKPVPVPGCSNPCPSSGCSKPGPAPGWSNPCPSSGCSKPGQFSGCANPCPPPA
HQGSTGSPCQGSWGNPSQCSCGQGSGQHQHHYYHGTQQPKPC

>Gj_EDSC1
MSQQQMRCSCDRCQCGCCSGVTGSSQGSQMSPGSTGECCMPVLRPHSRSGGCCGGSRSGCCHNSGSSGGCCCGCRSGSCGRCCGSGRCCSGGYGSGGGRR
GSGGGSGGGGRCGSGGDSSKGGSGGGRRGSGGGNDGGGYGGSCGGGRRGSGGGSSKGGSGGGRRGSGGGSDGGGYGGSCGGGRRGSSGGSKGGGSGRDTQ
QQQQKKC

>Gj_EDSC2
MSQQQQQGASCCCCSCCGGGSGGRGGCCGGGSSAAQQSQRSGGCCGGRSGGSGGCCGGRSRGSGGCCGCCGGCCGGRSGGGCCGGGYSQQQQLKVPSQKL
K

>Gj_EDSC3
MSQQQQRGGSCCCCSCCGGGSGGHGGCCGGGSSSSQQSQRSGGCCGGRSGGSSRCCGCCGGRSSGGCCGGGSSQQKQQQIPPRKLK

>Gj_EDSC4
MSQQQQRGASCCCCSCCGGGSGGHGGCCGGGASAAQQSQRSGGYCGGRSGGSGGCCGGRSRGSGGCCGCCGGCCGGRSGGGCCGGGYSQQQQLKVPSQKL
K

>Gj_EDSC5
MSQQQQQGASCCCCSCCGGGSGGRGGCCGGGSSAAQQSQRSGGCCGGRSGGSGGCCGGRSRGSGGCCGCCGGCCGGRSGGGCCGGGYSQQQQLKVPSQKL
K

>Gj_EDSC6
MSQQQQRGASCCCCSCCGGGSGGRGGCCGGGASAALQSQRSGGCCGGRSGGSGGCCGGRSRGSGGCCGCCGGCCGGRSGGGCCGGSYSQQQQLKVPSQKL
K

>Gj_EDSC7
MSQQQQRGASCCCCSCCGGGSGGRGGCCGGGSSAALQSQRSGGCCGGRSGGSGGCCGGRSRGSGGCCGCCGGCCGGRSGGGCCGGGYSQQQQLKVPSQKL
K

>Gj_EDSC8
MSQQQQQRGGCCCCCGGGSSSSQQSQRSGGGCCGGGSSQQGQRSGGGCCGGRSSSSQQSQGSGGCCGGSSCGGSSQMKQQKCAPQQLK

>Gj_EDSCP1
MVSCCGVPSCYIPCRSIVPMCGGQLCCAPSCTIPCGPIFPLCGGQSCCVPSCSTPCRSIAPLCGGQSCCAPSCSIPCGPIAPLCKSQEPSCSTPCSSITP
LSGGRSCCAPSCSIPCGPIAPLCGGQSCCAPSCSTPCRSIAPLYGGQSCCVPSCSIPSGPIFPLCGGQSCCAPSCSNPCRSITPLCGGQSCCAPSCSIPC
GPISPLCGGQSCCAPSCSNPCRSITPLCGGQSCCAPSCSIPCGPIAPLCSSPSCCVPSCSTPCRSIAPLYGGQSCCVPSCSIPSGPIFPLCGGQSCCAPS
CSTSCRSIAPLYSGKSHCAPSCSIPCGPVAPRCGGQSCCAPVCPIPCGPIAPLCGGQSCCVPSCTIPCGSITPLCGGQSCCDPSCCIPCSPIAIAPLCSS
HSCCVPSCSIPCRISTPSRCYTPC

>Gj_EDSCP2
MAFCRGVPSCSIPARCISPLYSVQSGSIPSRAIQCGPLAPLCGIQHRSISPYSFQCGSIRPLCNIPSCGVSPCVIPVRSVAPLCGVPSPSIPSCTIQNRP
VCSTFPGGSIAPLCSVPSSSTPSCTIPCEPIVPLCGAPSPSMPVYTVPCGPTTPLCGVPSPCPQSCTSPLCVVPSSSTPSYTIPCEPIVPLCGAPSPSMP
IYTVPCGPTTPLCGVPSPCPQSCTSPFCVVPSSSALSCTTPCEPLIPLCNVPSSSIPSYTVPSGSIIPLYSAQSACSPAYTVPLGPIIPLCSAEPSSLPS
CTIPCEPMTPFCSGPSSGIPSCTIPSGLIYPLHSASPCVPSYCPC

>Gj_EDSPRL1
MACPFQQCKQPCLPPPICGCTKQSAPVGPLPCPEPCIPVSVKQSPTTVCPEPCVKVDPCAPQCPEECAPKSVQPCAPTVCEQQCPPQSAKVCSPKSVVPC
PPPVCEQPCPPQGVEVCPPKSVVPCAPPVCEQPCPPQCEQVRASKSVPCPPPVCKEPCPPQGIDVCPPKSVVPCPPPVCEQPCPPQGVEVCPPKSVVPCP

PPVCEQPCPPQCEQVRASKSVPCPPPVCEQPCPPQGVEVCPPKSVVPCPPPVCEQPCPPPCEQVCAPKSVPCPPPVCKEPCPPQGIDVCPPKSVVPCPPP
VCEQPCPPKCEVVCPPQDIAQCKLSQCKCPPQIPPQCAPKSSK

>Gj_EDSPRL2
MSQCKQTPQLPPRCEPSCPAKDPPQIQMKTPPHPIHCQPPETVVKSLPQPKQPSKECC

>Gj_EDSPR1
MSCYQEQCQQPCLLPPCVKTCFPCVKTCSAKCVDPCCPCAPKCVEPCHPCASMCVEPCHPYPPCQYMEVCHTCAPRCVDPCNSCLPCQCVAVSQFHCAPP
CEPACVPCVPACADPCAPQCLKMCSNTCMEPCVSMCK

>Gj_EDSPR2
MSCHQHQCKQPCLPPPCLKTCSPCVKTCSPKCAEPCNPCAPRCVQPCNPCAPKCEEPCNPYASVCVEPCHPCPPRQCVEVCQPQCHSPCEPACIPCVPAC
PPCAPACAAPCAPQCVERCTTTCMEPCVSKCK

>Gj_EDSPR3
MSCHQHQCKQPCLPPPCVKTCSPKCVDLCNPCAPKCMEPCDPCAPKCKDPCHPCAPKCVDRCHPCAPKCMDPCHPCAPKCVELCHPCPPRQCVEVCQPRC
HQPCEPAGVLCAPPCSPCAPACAAPCAPQRVELCAKTCTEPFVSECQ

>Gj_EDSPR4
MSCYQHQCKQPCLPPSCVKMCSSRCVDPCNPCAPRCVDPCDPCAPKCVDPCNPCAPKCVDPCNPCTPKCVQPCNPCAPKCVGPCNPCAPKCVQPCNPCAP
KFVDPCNPCAPKCVQPCHTYPPCQCSQPCGPACVSCVPACSPCAPACVTPCAPRCMKTCSTPCAKPCVSICK

>Gj_EDSPR5
MSYHQHQCKQPCLPPPCVKTCSPKCVEPCHPCAPKCAEPCYPCAPKCVQPCNPCPPRQYVEVCQPQYSQPCGPVCAPPCAPTCSPCAPACATPCAPQYVK
PCTTTCLESCVYKWK

>Gj_EDSPR6
MSCHQHQCKQPCLPPPCVKTCSPCVKTCAPKCVEPCHPCAPKCAEPCNPYASSKCMEPCHPCPPRQCVDVCQPQCSQPCGPACVPCAPACPPCAPARAVL
CATECVRTCTTTCVEPCVSKCK

>Gj_EDSPR7
MSCHQHQYKQLCRLPPCVKTCSPCLKTCSPRCVDPCCSCATKCVEPCNPCAPKCVAPCNPCAPKCVAPCNPCAPKCVDPCNPCAPKCVAPCNPCAPKCAQ
PCYPCPPRQCVEVCQPCDLACVPCGPAWAAPCAPQCVETCSSTYLEPCAYKCI

>Gj_EDSPR8
MSCHQFKQPCLPPPCVKTCSPCVKTCSPKCVDPCHPCAPKCMEPCCPCAPKCVEPCHPCAPKCVDLCHPCAPKCMDPCHPCAPNCVEPCRSCPPRQCVEV
CQPRCHQPCEPVCVPCVPPCPPCAPACAAPCAPQCVKMCSTTCVEPCVSKCQ

>Gj_EDSPR9
MSFYQQQCKQPCLPPPCVKTCSPCAKTCSPQCVEPCHPCAPKCVAPCNPCAPKCVQPCNPCAPQCVQPCNPCAPKCVAPCNPCAPKCVEPCNPCAPKCVA
PCNPCAPKCVKVCQTQCAQTCVPACAPSCAPACSPCAPQCVKKCTTTCVEPCVPKCK

>Gj_EDSQ
MSYQWKQPCMPPPIGVKGCTKCGSVYCPGRAAPSPVAPPLKKSASACSCPCANVCTSPISPICMANGQGQCSPVCPHGPICLSPICASSASPCSNPCPGP
AKSVCMTNGQAQCAPACPHGSICVTPLQTQNGGPCANACATPKQGQCSPIYMGPGVSPSVTPQSPCDPACNYARGPICVTSIQPQRCSVCANKCPACCGS
TCAEPIKPQSSASCAATCTTPSDAVKKQCATACKDPCDSACATPCSPTCAAPGGTKGGSVGTDSCAPTTCAVSNGTKGGSVCTNSCAPTTCAVSNGTKGG
SACTKSCEEVKSGATKGAKPCVSSGPEKAWKKTRPTITLNQCVIKK

>Gj_EDSRWM
MSDYKYQSQCDPCRRKSISSGSGSGGYGQSKGASSGSGSGGYGQSKGASSGSGSGGYGQRMGASSGSGFGGYGRCIGAGSGSGSGGYGRCIGASSGSGYG
GYGQRMGGSSGSGSGGYGRCIGASSGSGYGGYGQRMGGSSGSGSGGYGQRMGGSSGSGSGGYGRCIGASSGSGYGGYGQRMGGSSGSGSGGYGRCIGASS
GSGYGGYGQRIGASSASCYGGYGQRIRCIPSYGPSCYPPIYWGGYGRSCYPPIYLGGYGSSGSPQTSYGGMGGSRSSQTSYGGYGGRCLPQTYYGGYGDG
RGSTQIQYYGGCGYGGLSGRRSSCYGLGYGLRHGSICYNPCGYSSGVCYPLSRRYSCSSIDGPW

>Gj_EDWM1
MPEERIYSSGREPYFNLNSTWYDPAGSWLDTRRKPFRYADNTCCITGCNRRDDIPRRGGHDYRCYGYRQSTCRSGGNPRVRCCVHNPSGGPRDYWGRPIG
DACNGNTGGHYSNEESVSGKPCCGSSGGCGSEGTGGCSQPCSKSVGCSSGGKGICSKAGGYGSGGRGVCSAPGGCGGGGGGVCSEAGGCSGRRRGLCSEP
CGRLLRRFQKSRRSACK

>Gj_EDWM2
MPEERIYSSGREPYFNLNSTWYGPAGSWLDTRRKPFHYAHNTCCITGCNRRDDIPRRGGHDYRCYGYRRSTCRSGGNPRVRCCVHNPSGGPRDYWGRPIG
DSCNGNTGGCYSNEESFSAGACCGSSGGCGSGGAGGCSQPGFTSVGCSGGGGGVCSEPGGCGSGGRGVCSEPGGCGSGGRGVCSEPGGCGSGGRGVCSEP
GGCGSGGRGVCSEPGGCGSGGRGVCSEPGGCGSGGRGVCSEPGGCGSGGSGACAEAGGCSGRRRGVCSEPCRRLFRRFRRSGRSSCGGC

>Gj_EDWM3
MTCSSGRESYFNLNSTWYDPAGSWLDNRRTPFCYVENTACVPCSDNRIPRRGGHDYRCYCGQPLCTGSALEYCRCGGSCGVRRPSGGCRDYWGRPVSNGC
DLCSECQNGGRPNCARRCCEPQGECRNDRSALCTE

>Gj_EDY1
MSFYDHSSCYNPCYQPCYQPSYEPCYQPCDFGRYGRRESGYDPCRYSYGSGGYDCYPRGCGGTTYVGPCNYGYRYTRRGSCYDPSSFRMGYGARLCYPSR
RCSCGSFGPL

```
>Gj_EDYA
MSCYQQQSKQPCLPPPWVKTFSHSVKTSSPKGADDYDSYAPKGVAPSPPCAPKCVDHYHSHALKCVAPSSPCAPNCVDHYHSYAPKCVVPSHQCAPKCVD
HYHSYASKSVESSYPCASKGAETSHPCAPKYLDDGRSCASKSVEPSHPCAPNCVDHYHSYASKGVEPSHPCAQKCVESSHPYSTRYYVKLGHQPQCSQSY
EPARAPSCAPACPSCTPAYASSYAPQCSKKYTTTCVETSGSKYKQWK

>Gj_EDYM1
MASYTHHCRIQHCYPAQVPGMACQAPQSFMLKHATCITRNPQPTAAKCPEPCGLKCDAKDNEPSTPKSPAPCDAKCLEPCDEKHAAPCAKPCREPCAIKP
PPPQYMERFNMPYPPPHYMHPCAAQYPPLCAPSFGMPQTFPSSSRCFPPRQMYQYTASKTYKSCYAK

>Gj_EDYM2
MTYYRQRNTMSLPFVKKGPMYFGPRYVPLYGSKYPLSICIPAHDERRCAPPRPPPYPTTRYQPRRVSVCPEPTTTKYPEVNLTRKLARASLRSPRSFLTY
SGMTRVRHGTIAKGLETHVNRIPLPHMTKGPVTYSTKTPKPYVNNDLLPHLTKDPVLYSTSTPNPSENKVPLPRMTKGPVIYATKGPEPYVTNSSQVRGA
KGILSRAAKHALSQVKKSTSGAARAPRLISTKVPRLGIMKPSRLWRNKISQSQSSLTKGSQPNLAKGSRVSLAKGSQPSLIPTGTYSSPKKLAKNVKIST
TGKKYSSATKWF

>Gj_LOR1
MSGQQRQSSSCCGGGGGGGGGGGGSSCCGGGSSGGGSSGQQIQGSCCCSGGSSGSSGGMQQMQGSGSSCCGGGGSSGGGGQKIIIVSGGSGGGQCCGGGG
SSGGQSSGCCVGGSSGGGSSQLKIPLFSGGGGCGGSGSGGVKVIVAGGSGGGSGCCVCGGSSSGGGGSGGGVKILGGGSGGGGGCGSSAGGIKVISGGSG
GGCGGGSSGGGVKIIGGGSGGGGGGGGSEGGVKIIGGGSGGGCGGGGGGGCGGGGSGGGVKIISGGSGGGCGGGGSGGGVKIISGGSGGGCGGGGSGGGV
KIISGGSGGGCGGGGSGGGVKIISGGSGGGCGGGGSGGGVKIISGGSGGGCGGGSSGGGVKVIGGGSSGGGGGGQSSGCCSGGGGGGSSGGKTIIISSGG
GGGGGSGGQSSGCCLGGGSSGGGGQTIIVPCGGGGGSSGGQSSGCCSQSMQQKQSCGGQSSGCCIGGGSSGGGGSGGQVIIVPSGGSSGGQSSGCCIGGG
SSGGGSGGQTIIVSPGGGSGGQSSGCCIGGGSSGGGSGGQTIIVSPGGGSGGQSSGCCIGGGSSGGGSGGQTIIVSPGGGSGGQSSGCCIGGGSSGGGSG
GQTIIVSPGGGSGGQSQSCLGGGSGGGGGGSGGQTIIVSPGGGGSSGGQSSIGGGCGGGSQSMQQKQPLVIPPSICQTKQSSQWPLGKK

>Gj_LOR2
MSQQRQTGSSCCCCGGAGCSRCRGGGSSGGGSCCGGSSGGVMQIQGVSSGCGGGGGSGGCCGGSRQRIIVVQGGQGSSCCGGGGYGGHQQIQGSGLGYYG
GSSGGAVCCSSGSGGAVCCGSSGSGGSGGGAVCCSSGGSGGGAVCCGSGGMGQSGQVIVVPAGGYGGQSSGYCGGMGSGFGGGLSQQKVPVVVVGGGSGG
GCCGGFGGGVPVVVAGSGSGGGAACCASSGSSGGGGASCCASSGSSGGGVQVVGGGSGDGGVKVVGGGSGMGGVKVVEGGSGGSGGAVCVSTGSGGSGGV
KVIGSGGSLGGGLSSGGSSGGKTIVVSGDGGSDGVCCGTGGSSGGTIVVSTGGSSGGPMMGQTKQAFSLPRHFK
```

B

```
>Gj_Crnn
MSQLLRNICSIIGAFEKYAQKDGDCSSLSKAELKQLIQREFADVIVNPNDPQTIESVLQLLDTDDDGKVDFEEFTVLVFRVARACHKKTQECQAPSEGQS
KKGEGSQQETLDEQTPGGDSSCRPTQEPQAPRDVSSCRPTVESQKPSREQKQELPKSGGQTPQTPRQEQNPTRPSSQEPSVTSPAQRKIQDQRAEQDLTQ
VAQRSLQDSTQESQTTRDSRHRGTQKPKISDRGPTQEQQVSEQRTGQPPARPHQGQGQGCVSQGEVEPQRPGRWQQPTGHQEEQHLSQREAQRSQRGQQE
VPGTNPRGSQVTEQSPSRGRARQPQNPEQRQGCRAEGEQKPQVGVPEQASGHQQRETLTQRERPTQQQEYHPQGSNQSKFLPGRQVPEQTTSGRRECGGS
SASEQQLHQQERRPQSPEPTPTCRPDGQSPQQERRPQSQGQSPTCRPDSQSPQQERRPQSQDQNPTCRPQGQSPQQERRPQSQEQSPTCRPQGQSPQQER
RPQSQEQSPMCRPQGQSPQQERRPQSQEQNPTCRPQGQSPQQERRPQNPVQAPTCRPEREPQVPQQTTSSRQECGSWTTEQQPHQQDRRHQDRDQAPMCR
PEQEHQVPQQTPSGPQEGASSTEEQCRQRQHGPQKPGEIQFCYPECEPQSSEQGPSPQDQWVSQARESNPSCDPAEVTQDPEHSSTRKPLKPASHQKPLQ
FPSPWSPKQ

>Gj_Scfn
MPHLLDSACTIIGVFHKYAKKDGDGFTLDRREMKRLMRKEFSEILENPRDPQTVELILQPLDHNKDSLVEFYEFVTLMFRVVKACYSHLKPESGARGALH
AGGPQRAERIHRQPRDNGDERTDGHERGGSDQTRSRPAESSTGREERREYLSPVQPVGQTDERGRRVHNHEPRGDQWRIHHSREPQEQEDDEGLPPDSQP
RADVETHQRASKPEPQWNKAGYHQPREREPTVEEEPQRQSQDLKARNDRRRPSCESQPREEDRDHYSQEPTRRVRQERQCRPRESEQQVGEDNLRRPSEF
EPQEYERSSCRMRAPGEVRNGYERRRPSHEPQAWEDERRGEYPHVLDPYEDEWGSYQSHGYEPVDGYEELPRTDGRHEPEAREYDKRRQRYHYIPEPRES
EGSRSRSRESDPREGNRRRFQPREQPREDDGRRQQYSHPEPRWVDGRRTERHDIEPRVEERRWPQTRDAETVEGERRRPRDAEPSEGERRRPHDAESNEG
ERRRPQPREQEPREDDQRRQQYSHSEPRGEERRWPQTRDAEAVEGDRRSHDAEPSEKERRLRDAEPSERERRPHDAEPSEGERRRPQPREQEPREVDWRR
QQYSHSEPRGEERRWPQTRDAEAVEGERRRSRDAEPSERERRRSCVAEPSEGERRRPHDAASSEGERRRPQPREQEPREDDRRRQQYSHSEPRGEERRWP
QTRDAEAFEGERRRPRDAEPSEGERRKPHDAEPSEGERRRPHDTEPSEGERRRPRDAEPSEGERRRPQPRAQEPREVDWRRLQYSHSEPQRVDRRRAEPH
DAHDAEAGEGERRRPRGADPREEERRSLQAQREGGQRSLRSSEHEPRETESSRRRPQRYQPEPREGERRRPQPSEHEPRGEEEERRPQPHQARPRVDERK
RLRSSEPLTREDVRSQSWPRDSEPREDERRRPRSREPESRGSESARPEARDPRGDYEPRSQYYYDPRPREREQNRYYYYSSESNPRECNQNRPSTDETEF
RNSEWGTQVPREPEYIYYGQIYYECKPREDEMSRPQIRDPKARDWERNQQEPRVSELYSVRQPQPREPESRQDERRRTHVPREPESRDGERNRLGYYAEE
DDHEIELPLHDPEQREDDQRRSRPRQSESQDGENRQLPQENAPREDEQRSSGSRQRGSRDTENGGQQTHDCAVQDEHQRPQQYRDEPREGEQRRLHQHES
EPKDIEQRRTQPHDSETREDTQRRSQQRESAPSEDEQRRSHQPKSAPREVEEQRRSQEPESEQREDEQRRSQQHESAPREDEQRRSHQHESSPREDEQRR
SHQHESAPREVEEQRRSQEPESEQREAEQRRSQQRDTQQTEVDQRGSQQHESESREGEQRRSQHNESKPTESEQRRPPSHETPATGVRRLPPTDPESSAY
GQQLQQLRKPKAEESEMGRPRLPRATESGEGKQRAEKSRESGPSQKVQTGQGGQDSPVPRSRGVERPQTGGPESQRSEQGRQLLQEPGSRAQPRDSQTTE
VVQSRSQSGEPKPDPRAAIETKGSKLVVRKGTREQSGTPETERAPVADVSRGEGSGRSVPMETDGSSQEVQEFQPPLKEENQAGTKKPLRGEHSKRDPET
RDTNASREKTELPSESKVSFVSNPLYEYLVAQKK
```

C

```
>Gj_EDKM
MSKFIKALTDMMQGNSKNSHKKVTEPEKFRRSEFKNLIQQEIVPVKRSHSNKYKHMKNLPDSDSELMNDKEITPCVY

>Gj_PGLYRP3
MTVMVEITFFVLCALSQANGCFQLITPSRWGARPANCSAPLKDVLPENVVIIHTAGTPCQTREECSRETRNVQDYHQGTKRWCDIAYSFLIGEDGYVYEG
RGWRAEGAHTLGYNDLSLGVAFIGLFTDRSPNEAAWKALKCWLDFSVKIGYLHPDYVLMAHSDVSDIVSPGEHVRQVISKWPHYKHQ
```

>Gj_S100A11
MSSKYAGGPTETERCIESLLAVFQRYAGRDGNACSLSKREFVTFMDTELASFTKNQKDPAIVDRMMKKLDMNNDGSLDFGEFLNLIGGLAQACHAQVMAS
PTSGGPKRP

>Gj_S100A12
MAQTEMEKHCEGIINGFHKFSIRVDHHDMLSIGELKQLLEQQLPTYLKKNKDAAKVLMDELDKDKNKQLDFAEFMGVITRVLIFSHDNIHKKEKEPGTVG
HSHSHGPGQGHDHSHGH

Supplementary Figure S3. Amino acid sequences of proteins encoded by EDC genes of the Japanese gecko. (A) Amino acid sequences of proteins encoded by tuatara SEDC genes excluding corneous beta proteins (CBPs), also known as beta-keratins which were reported by Liu et al. (2015). **(B)** Amino acid sequences of SFTPs. **(C)** Amino acid sequences of proteins encoded by other EDC genes of the tuatara. The following amino acid residues are highlighted to indicate residues important for protein cross-linking and the peculiar amino acid compositions of SEDCs and SFTPs and: lysine (K) and glutamine (Q) are potential sites of transglutamination; cysteine residues (C) are potential sites of disulfide bonds; glycine (G), proline (P) and serine (S) are highly abundant residues not directly involved in cross-linking. Stretches of unknown amino acid sequence due to gaps in the corresponding gene sequences are indicated by “XXX”. Only the S100A proteins whose genes are flanking *PGLYRP3* and *Scfn* are are shown here. SEDC, Simple epidermal differentiation complex gene; SFTP, S100 filaggrin-type protein; Gj, *Gekko japonicus*.

```
                  1                                                                                                  100
     Tuatara LOR1 MS--QQRHS----CGGGGGGHCGGGGGG---------------------------------------------GGGGCHGSGG----------GGGSSCH
       Gecko LOR1 MSG-QQRQSSS-CCGGGGGGGGGGGGSSCCGGGSSGGGSSGQQIQGSCCCSGGSSGSSGGMQQMQGSGSSCCGGGGSSGGGGQKIIIVS-GGSGGGQCCG
 Anole lizard LOR1 MSG-QQRQSSG-CCGRGGGGGGGGGGGSSCCGRRSGGSSSSSSSSCCCSSSRGSSGQQSQGSMCCGGGGG--GGSGGGGSGGQKIIIVSSGGSSGGGQCC
  Wall lizard LOR1 MSSMQQKQGGGSCCGGGGGGGGGGGGGGG------GGGSCCGSSGGGSISYQGQGG--SSGCCCCCGGGG--GGGGSGGGGGQKIIIVSGGGGGGGQGCC

                  101                                                                                                200
     Tuatara LOR1 SGG-------------------------------------------------------------------------------------------------
       Gecko LOR1 GGGSSGGQSSGCCVGGSSGGGSSQLKIPLFSGGGGCGGSGSGGVKVIVAGGSGGGSGCCVCGGSSSGGGGSGGGVKILGGGS-----GGGGGCGSSAGGI
 Anole lizard LOR1 CGGGGGG-------GGSSSGGGSGQII-ISSGGGGSGGQSSGSCGGGSGGGSGGGMQQK-IPCCGGGGGGGSGGIKIIGGGSSGGGGGGGGGGGGGSGGM
  Wall lizard LOR1 GGGSSGG-------GGSIGGQSSGCCI-GGGSGGGQGGGMSQQKIPYMSGGSGGGGGIC-CGGGSSGGGGGSGGVKIIGGGS--GGGGGGGGGGGSSGGI

                  201                                                                                                300
     Tuatara LOR1 ------------------------------GGGYQTQSSSCHSGGGGGGSHC----------------------QSGGGGCGGGGSSSC-----------
       Gecko LOR1 KVISGGS--------------GGGCGGGSSGGVKIIGGGSGGGGGGGSEG--GVKIIGGGSGG------GCGGGGGGGCGGGGSGGGVKIISGGS---
 Anole lizard LOR1 KSMGGGSSGGSSCGMGGGSGGGGGSGGGGGSGGVKIIGGGSSGGGGGGGGGGSGGMKSMGGGSSGGSSCGMGGGSGGGGGSGGGGGSGGIKIIGGGSSGG
  Wall lizard LOR1 KVIGGGSGGG-------GGGGGGGGGGGGSSGGIKIIGGGSGGGGGGGGGGG--------GGSGGVKIIGGGSGGGGGGGGGGGGGSGGVKIIGGGS---

                  301                                                                                                400
     Tuatara LOR1 ------HGGSSGGYQMQVGG-------------GGGGSSCH-----------GGGGGGGGYGG-------------------------------------
       Gecko LOR1 -GGGCGGGGSGGGVKIISGGS--------GGGCGGGGSGGGVKIISGGS---GGGCGGGGSGGGVKIISGGSGGGCGGGGSGGGVKIISG-GSGGGCGGG
 Anole lizard LOR1 GGGGGGGGGGSGGMKSMGGGSSGGSSCGMGGGSGGGGGSGGVKIIGGGSSSGGGGGGGGGGSGGMKSMGGGSSGGGSSGGSGGKTIIICGGSSGGGQSSG
 Wall  lizard LOR1 GGGGGGGGGSSGGIKIIGGGSGGG-----GGGGGGGGSSGGVKIIGGGS--GGGGGGGGGSSGGIKIIGGGSGGGGGGGGGGGSSGGVKIIGSGGGQSSG

                  401                                                                                                500
     Tuatara LOR1 -------------------------------------------------------SSCHGGGVSSGVYQIPSSVGGGGSS--------------------
       Gecko LOR1 SSGGGVKVIGGGSSGGGGGG-------QSSGCCSGGG--GGGSSGGKTIII----SSGGGGGGSGGQSSGCCLGGGSSGGGGQTIIVPCGGGGGSS---
 Anole lizard LOR1 GQSSGGQSSGGQSSGG-----------QSSGCCMGGGSSGSGGGGGQTIIVPCGGSSGSGG-QSSGGQSSSSCMGGGSSGGGGGGGGGGGGGGQNSGHQT
  Wall lizard LOR1 GQGSGGQSMGGGSGGGGMGGQSIGCGGQSSGSSIGGGSGGGGGSGGQTIIVPCGGSSGGGGGQSSGGYIGGGGGGSGGQSSGGCIGGGSGGGGGGSGGQT

                  501                                                                                                600
     Tuatara LOR1 ------------------------------CHGGGGGGGGSCQSGG----------------------------------------------------GG
       Gecko LOR1 -----GGQSSGCCSQSMQQKQSCGGQSSGCCIGGGSSGGGG--SGGQVIIVPSGGSSGGQSSGCCIGGGSSGGGSGGQTIIVSPGGGS-GGQSSGCCIGG
 Anole lizard LOR1 IIVSCGGSSGGGGQSSRR-RQSSGGQSSSSCMGGGSSGGGGGQSSGCCMGGGSGGGGGG-GGQTIIVPCGGSSGSGGQSSGGQSSSSCMGGGSSGG--GG
  Wall lizard LOR1 IIVPCGGSSGGGQSSGGYIGGGSGGQSSGGCIGGGG-GGSGGQSSGGYIGGGSGGGGGGSGGQTIIVPCGGSSG-GGQSSGGYIGGGS-GGQSSGSCIGG

                  601                                                                                                700
     Tuatara LOR1 GCGGGDSS------------------------------------------------------------------------------------CIDGGS--
       Gecko LOR1 GSSGGGSGGQTIIVSPGGGSGG------------QSSGCCIGGGSSGGG--SGGQTIIVSPGG-------------------GSGGQSSGCCIGGGS--
 Anole lizard LOR1 GGGGQNSGHQTIIVSCGGSSGGGGQSSGGQSSGGQSSGCCMGGGSSGGGGGSGGQTIIVPCGGSSGGGGG-----------GQSSGGQSSSCCMGGGSSS
  Wall lizard LOR1 GSGGGGSGGQTVIVPCGGSSGGGG---------GQSSGGYIGGGSGGGGGGSGGQTVIVPCGGSSGGGGGQSSGGYIGGGSGGGSGGQSSGGCIGGGS--

                  701                                                                                                800
     Tuatara LOR1 ------------------------------------------------------------------------------------ICQQAKQGGQWPDQLK
       Gecko LOR1 --SGGGSGGQTIIVSPGGGSGGQSQSCLGGG---SGGGGGGSGGQTIIVSPGGGGSSGGQSSIGGGCGGGSQSMQQKQPLVIPPSICQTKQSSQWPLGKK
 Anole lizard LOR1 GGGGGGSGGQTIIIPCGGSSGGGGGGS-------------SGGQS---SGCCSGGGG--------GSSGGSSMQSKQSFSIPACLSQTKQMNTWPSGQK
  Wall lizard LOR1 -GGGGGSGGQTIVVPCGGSSGGGGGGGQSSGCSIGGGSGGGSGGQTIIVSGGSSGGGGQGSGCCIGGGSSGGSMQTKQPVCLPPGLAQTKQSSQWPSHK
```

Supplementary Figure S4. Loricrin 1 of the tuatara is exceptionally short. Amino acid sequence alignment of the loricrin 1 proteins of tuatara (*Sphenodon punctatus*), Japanese gecko (*Gekko japonicus*), anole lizard (*Anolis carolinensis*) and wall lizard (*Podarcis muralis*). Sequences were aligned with the MultAlin program (Corpet 1988). Amino acid residues identical in all 4 or in 3 species are shown with red and blue fonts, respectively. Red dashes indicate absence of long stretches (>20 residues) of homologous sequence in the tuatara.

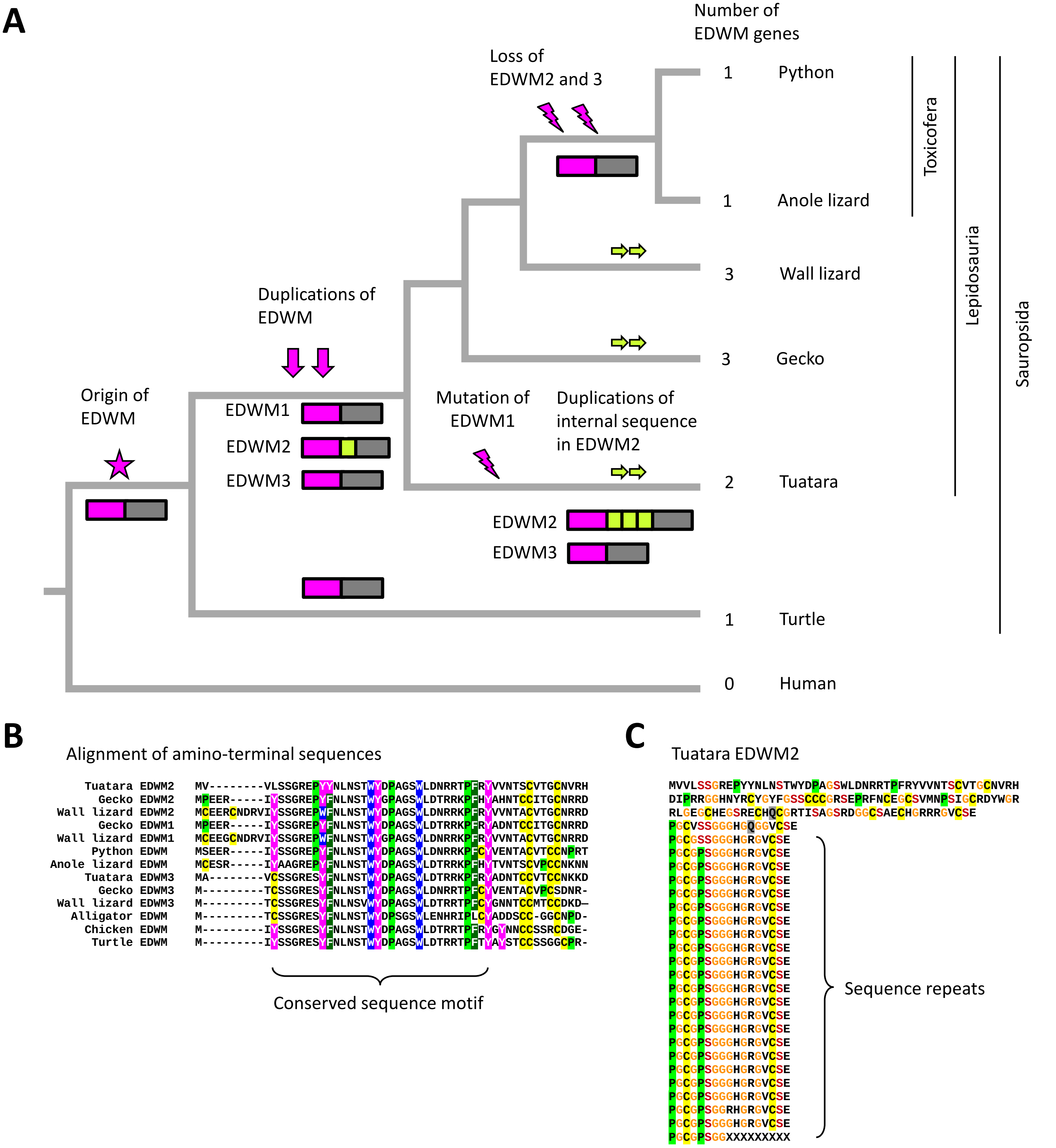

Supplementary Figure S5. Evolution of Epidermal Differentiation protein containing a WYDP Motif (EDWM) isoforms in lepidosaurs. (A) Schematic model for the evolution of EDWM. This gene is found in sauropsids (reptiles and birds) but not in mammals, suggesting an origin early in the evolution of sauropsids. Archelosaurs, here represented by a turtle, have one *EDWM* gene whereas gene duplications have led to 3 EDWM genes in lepidosaurs. Mutations have inactivated EDWM1 in the tuatara. Duplications of an internal sequence have led to extension of EDWM2 in lepidosaurs. EDWM2 and 3 have been lost in Toxicofera. **(B)** Alignment of the amino-terminal sequences of EDWM proteins. The residues WYDP are present in the center of the conserved sequence motif. **(C)** Amino acid sequence of tuatara EDWM2. Line breaks were introduced to align the sequence repeats. "X" indicates unknown amino acid residues that could not be predicted due to a gap in the genome sequence assembly. Species: tuatara (*Sphenodon punctatus*), anole lizard (*Anolis carolinensis*), Burmese python (*Python bivittatus*), Japanese gecko (*Gekko japonicus*), wall lizard (*Podarcis muralis*), alligator (*Alligator sinensis*), chicken (*Gallus gallus*), turtle (*Chrysemys picta bellii*) and human (*Homo sapiens*).

A

```
        Tuatara EDCC   MTCCGTS--SGC----------GRGT--------VDVDFGPAARMNVCPGPCCLTICGPRLITHSETLRTCCSVVS
         Gecko EDCC1   MACCGTSCGTGC----------GGGGGGSSGSGGVLVDSGPANVVSVSTGGSSLTIPGPRLQVHNN-VRVSCQCVC
         Gecko EDCC2   MACCGTSCGTGC----------GGGSGGSSGSGGVLVDSGPSNVVSVSTGGCSLTIPGPRLIVHNN-VRVSCQCVC
         Gecko EDCC3   MACCGTSCGTGC----------GGGSGSGSGSGGVLVDSGPANVVSVSTGGSSLTIPGPRLQVHNN-VRVSCQCVC
         Gecko EDCC4   MACCGTSCGTGC----------GGGSGSGSGSGGVLVDSGPANVVSVATGGSSLTIPGPRLQVHNN-VRVSCQCVC
  Anole lizard EDCC1   MSCCGGGCCGGCCGGGYGGGYGGSHGGAGG-GGGSFVGYTLGRDIHVTPGECYMITPGPYLRDCCG-VSVCCHCVC
  Anole lizard EDCC2   MACCGSGCCGDCCGGGYGG-YGGYGGGYGGDGGGVLAAVGVNKNVHVRDHGCTMVFPTPYITNNGD-FRMCCREVC

        Tuatara EDCC   GEECCRCC------SGCSGAGGCTGATGPGVSVSLQGPTYEVISGGACLRSSVHSASVQCSKTCDPC------GPC
         Gecko EDCC1   ASECCMPC------GGCCGGHG---ENGPGARVDISHPCYEVITGGGYLCSNVHCARASTSCCCDPCC-----GPC
         Gecko EDCC2   ASECCMPC------GGCCGGHG---ENGPGARVDICHPSYEVIMGGGYLCSNVHCARASTSCCCDPCC-----GPC
         Gecko EDCC3   ASECCMPC------GGCCGGHG---ENGPGARVDICHPCYDVITGGGYLCSNVHCARASTSCCCDPCC-----GPC
         Gecko EDCC4   ASECCMPC------GGCCGGHG---ENGPGARVDICHPCYDVITGGGYLCSNVHCARASTSCCCDPCC-----GPC
  Anole lizard EDCC1   VPDCC-PC-GSGGYGGYGGYGGGCGGGG--LEVDIIGESRNITMPATYLCSRVHCAE-SCCTCCEPCCC-----TC
  Anole lizard EDCC2   AHECCKPCCCGGGYGGYGGGDGGYGGQGPGLRVDMRQAGSTVHVPGSYLFSCYPCAS-SCTECCDPCCCGSCCGPC
```

B

```
     Tuatara EDQM1   MGSSNTKEQQEQSGCHENRRSSCSGSTSSYQD-----------------SGSSSCGRSSGGCQSNHGSS-------
     Tuatara EDQM2   MSCQQNRGCQQSIPKQTGCSSGGCGRSGGG--------------------GCSRRGSSGGGCSSSGGSS-------
       Gecko EDQM1   MCSRQEKDHCHKQEKEESSCCSSRRSSCSDKE-----------------SSCGRGRRDSGGCHSSRGSS-------
       Gecko EDQM2   MCSRQEKDHCHKQEKEESSCCSSRRSSCSDKE-----------------SSCGRGRRDSGGCHGSRGSS-------
       Gecko EDQM3   MCSRQEKDHCHKQEKEESSCCSSRRSSCSDKE-----------------SSCGRGRRDSGGCHSSRWSS-------
       Gecko EDQM4   MCSRQEKDHCHKQEKEESSCCLSRRSSGRDKE-----------------SSCGRGRRDSGGCHSNQGSS-------
  Anole lizard EDQM   MCSRQDKDKAYKQEKEEKPSGCGRQNSGNESR-----------------G-CGSGRQNSG-------SS-------
       Python EDQM   MCSREDKDQCYKQEKDESPCCYSHRSSGCGNK-----------------PS---SRACSGSYGWGPSSP-------
     Chicken EDQM1   MCSRQDRDQCHSQERYTRQSS-GCHSS-GGGGCHSSGGGGGCHSSGGG-GGCHSSGGGSGGCHSSGSSGGCHSSSG
     Chicken EDQM2   MCSRQDRDQCHSQERYTRQSS-GCHSS-GGGGCHSSGGGGGCHSSGGGSGGCHSSGGGGGGCHSSGSSGGCHSSSG
     Chicken EDQM3   MCSRQDKDQCHRQERSSCHSSEGCGGGRGGSGCHGSSGSM-CHSSSGG-FGCHRSSGS--GCHGSSGSG-CHGSSG

     Tuatara EDQM1   ------------------CHSVGPSCQP----------QHNQQQQQTCQVPSQKLN
     Tuatara EDQM2   --------------SGGGCGSHGK---------------QQCQQKQQCPIPQQKMK
       Gecko EDQM1   ------------------CCTSGVP---------QQQQQHQQQQKQVCQPPCQKLK
       Gecko EDQM2   ------------------CCTSGVP---------QQQQQQQQQQKQVCQPPCQKLK
       Gecko EDQM3   ------------------CCASGVP-------QQQQQQQHQQQQKQVCQPPCQKQK
       Gecko EDQM4   -----------------GCCAGKPQ-----------MQQQKQQKQQVFQVPGQKLK
  Anole lizard EDQM   -------------------C-GKPQKPKPS-----QDQQQQQQQQQVCKVPDQKQK
       Python EDQM   ------------------YCQGKSDQP-----------QQQQQKKQGCQLPPQK--
     Chicken EDQM1   GGGCHSSGGSSCH--------GKPQVQYHYYHHQQQQQQQQQQQQQVHQLPSQKMK
     Chicken EDQM2   GGGCHSSGGSSCH--------GKPQVQYHYYH-QQQQQQQQQQQQQVHQLPSQKMK
     Chicken EDQM3   SGCHGSSGGSGCHGSSRGSCHGKPQDC----------------QQQIYQV-SSKMK
```

Supplementary Figure S6. Examples of EDC proteins that are conserved in tuatara and other sauropsids. (A) Amino acid sequence alignment of Epidermal Differentiation proteins with Cysteine-Cysteine repeats (EDCC) which are lepidosaur-specific. **(B)** Amino acid sequence alignment of Epidermal Differentiation proteins containing a glutamine (Q) Motif (EDQM) which are conserved in sauropsids. Amino acid residues are colored as in supplementary figures S1 and S3. Sequences were aligned with the MultAlin program (Corpet 1988) followed by manual corrections. Species: tuatara (*Sphenodon punctatus*), anole lizard (*Anolis carolinensis*), Burmese python (*Python bivittatus*), Japanese gecko (*Gekko japonicus*) and chicken (*Gallus gallus*).

A

N-terminal sequence motif in EDSPR and other SEDC proteins

```
     Tuatara EDSPR1    MSY--QQQCKQPCLPPPI--K
     Tuatara EDSPR3    MSY--QQQCKQPCLPPPI--K
     Tuatara EDSPR5    MSY--GQQCKQPCLPPPI--K
     Tuatara EDSPR8    MSY--GQQCKQPCLPPPI--K
     Tuatara EDSPR4    MSY--GQQCKQPCLPPPI--K
     Tuatara EDSPR6    MSY--QQQCKQPCLPPPI--K
     Tuatara EDSPR7    MSY--QQQCKQPCLPPPI--K
     Tuatara EDSPR2    MSY--GQQCKQPCLPPPI--K
     Tuatara EDSPR9    MSY--GQQCKQPCLPPPI--K
       Gecko EDSPR5    MSY-HQHQCKQPCLPPP-CVK
       Gecko EDSPR1    MSC-YQEQCQQPCLLPP-CVK
       Gecko EDSPR2    MSC-HQHQCKQPCLPPP-CLK
       Gecko EDSPR6    MSC-HQHQCKQPCLPPP-CVK
       Gecko EDSPR3    MSC-HQHQCKQPCLPPP-CVK
       Gecko EDSPR8    MSC-HQF--KQPCLPPP-CVK
       Gecko EDSPR4    MSC-YQHQCKQPCLPPS-CVK
       Gecko EDSPR9    MSF-YQQQCKQPCLPPP-CVK
       Gecko EDSPR7    MSC-HQHQYKQLCRLPP-CVK
     Tuatara EDSPRL1   MSFPHQHQCKQPCLPPPICGQ
Anole lizard EDSPRL1   MACPHQ-QCKQPCLPPPLCGK
       Gecko EDSPRL1   MACPF-QQCKQPCLPPPICGC
       Gecko EDYA      MSC-YQQQSKQPCLPPP-WVK
Anole lizard EDEPT     MSY----QARQPCTAPPIYQE
       Gecko EDEPT     MAY----QCTQPSLPPPIHGQ
 Wall lizard EDEPT     MAY----QCRQPCLPPPIGVP
       Gecko EDCTT     MAY----QCKQPCVGPPSFVK
 Wall lizard EDCP      MSH----QCKQPCAPPPCCVK
Anole lizard EDCP      MSY----QCKQRCLPPPIWVK
       Gecko EDSQ      MSY----QWKQPCMPPPIGVK
Anole lizard EDSQ      MSY----QVKQASLPPPTYGK
 Wall lizard EDSQ      MSY----QCKQPCLPPPSCVK
 Wall lizard EDCATM    M-Y----QTKQPCLPPPIGAT
Anole lizard EDCATM    M-F----LSQLSCLLPPIFPT
```

B

Internal sequence repeats of EDSPR and other proline-rich SEDC proteins

```
>Sp_EDSPR1
MSYQQQCKQPCLPPPIKCGPVCQP
KCVPVCSP
KCVPVCSP
KCVPVCQP
KCVPVCQP
KCVPVCHP
KCVPVCSP
KCVPVCSP
KCVPVCSP
KCVPVCSP
KCVPVCPP
KCPPSQNCCYQHKWY

>Sp_EDSPR5
MSYGQQCKQPCLPPPI
KCVPVCPP
KCVEPCPP
PCVPVCPP
KCVPVCPP
KCVPVCPP
KCVPVCPP
KCVPVCPP
PCVPECPP
KCVPVCPP
```

KCVPVCPP
KCVEPCPP
PCVPVCPP
KCVPVCPP
KCVQPCPP
PCVPVCPP
KCVPVCPP
KCVPVCPP
KCPPSQNCCYQHKRY

>Sp_EDSPR9
MSYGQQCKQPCLPPPI
KCVPVCPP
KCVELCPP
KCVPVCPP
PCVPVCPP
KSVPVCPP
KCVDVCPP
KCPPSPNCCQKKFY

>Gj_EDSPR1
MSCYQEQCQQPCLLPPCV
KTCFPCVKTCSA
KCVDPCCPCAP
KCVEPCHPCAS
MCVEPCHPYPPC
QYMEVCHTCAP
RCVDPCNSCLPC
QCVAVSQFHCAP
PCEPACV
PCVPACADPCAP
QCLKMCSNTCMEPCVSMCK

>Gj_EDSPR5
MSYHQHQCKQPCLPPPCVKTCSP
KCVEPCHPCAP
KCAEPCYPCAP
KCVQPCNPCPPRQYVEVCQP
QYSQPCGPVCAP
PCAPTCSPCAP
ACATPCAPQYVK
PCTTTCLESCVYKWK

>Gj_EDSPR9
MSFYQQQCKQPCLPPPCVK
TCSPCAKTCSP
QCVEPCHPCAP
KCVAPCNPCAP
KCVQPCNPCAP
QCVQPCNPCAP
KCVAPCNPCAP
CVEPCNPCAP
KCVAPCNPCAP
KCVKVCQT
QCAQTCVPACAP
SCAPACSPCAP
QCVKKCTT
TCVEPCVPKCK

>Pm_EDSPR1
MSCHQHQCKQACLPP
PCVKSCQPCAPQ
QCVKTCCPPCGP
KCGPACGSGCGP
KCAPACGAGCGP
KCTPACGSGCGP
KCAPACGSGCGP
KCAPACGSGCGP
KCAPTCAPACAP
PCAPQCVKKCAT
KCVEPCTPKCK

>Cp_EDPCV1
MAYQQQCKQPCLPPPCCVKQCKT
KCVDPCPCPP
QCVDPCPPCPP
KCVDPCPP
KCVDPCPP
KCVDLCPP
KCVDQFPCPP
KCVDVCPP
KCVDLCPP
KCVDQCPCPP
KCVDVCPP
KCVDVCPP
KCVDVCPPCPPLQHCCQEKKHY

>As_EDPCV
MSFQHQCKQPCLPPP
ICGQTVPSQPCVA
PCSTVRVD
PCPPVCVN
PCPPQCVD
PCPPGCVK
PCPPQCVD
PCPPVCVN
PCPPQCVE
PCPPGCVN
PCPPQCVD
PCPPGCVN
PCPPQCVD
PCPPKCPPSQQCCAQTKLC

>Gj_EDCTT
MAYQCKQPCVGPPSFVKGSTISYVDA
DADKSSDVPPCIE
PCQAAPMDASKTA
AVGSQTAGTKASK
AAVKAADTTEVCA
KVAVGRSKVVCTE
GCGNFPTKTIKSS
TPSQAQGPTCVPV
CKTPDQGPACVPV
CTSPSSAPAYVPI
CKTPDQGPAYVPV
CTSPSKGPVCVPV
CATPGQGPTYIPV
CVAPGHAPAYVPI
CTTPGHPPTYVPV
CTSPGHPPTYVPV
CATPGQAPTCVPV
CTTPGQAPTCVPV
CTTPGQAPTSVPV
CATPGQAPTCVPV
CTTPGQAPTSVPV
CTTPGQGPTYVPV
CTTPGQGPTYVPV
CTTPGQGPTCVPV
CTTPGQGPTYVPV
CTTPGQGSTCVPV
CTTPGQAPTCVPV
CTTPGQGPTCVPV
CTTPGKAPTCIPV
YATPGHAPTCVPV
CKSPSRVPYSGMS
CKSPPSSPVWYVQ
CCRSGCGGGCGYM
FLVTHPAQGVTPYTCCSRSPSSKQG

```
>Ac_EDCPGS
MPYCQLQSNQTSAAPSGCVKSYT
TECPQLCH
PQCGPQCG
PTCGCHGG
PNCGCPCG
PNCRCPCG
PSCGCSCG
PSCGCHGG
SNCGCPCG
PSCGCPCV
PCCGCPCG
PSCGCQSG
PNCGCPCG
PSCGCRGG
PNCGCPCG
PNCKCPHN
PTCGYPCG
PSGGASCS
PICNQPCG
PSCGYPCH
PSCGPSWA
PAGGKSCG
PTCAPSGG
TSCGPSCG
KTCVPSGG
ISYGPSRK
TSCSPNCG
QTSGSSCA
ATCGSSSA
IKSEKKCDTKSKDSCSTKEK
```

Supplementary Figure S7. EDSPRs contain a conserved sequence motif at the amino terminus and multiple sequence repeats and thereby resemble other SEDC proteins. (A) Amino-terminal sequence alignment of SEDC proteins. This segment of the proteins contains a largely conserved sequence motif (Strasser et al. 2014). **(B)** Amino acid sequences of EDSPRs and other SEDC-encoded proteins. Line breaks were introduced to align the specific sequence repeats of each protein. Amino acid residues were shaded in colors as in supplementary figure S1. In addition tyrosine (Y) is highlighted in magenta in panel A. Species: tuatara (Sp, *Sphenodon punctatus*), anole lizard (Ac, *Anolis carolinensis*), wall lizard (Pm, *Podarcis muralis*), Japanese gecko (Gj, *Gekko japonicus*), turtle (Cp, *Chrysemys picta bellii*) and alligator (As, *Alligator sinensis*).

A

```
>Ac_EDGGY1
MTFYRGSWGHQSCCDPCSYGYGGWSGSRCGYPRCYEPCGYGYGYGGWCGSRCSYPYGGWGCGWGNGC

>Ac_EDGGY2
MTHSSGSCKPCGYSSCSTPCSYGGYGGYGRYCGSSKCYNPCGYGGCYGGYGGYGGCGGYGGYRGYGGYGGYGCNSGGYGYCGPNWCYPYYGRGC

>Pb_EDGGY1
MSSYHGGICRPWKYQSCYNPCGGGYGGWYGHQCYDHCGYGGWCSSRWCYPPCYPCSRWGWGWGC

>Pm_EDGGY1
MTYSCSVACRPWRHQSCYEPCGYGYGYGGWCSSRYCAPRCYEPCGYGYGGYGGYGGYGGYGDWCGSRWGYPYSRWGCGW

>Pm_EDGGY2
MTYSCSVACRPWRHQSCYEPYGYGYGYGGWCSSRYCAPRCYEPCGYGYGGYGGYGSYGCYGGYGDCCGSRWGYPYSRWGCGW

>Pm_EDGGY3
MTYYGGYCRPYGYSSCSTSCYDPCCYGSGYGHWYGSTTCRTGCYDPCRYGGYSGYGCYGGSGCYGAYGSKGCYPYSRWGCGW

>Pm_EDGGY4
MSNYGTYGLSSCSGNQSGREPSSQGSGHWFGSTTSRPRCYEPSSYGSYASSGGFGGCGSCGGGYGGSGRMGGHSGFGSFASFGGCGGQGGYGGW
GNPRPCASSPWGSGWGN
```

B

```
    Tuatara EDGGY1  MTY-GGY-GKSWSYSSRSGRCNEPCGYGSGASGGYGHWYGSTTCRTRCSEPC------------GYG-
Wall lizard EDGGY3  MTYYGGY-CRPYGYSSCSTSCYDPCCYGSG----YGHWYGSTTCRTGCYDPC------------RYG-
Wall lizard EDGGY1  MTYSCSVACRPWRHQS----CYEPCGYGYG----YGGWCSSRYCAPRCYEPC------------GYG-
Wall lizard EDGGY2  MTYSCSVACRPWRHQS----CYEPYGYGYG----YGGWCSSRYCAPRCYEPC------------GYG-
     Python EDGGY1  MSSYHGGICRPWKYQS----CYNPCGGG------YGGWYGHQ-----CYDHC------------GYGG
Anole lizard EDGGY1  MTFYRGS----WGHQSCC----DPCSY------GYGGWSGSRCGYPRCYEPC------------GYG-
    Tuatara EDGGY2  MTYSAGC-CYPWGSSSCGSSCYDPCRSGCGY--GYGRW-GSRYSGSGCCEPC------------GYGG
Anole lizard EDGGY2  MTHSSGS-CKPCGYSSCST----PCSYG-----GYGGY-GRYCGSSKCYNPC------------GYGG
      Gecko EDGGY2  MSY-GNC----CG-SSCCS----PC--------GYGGW-GRR-GWGGYGGWG------------GYGG
      Gecko EDGGY1  MSY-GNC----CGYS-CCS----PCGYGGW---GYGGW-------------------------GGYGG
      Gecko EDGGY3  MATYGMHSMSH-SSSQ-------PSHGVSGQGFGAGHWYGSSTSHPRCYEPTSYINLGGPSAGGGYGS
Wall lizard EDGGY4  MSNYGTYGLSSCSGNQ--SG-REPSSQGS------GHWFGSTTSRPRCYEPSSY---GSYASSGGFGG


    Tuatara EDGGY1  ------------------YGGYGGYGGYGGYGGNGCYGG-YNR-GCGSPRWSYPCSQGWGCKY------
Wall lizard EDGGY3  -----------------------GYSGYGCYGGSGCYGA-YGSKGC------YPYSR-WGCGW------
Wall lizard EDGGY1  ------------------------YGGYGGYGGYG---G-YGD--WCGSRWGYPYSR-WGCGW------
Wall lizard EDGGY2  ------------------------YGGYGGYGSYGCYGG-YGD--CCGSRWGYPYSR-WGCGW------
     Python EDGGY1  ----------------------------------------WCSSRWCYPPC-YPCSR-WGWGW----GC
Anole lizard EDGGY1  ----------------------------------------YGYGGWCGSRCSYPY-GGWGCGWN--GC
    Tuatara EDGGY2  -YGGGYGGYGNCCG-YGGYGGYGGYGGYGGYGGRYG------------SRCSYP-S--WGCGWDSCGSC
Anole lizard EDGGY2  CYGG-YGGYGGC-GGYGGYRGYGGYGGYGCNSGGYGY--------CG-PNWCYPYYGR-GC--------
      Gecko EDGGY2  CGG--------CCGGYGGYGGYGGYGGYGGYGGRCG----NRCGYSSCCSPCGGGWGWGW------
      Gecko EDGGY1  CGG--------CCGGYGGYGGH-------------GCCG----NRCSYSSCCSPCCGGWGWGW------
      Gecko EDGGY3  CGGPSSYGMLSCRNTSGGPGTYGG--GYGGPGGYGSPGGPGGSVSWCSPRS-Y-SSPQWGSSWGN----
Wall lizard EDGGY4  CGS--------CGGGYGGSGRMGGHSGFGSFASFGGCGGQGGYGGWGNPRPCA-SSP-WGSGWGN----
```

Supplementary Figure S8. EDGGY are lepidosaur-specific proteins rich in glycine-glycine-tyrosine motifs. (A) Amino acid sequences of EDGGY proteins of the anole lizard, the wall lizard and python identified during this study. These Epidermal Differentiation proteins are characterized by the presence of repeats containing glycine duplets (GG) followed by tyrosine (Y). **(B)** Amino acid sequence alignment of EDGGY proteins. Amino acid residues are colored as in supplementary figures S1 and S3; in addition tyrosine is highlighted in magenta. Sequences were aligned with the MultAlin program (Corpet 1988) followed by manual adjustments. Species: tuatara (*Sphenodon punctatus*), anole lizard (Ac, *Anolis carolinensis*), wall lizard (Pm, *Podarcis muralis*), Burmese python (Pb, *Python bivittatus*), Japanese gecko (*Gekko japonicus*).

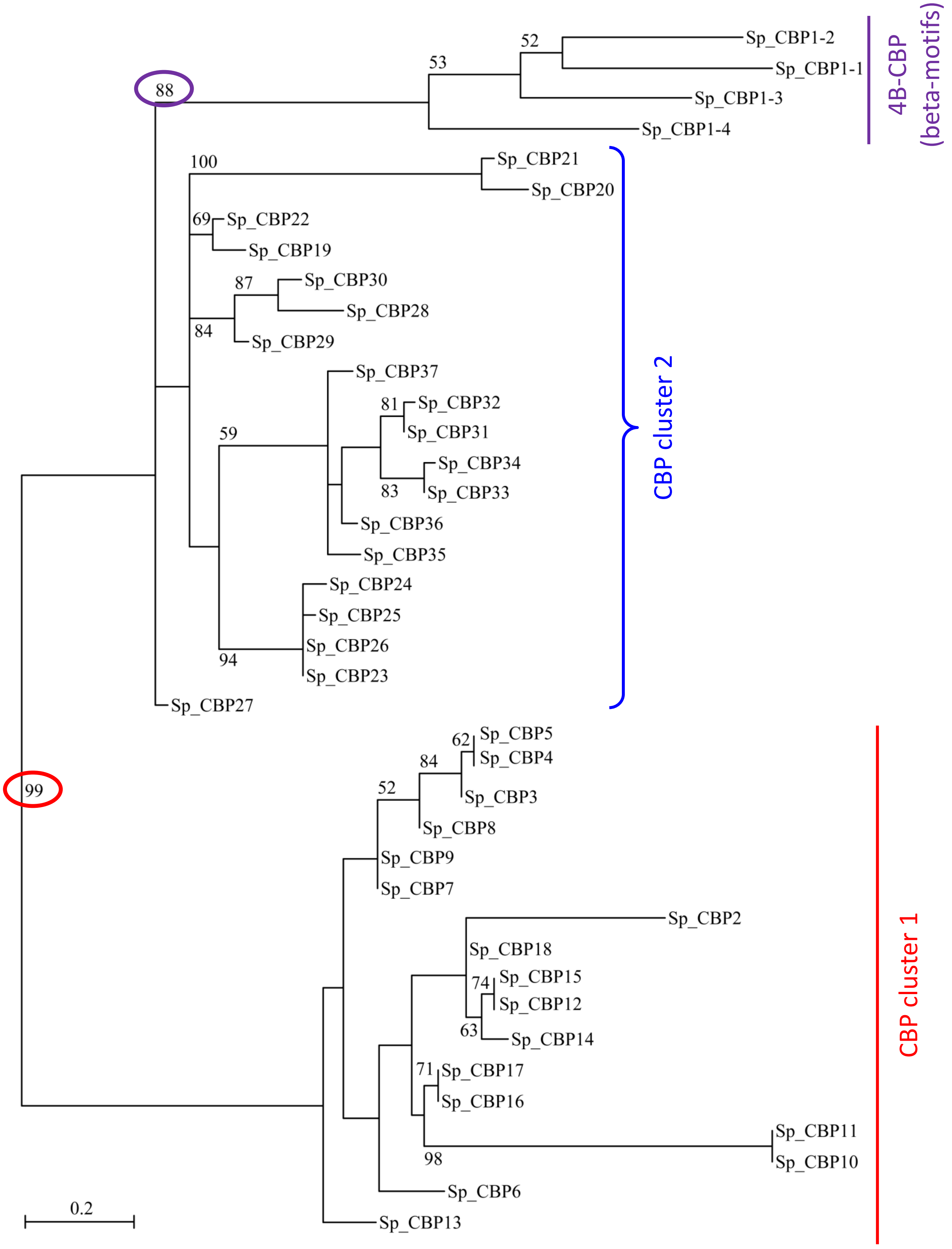

Supplementary Figure S9. Phylogenetic analysis of tuatara corneous beta proteins (CBPs). The amino acid sequences of the beta-sheet motifs of CBP proteins of the tuatara (Sp, *Sphenodon punctatus*) were aligned and a phylogenetic analysis was performed with PhyML (JTT model, 100 replicates). Bootstrap values >50 are indicated. Support values for important branches are encircled. "Cluster" refers to the arrangement of the corresponding genes within the EDC. Scale bar: substitutions per site.

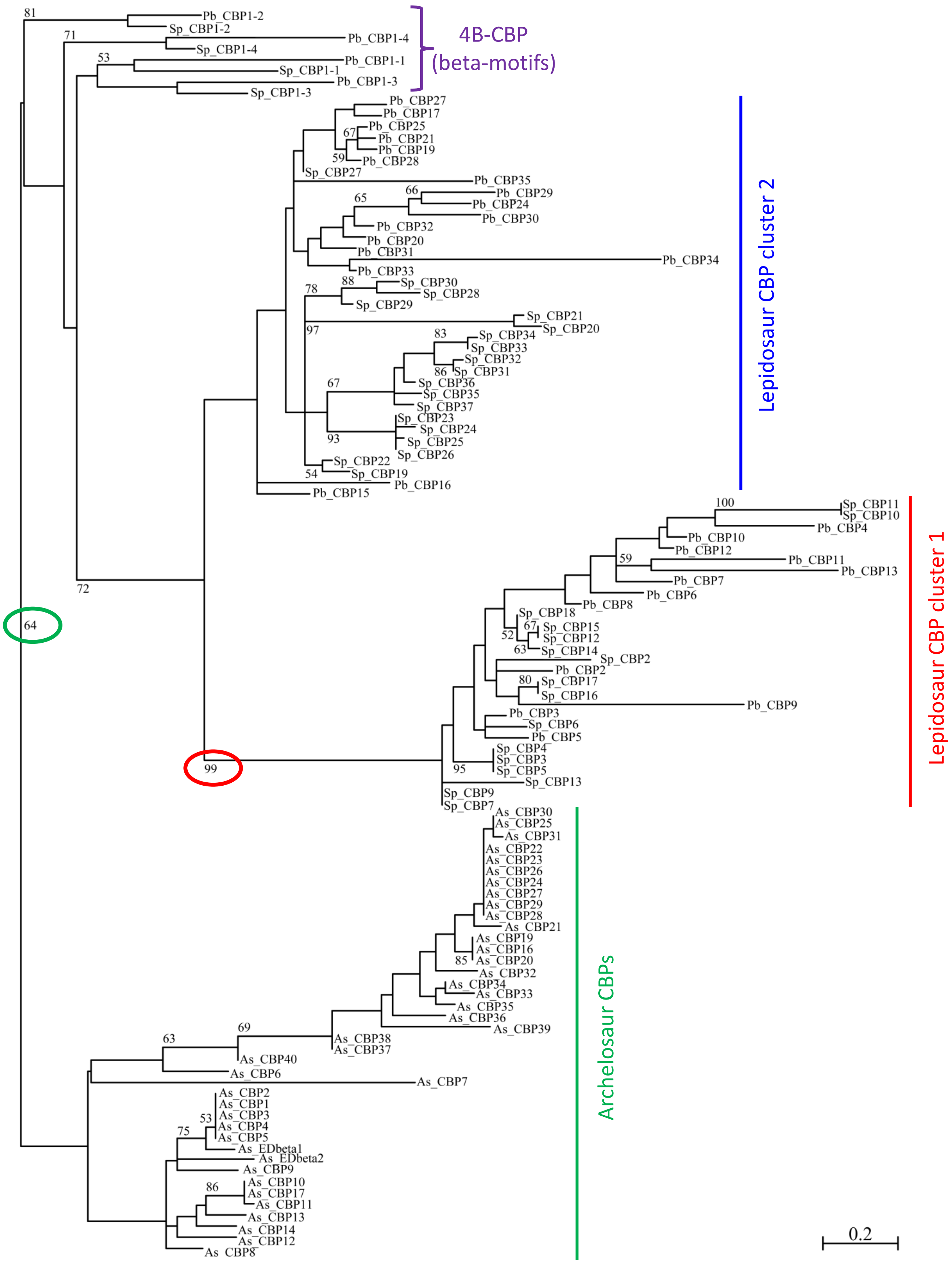

Supplementary Figure S10. Phylogenetic analysis of corneous beta proteins (CBPs) of the tuatara, python and alligator. The amino acid sequences of the beta-sheet motifs of CBPs were aligned and a phylogenetic analysis was performed with PhyML (JTT model, 100 replicates). The 4 beta-sheet motifs of 4B-CBPs were included separately and their position within CBP1 proteins is indicated by a number after a dash. Bootstrap values >50 are indicated. Support values for important branches are encircled. “Cluster” refers to the arrangement of the corresponding genes within the EDC. Species: tuatara (Sp, *Sphenodon punctatus*), Burmese python (Pb, *Python bivittatus*), Chinese alligator (As, *Alligator sinensis*). Scale bar: substitutions per site.

A

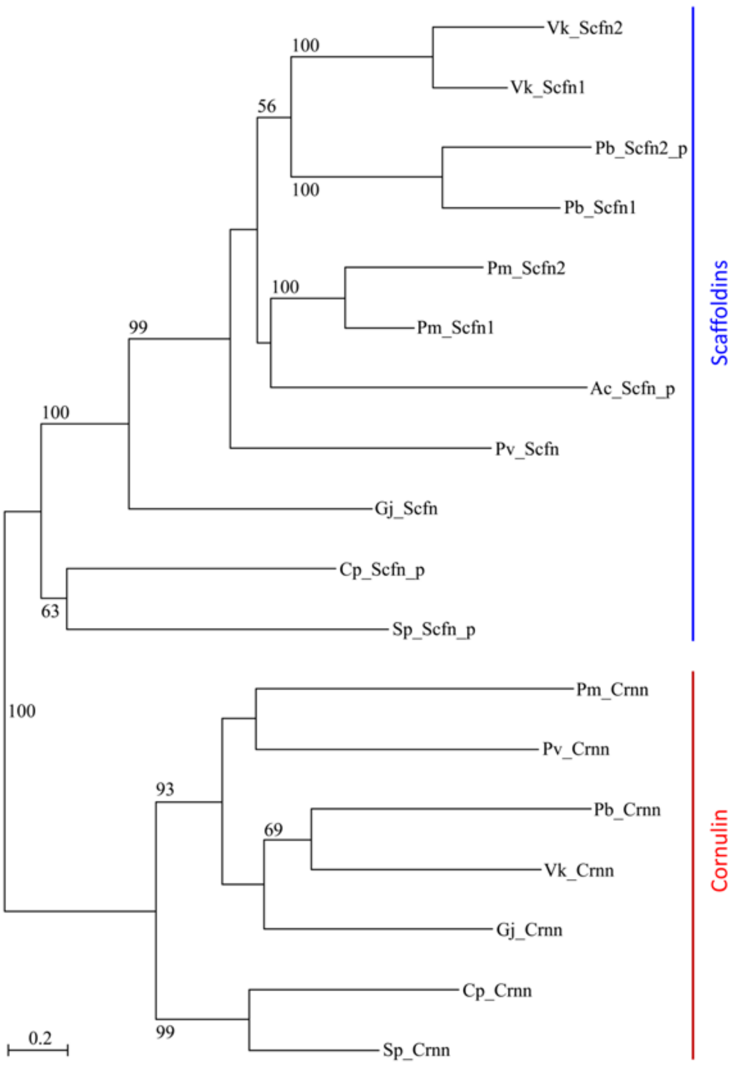

B

```
                    1                                                                                                   101
Wall lizard Scfn1   MPYLLGSICTIIGVFYKYAKRHGDCSTLNRREMKRLILKEFGEVIKNPSDPETVEMTFQLLDINGDSLVDFNEYLLLIFRVAKACYSHLQPRECLLQQGEA
Wall lizard Scfn2   MAHLLDKMCTIMGVFYKHAKQRGNCSALTRREMKTLLQEEFAGVIENPRDPETLELTFQLLDVNKDCLVDFNEYLFLIFRVAKACDSHLQPRERRIQRGEA

                    102                                                                                                 202
Wall lizard Scfn1   RRALREREARGEGRDDRHLREDEREGSYGRERRDSDRTRLHQLEAVARDEGRRESFPREAVARGVERSRHARDQELRDDRGRDRPSHEPQDSENEHEDWPR
Wall lizard Scfn2   RRAPHEDELRGEGRDHRQLREAEREGGYVCERRDSDRPRLRQQEGATRREGRRDHFPQEPVARSDQRSRQARDQDLRDDCGRHRPSRETQEWEDDCEDRPR

                    203                                                                                                 303
Wall lizard Scfn1   KSEEQADVGTRQQPLGDEPRRSDAGRRQLRRREPEMEEDHRRQSRERVQREDGERRPLLRDPRLREGGESERYSQEATQRDEDERRHQARETERQLEEEED
Wall lizard Scfn2   GSDEQGEVETCQQTHECESRRDEAGRHQLRRREPSREEDHQRQSWERDYDQ--------RDCLPRGGGDRQRHSQEATQRGEDERRRQPKVTECQMEEEED

                    304                                                                                                 404
Wall lizard Scfn1   LRRPHVSGPRIDEESHNRRRAVQEIRSDYGRTRPSREPREREDARRDDYVREAERRSSRRDQRVSDARRDQRSDREPVDYDDERHSFPREGERRSWSRDAE
Wall lizard Scfn2   LRRPRVSGQQVGEESHTRRCAAQGIRSAYGRSRPSCEPQELEDGGREDYVCEVERRSRRRDQRVADECWDQRSDRKPVDCDDECHSFRRDGERRSLSCDPE

                    405                                                                                                 505
Wall lizard Scfn1   QEQRRSQMPEEEYRERPRSSDPDYRVDEQRRR--YTAQSREAELHEPDVRESERRRRQPVDQDEWNRPYPRESTTREELEDQRGSRDPERREROWSGLHPY
Wall lizard Scfn2   REQRRSQRCDAEHTERSMS---DCRADERRSQGQYRPQSRGAELREHDVRESERRRRQPVDQDDRTRPYPCEPTTRAEVEDQRWSRVLECREGQRTGLHPS

                    506                                                                                                 606
Wall lizard Scfn1   ERKPTDGERRRPQSRESELREGDRRRSQRHAQTPE-DGRRREQYHQDDAQEEENLRRRRPLSGDLEPRDYESCRPQRRERDPRSDGQRRQQYYDPEPLDRD
Wall lizard Scfn2   ECEPADGERRRPQSCEYEPREGDRRRSQRHAQTPEEDGWGREECHQDEAQEEENLRRRRPLSGDLEPRDYESCRPQRREREPRGDGQRRQQYYDSEPLDRD

                    607                                                                                                 707
Wall lizard Scfn1   VERRMPEAPESALREGSRGRQQYSDPDTQERGSRRRPQSRYSESREDERRRPEEADFSDDERERPQARQSLSRPREDNRCYPSVGSRRECDRGRRQEYSGQ
Wall lizard Scfn2   VERRMPEAPESAQREGRRGRQQCYDSETLE-SRRNRPQSHYSESWEDERRRPEEADFSDDERERPQARQSLSRPREEDRYYSSVGGRRECDRGRRQEY---

                    708                                                                                                 808
Wall lizard Scfn1   RRNVDQSGDEQERRAPLRDTDAADDEQWRSQSRDIEPRDGEQQRRTQSRDTDGQRRSQRCNIDQSDDEQERRTPLRDTDAEDAEQRRSQSRDTEPRDSEQQ
Wall lizard Scfn2   ------------------------------------------------------------------------------------------ESEPRESE--

                    809                                                                                                 909
Wall lizard Scfn1   RRTQSRDTDGQRRSQRRTVDQSDDEQERRAPLRDTDAADDEQRRSQSRDIEPRDSEQQRRTQSRDTNGQRRSQRRTVDQSRDEQERRAPLRDIDAEDSEQR
Wall lizard Scfn2   -----------------------------------------------------------------------------------------------------
```

```
                      910                                                                                                   1010
Wall lizard Scfn1     RSQSRDIEPRDSEQRRRTQSRDTDGQRRSQRRTVDQSDDEQERRTPLRDIDTEDAEQRRPQSRDTEPRDSEQRRRTQSRDTDGQRRNVDQSRDEQERRTPL
Wall lizard Scfn2     ---------------------------------------------------------RRPRQR-CEPEDYEER---------GQRYCTPGAR-EQEGSRPQ

                      1011                                                                                                  1111
Wall lizard Scfn1     RDIDAEDTEQRRSQSRDIEPRDSEQQRRTQSRDTDGQRRSQRRTVDQSRDEQERRVPLRDIDAEDAEQRRPQSRDTEPRDSEQRRRTQSRDTDGQRRNVDQ
Wall lizard Scfn2     RSCDT------------------------------------------------------------------------------------------------

                      1112                                                                                                  1212
Wall lizard Scfn1     SRDEQERRTPLRDIDAEDTEQRRSQSRDIEPRDSEQQRRTQSRDTDGQRCTVDQSRDEQERRAPLRDIDAEDAEQRRSQSRDTEPRDREQRRRTQSRDTDG
Wall lizard Scfn2     --------------DPIGVDGRRCQTREVDPRE----------------------------------------QQRRARCRDTEQRDRERNQTLR------

                      1213                                                                                                  1313
Wall lizard Scfn1     QRRNVDQSRDEQERRTPLRDIDAEDTEQRRSQPRDIEPRDSEQQRRTQSRDTDGQRRSQRRTVDQSRDEQERRVPLRDIDAEDAEQRRSQSRDTEPRDSEQ
Wall lizard Scfn2     ----------------------------------------------------------------------------RDGEARQGDQRRNWSHDTDRRDSEQ

                      1314                                                                                                  1414
Wall lizard Scfn1     RRSTQSRDTEQRDSQQRRTQSHYSDTTDGEQRRTRSGDPRFDGQQRRAPSQDTDATDIDRRQTQARGSDPREGEQQRRPQSRGAEPADVDQKQTPTRDADP
Wall lizard Scfn2     --------------QRRTQSCADDVGDVEPRR-RGMDLGISEQQRRGPSQSSGAADADQRRVQAREEDLRES-----------------------------

                      1415                                                                                                  1515
Wall lizard Scfn1     RNDEQQRRVVSRDTEQSDGEQRQTQLHSVDARIAAARDGRQQRRTQTRDVDSRDAQQQRRMQSSDTEPTDGDQRQTQTSEAAPRERGQQRQTESRDTEQGA
Wall lizard Scfn2     ----QRRRVVPSGLEH-------------------------------------EASQQRRPHCCDSES---------------------------------

                      1516                                                                                                  1616
Wall lizard Scfn1     SREEPRYSEPKDNVQRRQAVPETQVAQPSSVRQAEPLAQDVEGSRAPPREVAPSADTARRPRPQEQEAAERVLGRQRPAESGETQQRPSERERAQSREPQR
Wall lizard Scfn2     -----------------------------------------------------------------------------------------------------

                      1617                                                                                                  1717
Wall lizard Scfn1     SDGEQGRARPGGPVPRQSTRSLCGPERSRAERSQPRQPEPQEGDSRRQQTQDPGSTDGKTSQPREPQAADRQRGRPNTEPRAEGKSEGREGAREQQSRAAE
Wall lizard Scfn2     ----------------------------------------RERENRRQDLHDP------EERSRESRAGNRRVSKP----------------RHTQSCAFS

                      1718                                                                                                1816
Wall lizard Scfn1     QWQNPVQGVSCKEESGCPGSMERQPEEGDRSSPQAREAQPPLEEVGEPGAERPPGGAASETNPEPSSQEKPGFLCGDKVPLVCNPLYQYLLAQKKQEQP
Wall lizard Scfn2     ----------------------------------------------------------------------------------CN---------------
```

C

```
                        1                                                                                                     103
Bearded dragon Scfn1    MPHLLNSICTIIGVFHKYAKWQGDCSTLNRKEMKELILQEFSQVMRNPHDPETVELIFQKLDFNENGLLDFNEYLLLLFQFTKACYSHLQPRECL-----SEG
Bearded dragon Scfn2    MPQLLDSICTIIGTFYKYAKWHEDCSTLNRKEMKKLIQKEFAIVLENQLDTKTAELIFQMLDINGDGMLDFNEYLLLLFNSAKACYRHLQSRECLLREEDGRG

                        104                                                                                                   206
Bearded dragon Scfn1    GPQEEG----RDRRQVHDGERVG--VRERPDSGRTRLRPIEGTTQGERRDCLPPSRERSSQAHDSEWRDDRGRDHSFRDAEDEYEGRPRRSGTWADAESHPQT
Bearded dragon Scfn2    PSQEEGHRDPRERCQLDDDEREGNYFRGRPDSGRTRLRPIEGIAQGERRDCLPPSRERSSQAHDSERRDDRGRDHSFRDAEDEYEGRPRRSGTWADAEPHPQT

                        207                                                                                                   309
Bearded dragon Scfn1    RRWEPERDQAGRHQLRRREAAGEEDRQRQSQELVPRSTGGRRHPAYGSPPGEGEERERYAPEGPERQEDRRHSWAPGTEHWLDEENLTRKRPSEPRVDEESRR
Bearded dragon Scfn2    RRWEPERDQAGRHQLRRREAAREEDRQRQSQELVPRSTGGRRHPAYGSPPGEGEERERYAPEGPERQEDRRHSWAPGTEHWLDEENLTRKRPSEPRVDEESRR

                        310                                                                                                   412
Bearded dragon Scfn1    RHPRAPEETRYDGGRCHPSSGSQEREDRRRRDSSDEQEMQDKLSSRDPERWQDRTHNHKPVDGADECFSLPRVGERRRSRTPEPERRERSWPSEPDRPDGERT
Bearded dragon Scfn2    RHPRAPEETRYDGGRCHPSSGSQEREDRRRRDSSDEQEMQDKLSSRDPERRQDRTHNHKPVDGADECFPLPRVGERRRSRTPEPERRDWSWPSEPDRPNGEWT

                        413                                                                                                   515
Bearded dragon Scfn1    GQRRGRTQYRGAEHGPDQPSGPEVREAERRLPHGDLTRCPRPWEPRTEVGAEAEDWRRSRDPEGGEQDRSRPRPRPYEPASTDSGRGWSQSYEPESREGLSRR
Bearded dragon Scfn2    GQRRGRTQYRGAEHGPDQPSGPEVREAERRLPHGDLTRCPRPWEPRTEVGAEAEDWQQSRDPEGGEQDRSRPRP-----------------------------

                        516                                                                                                   618
Bearded dragon Scfn1    PRRLDEDDTRRQRFHQEEEVDRRRPRFPEPEPCDYRRPERPEWEXXXRPRPYEPASTDSGRGWSQSYEPESREGLSRRPRRLDEDDTRRQRFHQEEEVDRRRP
Bearded dragon Scfn2    ---------------------------------------------------YEPASTDSGRGWSQSYEPESREGLSRRPRRLDEDDTRRQRFHQEEEVDRRRP

                        619                                                                                                   721
Bearded dragon Scfn1    RFPEPEPCDYRRPERPEWEDSQGRHQYDGAEPQERESYRRRPQLRDRDSREGERRRAPRTGSREDQGERPQPSYTQTRPTEQDQFSPSERSPRDCFQHRPQTY
Bearded dragon Scfn2    RFPEPEPCDYRRPERPEWEDSQGRHQYDGAEPQERESYRRRPQLRDRDSREGERTRAPRTGSRDDQRERPQP-------------------------------

                        722                                                                                                   824
Bearded dragon Scfn1    NYQSSEQGEREWRPNQSYDTEALDYGPSQMRAHDTDPRDGEHERQTLSHYAGPRDQRRTWPYERDPIGYDQQRRHPSYNTDATNTDRRGAHTRDGEQRDGEPQ
Bearded dragon Scfn2    ------------------------------------------------------------------------------------------HDEDDWRRD----

                        825                                                                                                   927
Bearded dragon Scfn1    RRTPPYNTDAPSVDRRQTQACEIGPRDWEQQKQTQGYDTRQRGSEPRQTITHEVDSRDTERQRTLSHGPDARNVDLRRTQKKAVDPRDCGPHDTDAGGADQRR
Bearded dragon Scfn2    ---------------RQAELCE---------------DTGVR----------------------------------------------CGP------------

                        928                                                                                                   1030
Bearded dragon Scfn1    TQRGDPRTNAEQRQREGDQQQTQSRDTDPSHEDQQCRTSSYDGDLKGVEQRQTQTCHVDPKNDEQQRRTPVHETVTREAEQRTEDVAPEDGEHQRQTPIYGNG
Bearded dragon Scfn2    ----------------------QTRESEPSN--LQCNR---------------------PERGVQELR----------GDRRGRQQSPDGEDERRQQPQYGSD

                        1031                                                                                                  1133
Bearded dragon Scfn1    QRGTERRGTQTGEPDPRACDHQRRTLVSDRRDGGQRQIQKPEDPKDGQQQEETLKSDAHARGIDQGRTQTEDIDPKCGEEQRLILPHDSATINADQRQNQSQD
Bearded dragon Scfn2    SGNGEQRTAQAQEP----------FLKEDVPRSQPRYESKPMQ-REGERR-------ALAR------------DPARREDNRT-------------RQQQHQD

                        1134                                                                                                  1236
Bearded dragon Scfn1    VGSRDSAQQRQIPSHGTDQKAAEKRQTQAFRAEPRTSGEQRQTPPPRGPDLREGDQSQTQVREVDPKGDDAQRLCKPHDAEQKGTKQVQGIPSDVTEGEPGRS
Bearded dragon Scfn2    ---DDPEEERD-----TQQRALHER-------EPARRGDSLSRPRSYKPNSRERE-----------------------------------------------S

                        1237                                                                                                  1339
Bearded dragon Scfn1    RWTACDSGTKSNVWMGPGEPGSEAARACPDPTLSSMPQEGERSRVCPHGAVPMTSQPSRYQLHAPGATKGALGGQRPPEPAEAPQREGGQESSQLQKTKLEQA
Bearded dragon Scfn2    EW---------------GRP-----QAC-----SAEPMEGER---------------------------------RQPRQTDLDQSEGASPAELLSR------

                        1340                                                                                                  1442
Bearded dragon Scfn1    ARRSHCLRGPELRRPERGRPQPQSTEPQEGEPRMQKAQEPGSSGSTPTATTQPHEAQSTRSQRGRPQVREARPEPRAEGKPGASQLPERGGIQDAPGGPEQLQ
Bearded dragon Scfn2    ---------------HRGQEGPRPSE--------------AGSGGRDRNRQQAHESSSREPERRAPQL-----------------------------------

                        1443                                                                                         1536
Bearded dragon Scfn1    PVVQRTSHDGGGGQPAPTPPQSEGDDESSQQARDPQPPLEEESQPGAAEGQSHPKSLESDASQGKPGDLCEAKTPVVCNPLYEYLVAQKKQEQL
Bearded dragon Scfn2    -----------------------GDPKRSETGQSKKMYLG----PGAVEEEA----------------------------------SRRRFLLH
```

D

```
                1                                                                                                        106
Cobra Scfn1     MSYFLDSVCTIVGIFHKYARCQDGNLALNRREMKALIQKEFAEVLENPCDPQTIELTFKLLDVNGDSLVDFNEYLIFVFQIAKGCYRYLQPRE-YLLRDESSRALH
Cobra Scfn2     MAGLVDSICTIIAVFHKYADRKSESSSMKRRQMKRLIQKEFGDVLENPRDPQIVKLTFQLLDVNGDNRVDFNEFLFLIFEMATACYSVWHPRECFSYNEERRRAVP

                107                                                                                                      212
Cobra Scfn1     EGEAGGSKRGDHQLQDGERRGDYVHERQGLDGTFLHSTEEGSRGELVGRYLPIEEVEEEDSNFEGHEPKLRDGDRRDHPSQEHQEREPEQQWREPKEWEDIETPEP
Cobra Scfn2     DEEPRGDESNRREFLGEDRRRDDVRERRGADRTPLPSMEEGRRGELSS-----ELREEVDRRFEGHDRELRDGDRRDRPSRELQEREPEQRRLEPKEWEDVDIPEP

                213                                                                                                      318
Cobra Scfn1     CQWRQQESTLEEDSPRQSRELVRRNDNDRREARGLLDRDEEDLYSPIVPSRREEMRQHRGQETE-----GRLFRYGSQARVD----HTDYDVERSWPSHEWQEVED
Cobra Scfn2     RQRRQRESAVEEDSPRQSRELVRRNDNDRREARGLLDRDEEDLYSPTVISRREDERQRQGRETEHQEAEGRLFRHGSQARVDTRNHRPEYDVERGQPSRERREGED

                319                                                                                                      424
Cobra Scfn1     GRRELEMHNERRSHAFEHRVADDPRDRIHHRSPVEEYEEHHPLSTEGERRSQFHEDDHRESERRRQGRSVFQSRKDVRRAFRAEEEEVRRSERRHDEDEWNRPQPR
Cobra Scfn2     GRRELEMHNERRSRAFEPRVADDRRDRIRHRSPVEEYEEHRPLSREGERRSQSYEDDHRESESRRQGRSVFQSREDVRRAIRAEEEEVRRSERRHVEDEWSRPRPR

                425                                                                                                      530
Cobra Scfn1     EASRRSEVQDLERRERETRRPYSSELKNMDSPSRSHESESREEVRRSQHPEDIVQRRREFSREDTRVTEVRRRRSQSLEPVARDIRYVRSQRYEEPRSNDQRRPHN
Cobra Scfn2     EASRRPEVQDLERRERETRRPYSSELENMDRPSRSHESEGREAVRRSQRPEDVERRRQELSWEETRATESRGRRPQSQEPEPRDPKYDRSQSYEEPRRDDQRRPRN

                531                                                                                                      636
Cobra Scfn1     YEGRSLERDVARRRRLQLRESPKREDNQERQWYYEHETWDSERDGRRCQLRYSEPREREMRRYQVVDSRVDEREQSREAVTRPTQWDRFSPSDSPGEYDQSGQQIY
Cobra Scfn2     YEVRSQERDAERRRRLQPRELVEREDNQERQ-HYERESRDRERDGRRAMSLEAETRDEVRRRPQESASRYGERERLPEGDAR------------------------

                637                                                                                                      742
Cobra Scfn1     ESRHTLQREGRGQRYDRLSNGQRQIWAIDIAQRESEGQRRSNVEVRIVDQFRGVDHRESEHQRRTSIHDSDDENTDQRQTQTYEASSREDEDQRRTSSHDSGTRQI
Cobra Scfn2     -----MRRTGR-----------------DMASPSDAGRRELD---------------------------------------------------------PSSPQQI

                743                                                                                                      848
Cobra Scfn1     NQRRTPTYEVGPKDDQRRRRMPSRESVDQRWRTHTQTDLAEDGQKPSNPYETDPRDGEQQRTVQSSDPNFRDTEATQHNMGDSTQPREATSLSNQTADREFDLRRD
Cobra Scfn2     SRRRAP-----------------------------------------------------------------------------------------------ELRRS

                849                                                                                                      954
Cobra Scfn1     QRPPESGESQPSRKPGQSKAEQSPVSPYEQHQPTRESQRLSGPELRGPQRRWLPHHQTEAPEEAGVDQVQRKVGSPVSKRAISQPSERQITEGQESRIQGYHRTPV
Cobra Scfn2     E----------------------------------------------------PHSFMPA------------------------------TKG-------------

                955                                                                                                   1057
Cobra Scfn1     SQAEGESELSLDGEGDKAQEPAALNQQQVHREGEVPQPVAEEPKVEEDDGSGSWAQESQPPLEDDDQQAITEKPSSSLVESKSCVICNPLYEYLLAQKKQEQP
Cobra Scfn2     -------------------------------------------------------------------------------------------------------
```

Supplementary Figure S11. Phylogenetic tree and pairwise sequence alignments of SFTPs. (A) A phylogenetic analysis of sauropsidian SFTPs was performed with PhyML (100 replicates). Bootstrap values >50 are indicated. Amino acid sequences of scaffoldin 1 (Scfn1) and 2 (Scfn2) of the wall lizard **(B)**, komodo dragon **(C)** and cobra **(D)** were aligned. Sequence identity is indicated by red fonts. Stretches of more than 30 amino acid residues with more than 90% sequence identity to the other scaffoldin of the same species are highlighted by yellow shading. Note that there are no such long stretches of sequence identity among Scfn1 proteins of different species and among Scfn2 proteins of different species. Species: tuatara (Sp, *Sphenodon punctatus*), Japanese gecko (Gj, *Gekko japonicus*), common wall lizard (Pm, *Podarcis muralis*), anole lizard (Ac, *Anolis carolinensis*), Western painted turtle (Cp, *Chrysemys picta bellii*), Komodo dragon (Vk, *Varanus komodoensis*), bearded dragon (Pv, *Pogona vitticeps*), Burmese python (Pb, *Python bivittatus*) and king cobra (Oh, *Ophiophagus hannah*). p, partial sequence; SFTP, S100 filaggrin-type protein; Crnn, Cornulin.
